# Screening of Anti-Inflammatory Activity and Metabolomics Analysis of Endophytic Fungal Extracts; Identification and Characterization of Perylenequinones and Terpenoids from the Interesting Active Alternaria Endophyte

**DOI:** 10.3390/molecules28186531

**Published:** 2023-09-09

**Authors:** Rosella Spina, Armelle Ropars, Sihem Bouazzi, Safa Dadi, Pascal Lemiere, François Dupire, Afra Khiralla, Sakina Yagi, Jean-Pol Frippiat, Dominique Laurain-Mattar

**Affiliations:** 1 Université de Lorraine, INRAE, LAE, F-54000 Nancy, France; dominique.mattar@univ-lorraine.fr; 2 Université de Lorraine, SIMPA, F-54000 Nancy, France; armelle.ropars@univ-lorraine.fr (A.R.); jean-pol.frippiat@univ-lorraine.fr (J.-P.F.); 3 Université de Lorraine, CNRS, L2CM, F-54000 Nancy, France; bouazzi-sihem@hotmail.com (S.B.); dadi.safa@gmail.com (S.D.); pascal.lemiere@univ-lorraine.fr (P.L.); francois.dupire@univ-lorraine.fr (F.D.); 4 Botany Department, Faculty of Sciences and Technologies, Shendi University, Shendi 11111, Sudan; aafraa21@hotmail.com; 5 Department of Botany, Faculty of Science, University of Khartoum, Khartoum 11115, Sudan; sakinayagi@gmail.com

**Keywords:** endophytic fungi, anti-inflammatory activity, molecular network, GNPS, *Alternaria alternata*, perylenequinones

## Abstract

Patients suffering from inflammatory chronic diseases are classically treated with anti-inflammatory drugs but unfortunately are highly susceptible to becoming resistant to their treatment. Finding new drugs is therefore crucial and urgent and research on endophytic fungi is a promising way forward. Endophytic fungi are microorganisms that colonize healthy plants and live within their intercellular tissues. They are able to produce a large variety of secondary metabolites while allowing their host to stay healthy. A number of these molecules are endowed with antioxidant or antimicrobial as well as cytotoxic properties, making them very interesting/promising in the field of human therapy. The aim of our study was to investigate whether extracts from five endophytic fungi isolated from plants are endowed with anti-inflammatory activity. Extracts of the endophytic fungi *Alternaria alternata* from *Calotropis procera* leaves and *Aspergillus terreus* from *Trigonella foenum-graecum* seeds were able to counteract the lipopolysaccharide (LPS) pro-inflammatory effect on THP-1 cells differentiated into macrophages. Moreover, they were able to induce an anti-inflammatory state, rendering them less sensitive to the LPS pro-inflammatory stimulus. Taken together, these results show that these both endophytic fungi could be interesting alternatives to conventional anti-inflammatory drugs. To gain more detailed knowledge of their chemical richness, phytochemical analysis of the ethyl acetate extracts of the five endophytic fungi studied was performed using HPTLC, GC-MS and LC-MS with the Global Natural Products Social (GNPS) platform and the MolNetEnhancer tool. A large family of metabolites (carboxylic acids and derivatives, steroid derivatives, alkaloids, hydroxyanthraquinones, valerolactones and perylenequinones) were detected. The purification of endophytic fungus extract of *Alternaria alternate,* which diminished TNF-α production of 66% at 20 µg/mL, incubated one hour before LPS addition, led to the characterization of eight pure compounds. These molecules are altertoxins I, II, III, tricycloalternarenes 3a, 1b, 2b, anthranilic acid, and *o*-acetamidobenzoic acid. In the future, all these pure compounds will be evaluated for their anti-inflammatory activity, while altertoxin II has been shown in the literature as the most active mycotoxin in terms of anti-inflammatory activity.

## 1. Introduction

The incidence rate of chronic inflammatory disease is increasing and now constitutes a world health problem due to some factors such as the ageing population, lack of exercise, obesity, unhealthy food, sleep disturbance and environmental factors such as pollution [1,2,3,4]. Patients are treated with corticosteroids and/or non-steroidal anti-inflammatory drugs. 

Patients suffering from inflammatory bowel disease or rheumatoid diseases such as arthritis, lupus or vasculitis are classically treated with steroidal anti-inflammatory drugs to diminish inflammation and pain [5,6]. In addition, patients suffering from other diseases such as lymphoid malignancies or asthma are also classically treated with glucocorticoids [5,7]. However, due to long-term use and/or high doses, a significant portion of all these patients may have to contend with two major risks: corticosteroid drug resistance and/or deleterious effects on organs, which aggravate their health problems which in turn increase their co-morbidity factors. Corticoid resistance is due to modifications in transduction signaling pathways linked to GRα, the corticosteroid receptor, as well as its decreased protein expression, but factors such as GRβ expression, as well as genetic factors, are also involved in this resistance [8,9,10]. Non-steroidal anti-inflammatory drugs (NSAIDs) have the ability to inhibit cyclooxygenase enzymes COX-1 and COX-2. COX-1 is expressed in most tissues in basal conditions and is responsible for prostaglandin production. These eicosanoids are in charge of different homeostatic functions such as maintaining the gastric mucosa integrity, regulating renal blood flow and participating in normal platelet function. COX-2 is induced in stressed cells and produces prostaglandins linked to fever, pain and/or inflammation. In view of their importance, resistance to NSAIDs can also cause serious health problems such as renal and/or cardiovascular failures, gastrointestinal bleeding and osteoporosis. Moreover, the compounds can promote the onset of diabetes and/or high blood pressure [11,12].

The search for new classes of anti-inflammatory molecules is therefore crucial, and finding new molecules which could be complementary or maybe even an alternative to the classical anti-inflammatory molecules will be useful not only for humans but also for other animals’ health.

Endophytes are microorganisms that can colonize the internal tissues of healthy plants without showing disease symptoms, and they form symbiotic relationships with their host plants. Endophytic fungi have been reported to protect their host plants by producing various metabolites with antiviral, antifungal and antibacterial properties [13,14]. The fungal kingdom is very interesting to study, as fungi can be beneficial to humans but also toxic or pathogenic for them. In fact, endophytic fungi have been identified as a source of a wide variety of specialized metabolites with interesting, pharmacologically active structures that could offer specific medicinal and agrochemical applications such as, for example, the discovery of the penicillin molecule from the fungus *Penicillium notatum* in 1922 [15,16]. 

In this research area, the endophytic fungal compounds seems also contain some promising types of metabolites, with an extensive body of literature describing their antioxidant, anti-inflammatory, neuroprotective and anticancer properties [15,17,18,19,20] as well as biotechnological and agronomic potentials [21]. In addition, their abundance, their diversity and ease of cultivation exhibit considerable advantages. Moreover, stresses modify their chemical profile increasing diversity and, for some, metabolite concentration. The advantages of fungal endophytes are: diversity, abundance, easiness of culture, stresses modify their chemical profile increasing diversity and for some, concentration. 

In a previous work, we achieved the isolation of thirty strains of fungal endophytes from five plants of Sudanese origin [22]. The potential antioxidant activity of their crude extracts was measured via DPPH radical scavenging assay. For some extracts, a high antioxidant activity related to their phenol content was observed.

Here, we investigated if these endophytic fungal extracts endowed with a high or moderate anti-oxidant activity were also able to counteract the LPS pro-inflammatory effect on THP-1 cells differentiated in macrophages. 

Moreover, we conducted a metabolomics screening (HPTLC, GC-MS and LC-MS with the Global Natural Products Social (GNPS) platform and the MolNetEnhancer tool) of these extracts to search the families of known molecules and compounds and improve the knowledge of the chemistry of these unstudied fungal endophytes extracts from original medicinal plants.

Finally, we purified the extract showing the strongest anti-inflammatory activity, and we isolated their compounds. 

## 2. Results

On the basis of our previous study, five endophytic fungi strains were chosen for their interesting potential antioxidant effects: *Alternaria alternata* isolated from the leaves of *Calotropis procera* (ethyl acetate extract A) has showed a total antioxidant capacity with IC_50_ values of 236.0 ± 8.3 µg/mL; *Aspergillus terreus* isolated from the stems of *Calotropis procera* (ethyl acetate extract B) has showed a total antioxidant capacity with IC_50_ values of 58.0 ± 0.4 µg/mL; two *Aspergillus terreus* strains isolated from the seeds of *Trigonella foenum-graecum* (ethyl acetate extracts C and D, isolated respectively in 2014 and in 2015) have showed a total antioxidant capacity with IC_50_ values of 18.01 ± 0.1 µg/mL; *Cladosporium cladosporioides* isolated from the leaves of *Vernonia amygdalina* (ethyl acetate extract E) has showed a total antioxidant capacity with IC_50_ values of 480.0 ± 3.9 µg/mL [22].

### 2.1. Cytotoxicity and Anti-Inflammatory Activity of the Five Fungal Endophytic Extracts

THP-1 cells were differentiated into macrophages and used to analyze the potential anti-inflammatory effects of the five fungal endophytic extracts on LPS-induced pro-inflammatory response. Indeed, LPS is classically used in in vitro studies to promote a pro-inflammatory response, which is notably characterized by a strong increase in production of pro-inflammatory cytokine such as TNF-α by THP-1 cells. Two conditions were used to study two different cellular states. The first condition consisted of pre-incubating differentiated THP-1 cells with 100 ng/mL of LPS to induce a pro-inflammatory state and, one hour later, adding one fungal endophytic extract at different concentrations (LPS + fungal endophytic extract). The purpose of this approach was to determine if the extract was able to counteract LPS-induced pro-inflammatory response. The second condition was the opposite, consisting of incubating THP-1 cells with one fungal endophytic extract, to potentially promote an anti-inflammatory cellular state, and one hour later to add 100 ng/mL of LPS (fungal endophytic extract + LPS). As cell viability could be decreased using fungal endophytic extracts and/or ethanol used to dissolve these extracts and thus indirectly decrease TNF-α production, we first had to evaluate the effect of different concentrations of endophytic fungal extracts/ethanol on THP-1 cells viability in the presence or absence of LPS. In both cases, cell viability and TNF-α production were analyzed after 24 h of treatment.

#### 2.1.1. Cytotoxicity of the Five Fungal Endophytic Extracts on Differentiated THP-1 Cells

THP-1 cell viability was detected using the crystal violet assay, and for each fungal endophytic extract, we determined which concentrations had no impact on THP-1 cell viability (cell viability > 97%) and could then be used for further experiments on THP-1 cells. 

The five fungal endophytic extracts can be divided into three groups. For fungal endophytic extracts A and D, cytotoxic effect on THP-1 cells appeared at 40 µg/mL; for fungal endophytic extracts B and E, at 60 µg/mL; and no cytotoxicity was observed for fungal endophytic extract C until 60 µg/mL (Table 1). We were unable to test higher concentrations due to the appearance of ethanol cytotoxicity from the concentration equivalent to 100 µg/mL LPS used at 100 ng/mL and added one hour before or after the fungal endophytic extract had no impact on THP-1 cell viability (Table 1).

This means that for anti-inflammatory activity studies, fungal endophytic extracts A and D could be tested only at 5, 10 and 20 µg/mL; B and E at 5, 10, 20 and 40 µg/mL; and C at 5, 10, 20, 40 and 60 µg/mL.

#### 2.1.2. Anti-Inflammatory Activity of the Five Fungal Endophytic Extracts on Differentiated THP-1 Cells

**Figure 1:**
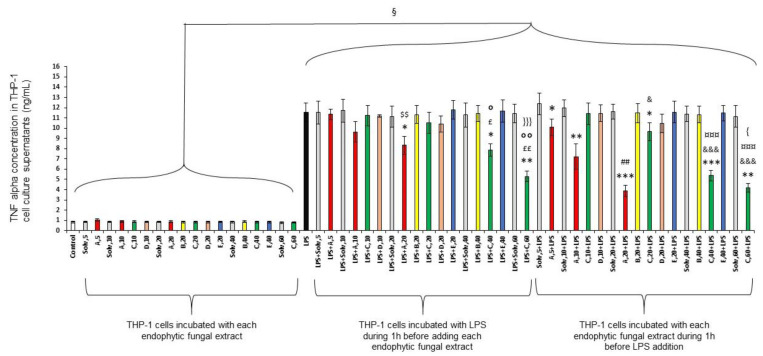
Endophytic fungal extracts are able to reduce the TNF-α production of differentiated THP-1 cells. Five concentrations (5, 10, 20, 40 and 60 µg/mL) of endophytic fungal extracts were evaluated based on their absence of cytotoxicity on THP-1 cells. Ethanol used to dissolve these extracts was also used as a reference. Differentiated THP-1 cells were incubated for 24 h either with solvent/endophytic fungal extract alone or with 100 ng/mL of LPS added one hour before or after the addition of solvent/endophytic fungal extract. Cell culture supernatants were then collected and used to quantify TNF-α production via ELISA. “Control” corresponds to a THP-1 cell culture in which no product was added. “Solv” corresponds to ethanol. “LPS + solvent/endophytic fungal extract,5” indicates that LPS was added before solvent/endophytic fungal extract at 5 µg/mL while “solvent/endophytic fungal extract,5 + LPS” indicates that the solvent/endophytic fungal extract at 5 µg/mL was added before LPS. This figure is representative of six independent experiments performed in triplicate. Data are presented as mean ± S.D. *t*-tests were used to identify statistically significant differences. ^§^ *p* < 0.05 vs. conditions without LPS. * *p* < 0.05 vs. “LPS+solv” or “solv+LPS”; ** *p* < 0.01 vs. “LPS+solv” or “solv+LPS”; *** *p* < 0.001 vs. “LPS+solv” or “solv+LPS”; ^$$^ *p* < 0.01 vs. “LPS+A,10”; ^£^ *p* < 0.05 vs. “LPS+C,10”; ^££^ *p* < 0.01 vs. “LPS+C,10”; ° *p* < 0.05 vs. “LPS+C,20”; °° *p* < 0.01 vs. “LPS+C,20”; ^}}}^ *p* < 0.001 vs. “LPS+C,40”; ^##^ *p*< 0.01 vs. “A,10+LPS”; ^&^ *p* < 0.05 vs. “C,10+LPS”; ^&&&^ *p* < 0.001 vs. “C,10+LPS”; ^¤¤¤^ *p* < 0.001 vs. “C,20+LPS”; ^{^ *p* < 0.05 vs. “C,40+LPS”.

As expected, TNF-α secretion by THP-1 cells increased strongly when LPS was added to cell culture (indicated by “£” in Figure 1). 

THP-1 cells differentiated into macrophages produced less TNF-α when endophytic extracts A or C were added one hour after or before LPS. This inhibition was concentration-dependent. More precisely, endophytic extract A added at 10 µg/mL and 20 µg/mL one hour after LPS diminished TNF-α production, respectively, by 17 and 28%; under the same conditions, endophytic extract C used at 20, 40 and 60 µg/mL diminished this cytokine production by 10, 32 and 54%, respectively. 

If these extracts were incubated one hour before the LPS addition, their inhibitory effect on TNF-α production was always stronger than under the condition “LPS + endophytic extract”. Indeed, endophytic extract from A added at 5, 10 µg/mL and 20 µg/mL diminished TNF-α production by 18%, 37 and 66%, respectively, and endophytic extract from C, used at 20, 40 and 60 µg/mL diminished this cytokine production by 17, 54 and 64%, respectively.

The other fungal endophytic extracts B, D and E failed to inhibit the pro-inflammatory effect of LPS regardless of the concentration and the condition.

### 2.2. High Performance Thin Layer Chromatography (HPTLC)

To discover the chemical composition of the five extracts of fungal endophytes, the initial screening was carried out via HPTLC analysis. 

**Figure 2:**
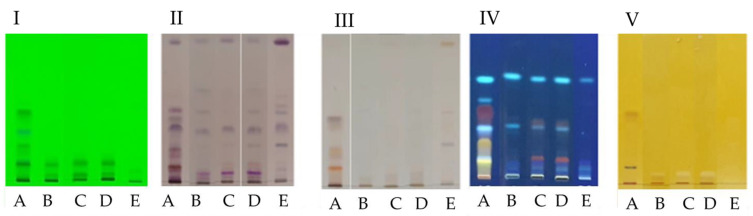
HPTLC screening of extracts A, B, C, D and E. The observation was carried out via observation under an ultra-violet lamp at 254 nm (**I**), visible light after using anysaldehyde reagent (**II**) for terpenoid detection, visible light after using 10% H_2_SO_4_ reagent (**III**) for organic compounds detection, ultra-violet lamp at 365 nm after using Neu’s reagent (**IV**) for flavonoid detection, visible light after using Dragendorff reagent (**V**) for alkaloid detection.

Observation of the HPTLC plates under UV at 365 nm and 254 nm (Figure 2I) revealed that overall, the extracts were rich in secondary metabolites. Extracts B, C and D showed similar phytochemical profiles remarkable for the presence of 2 orange spots with Rf = 0.11 and Rf = 0.33, which were not present in the other extracts. After using four different spraying reagents such as sulfuric anysaldehyde, sulfuric acid, Neu’s reagent and Dragendorff reagent (Figure 2II, III, IV and V respectively), it appeared that all fungal endophyte extracts contained terpene compounds. The extracts A, B, C and D showed orange spots that indicate the presence of the alkaloids. Neu’s reagent made possible to highlight the presence of flavonoids in extracts A, B, C and D. 

Extract A was the one with the greatest richness in secondary metabolites (Figure 2). A large majority of these compounds are at an Rf between 0 and 0.6. It is possible to observe about ten spots corresponding to terpene compounds at Rf between 0 and 0.5. Polyphenols were also observed in the form of yellow (Rf = 0.08) and orange (Rf = 0.46) spots.

### 2.3. Gas Chromatography Coupled to Mass Spectrometer (GC-MS)

In total, seven compounds in extract A, six in extracts B and C, seven in extract D and nine in extract E were identified using GC-MS (Table 2). The analysis showed the presence of different classes of molecules, including fatty acids, sterols, nitrogen derivatives and statins. 

Fatty acids, such as linoleic, oleic, stearic and palmitic acids were present in the extracts. 

The cyclo Pro-Leu was identified in extracts A and D and the cyclo Phe-Pro only in extract E.

Extracts B, C and D were derived from endophytes of the genus Aspergillus which were isolated from different plants. The profile of extracts B and D were very similar, while extract C was different; for example, palmitic and stearic acids were not detected in extract C, whereas 3-*t*-pentylcyclopentanone, methyl 2-nonynoate and 11,14-eicosadienoic acid, methyl ester were detected. 

These 3 fungal endophytes are the only ones in which lovastatin and hippuric-benzaldehyde azalactone have been identified.

In order to identify other molecules, derivatization via silylation was carried out. In total, ten compounds in extract A, four for extract B, six in extract C, five in extract D and eleven in extract D were identified (Table 3).

After silylation, the GC-MS analysis confirmed the previous results, such as the presence of linoleic acid in all extracts and the presence of lovastatin in extracts B, C and D. Itaconic acid was detected in extract C. Benzoic acid was detected in extracts C and E. Among the other identified molecules, there were dicarboxylic acids such as succinic acid and malic acid in extracts A and E and fumaric acid in extract E. 

### 2.4. Liquid Chromatography Coupled with Mass Spectrometry in Tandem (LC/MS-MS) and Molecular Network (MN) of the Fungal Endophytic Crude Extracts

Our identification and annotation of chemical compounds is based on the comparison of commercial standards, the GNPS (Global Natural Products Social Molecular Networking) libraries and data from the lit. 

GNPS organizes and visualizes MS/MS data based on spectral similarity based on the presence of homologous MS/MS fragments or homologous neutral loss. MolNetEnhancer Workflow is used in the description for chemical class annotation of molecular networks, and Cytoscape software 3.8.0 (U.S. National Institute of General Medical Sciences, Bethesda, MD, USA) [23] is used for their visualization. MolNetEnhancer reveals the chemical type of nodes in the GNPS molecular network and provides chemical class annotations. In particular, MolNetEnhancer can integrate the output of several metabolome mining and annotation tools, including GNPS molecular networking and in silico annotation tools, as well as identify molecular families, subfamilies and subtle structural differences between family members.

All strains were first analyzed with GNPS and then with MolNetEnhancer Workflow.

MolNetEnhancer increased chemical structural information about composition in metabolites from the crude extracts. 

**Figure 3:**
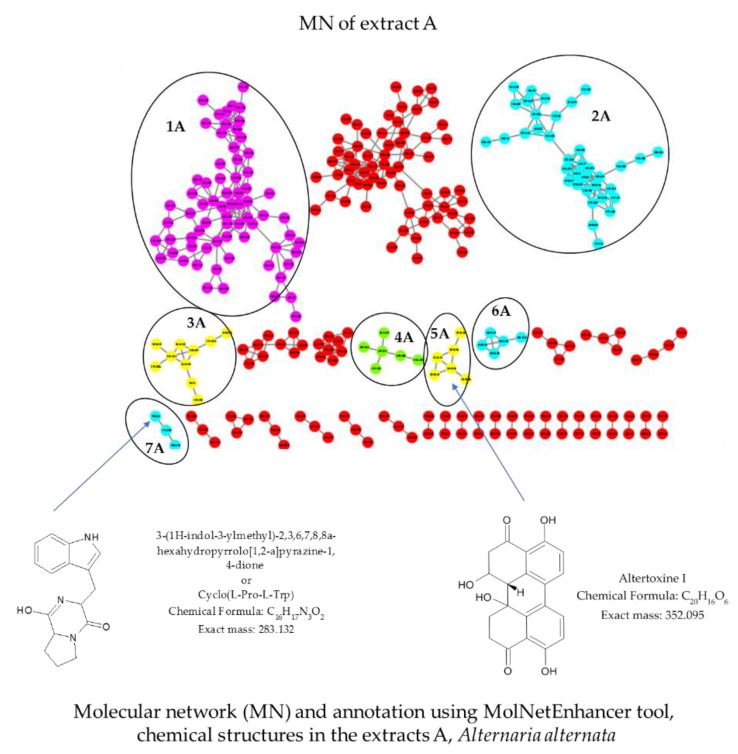
Molecular network and annotation of extract A created using MolNetEnhancer. Each node represents a precursor ion (MS), and edge thickness between nodes indicates similarity in MS/MS fragmentation patterns. The node color represents the structural annotation at the class/subclass level. Annotated class/subclass: 1A—indoles and derivatives/naphthoylindoles; 2A, 6A and 7A—carboxylic acids and derivatives/amino acids, peptides and analogs; 3A—benzene and substituted derivatives/purines and purine derivatives; 4A—steroids and steroid derivatives/steroidal alkaloids; 5A—perylenequinones. Unidentified clusters are in red or not circled.

Molecular network (MN) analysis of extract A (Figure 3) revealed the presence of five classes/subclasses of chemical compounds. Cluster 1A corresponded to indoles and derivatives/naphthoylindoles. Clusters 2A, 6A and 7A corresponded to carboxylic acids and derivatives/amino acids, peptides and analogs. Cluster 3A corresponded to benzene and substituted derivatives/purines and purine derivatives. Cluster 4A corresponded to steroids and steroid derivatives/steroidal alkaloids. Cluster 5A corresponded to perylenequinones.

In cluster 5A, the ion peak at *m*/*z* 353.101 [M + H]^+^ was identified as Altertoxin I, a perylenequinone compound, which matches with the molecular formula of C_20_H_16_O_6_.

Among them, in cluster 7A, the ion peak at *m*/*z* 284.140 [M + H]^+^ was identified as 3-(1H-indol-3-ylmethyl)-2,3,6,7,8,8a-hexahydropyrrolo[1,2-a]pyrazine-1,4-dione or Cyclo(L-Pro-L-Trp), which matches the molecular formula of C_16_H_17_N_3_O_2_.

**Figure 4:**
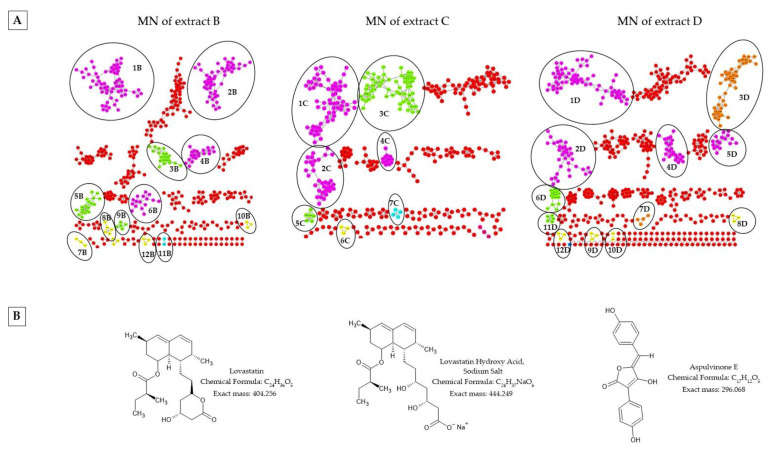
(**A**) Molecular network and annotation using MolNetEnhancer of extracts B, C and D. Each node represents a precursor ion (MS) and edge thickness between nodes indicates similarity in MS/MS fragmentation patterns. The node color represents the structural annotation at the class/subclass level. Annotated class/subclass clusters: 1B, 2B, 1C, 2C, 1D, 2D—Lactones/Delta valerolactones; 4B, 4C, 4D—Pyrimidodiazepines; 10B, 6C, 9D—Phenols/1-Hydroxy-2-unsubstituted benzenoids; 8B, 12B, 8D, 10D, 12D—Anthracenes/Hydroxyanthraquinones; 9B, 5C, 11D—Fatty Acyls/Fatty acid esters; 3B, 3C—Steroids and steroid derivatives/bile acids, alcohols and derivatives; 5B, 6D—Prenol lipids/Diterpenoids; 11B, 7C—Carboxylic acids and derivatives/Amino acids, peptides and analogs; 3D—Lupin alkaloids/Sparteine, lupanine and related alkaloids; 5D—Pyridines and derivatives/Hydropyridines, 6B—Lactones/gamma butyrolactones; 7B—Benzene and substituted derivatives/Diphenylethers; 7D—Phthalide isoquinolines. (**B**) Common chemical structures in the extracts B, C and D from *Aspergillus terreus*. Unidentified clusters are in red or not circled.

Molecular network (MN) analysis of extracts B, C and D (Figure 4) have been compared because the 3 extracts, which were isolated from two different plant species, came from the same genus and species of endophytic fungus (*Aspergillus terreus*).

Network analysis revealed the presence of 12 classes/subclasses of chemical compounds annotated such as: lactones/delta valerolactones (clusters 1B, 2B, 1C, 2C, 1D and 2D) corresponding to statin compounds; pyrimidodiazepines (clusters 4B, 4C and 4D); anthracenes/hydroxyanthraquinones (clusters 8B, 12B, 8D, 10D and 12D); fatty acyls/fatty acid esters (clusters 9B, 5C and 11D); steroids and steroid derivatives/bile acids, alcohols and derivatives (clusters 3B and 3C); prenol lipids/diterpenoids (clusters 5B and 6D); carboxylic acids and derivatives/amino acids, peptides and analogs (clusters 11B and 7C); lupin alkaloids/sparteine, lupanine and related alkaloids (cluster 3D); pyridines and derivatives/hydropyridines (cluster 5D); lactones/gamma butyrolactones (cluster 6B); benzene and substituted derivatives/diphenylethers (7B); phthalide isoquinolines (cluster 7D).

The molecular network (MN) revealed that the three extracts (B, C and D) had the following classes/subclasses in common: lactones/delta valerolactones lactones, pyrimidodiazepines, phenols/1-hydroxy-2-unsubstituted benzenoids and fatty acyls/fatty acid esters.

Lovastatin was annotated in the lactones/delta valerolactone and lactone clusters; according to the peak ion *m*/*z* 405.256, [M + H]^+^ was present, which matched the molecular formula of C_24_H_36_O_5_. The peak ion *m*/*z* 445.253 as [M + Na]^+^ corresponded to lovastatin hydroxy acid sodium salt with molecular formula C_24_H_35_O_6_.

These compounds were present in the three *Aspergillus terreus* extracts, and the lovastatin structure was confirmed via comparison with the commercially available standard and GC-MS analysis.

In extract B, we observed the peak ions *m*/*z* 437.289, *m*/*z* 407.272 and *m*/*z* 447.268 as [M + H]^+^, corresponding to lovastatin-related compounds according MS/MS spectra.

In the clusters 8B, 8D and 10D, phenols/1-hydroxy-2-unsubstituted benzenoids were identified. In these networks, we observed the ion corresponding to Aspulvinone E, a yellow pigment found in Aspergillus, with peaks ions *m*/*z* 297.071 as [M + H]^+^ which matched the molecular formula of C_17_H_12_O_5_ (calculated (calcd.) for [M + H]^+^ 297.075).

Several classes/subclasses were only common in two extracts. This was the case for anthracene/hydroxyanthraquinone networks (8B, 12B, 8D, 10D and 12D), uniquely present in extracts B and D. Questinol and questin were annotated in the clusters 8B–8D and 12B–12D, with peak ions at *m*/*z* 301.063 and 285.072 as [M + H]^+^ which matched the molecular formula of C_16_H_12_O_6_ ([calcd. for [M + H]^+^ 307.070) and C_16_H_12_O_5_ (calcd. for [M + H]^+^ 285.076) respectively. Emodin (C_15_H_10_O_5_, calcd. for [M + H]^+^ 271.060) was identified only in cluster 10D, with 271.054 as [M + H]^+^.

**Figure 5:**
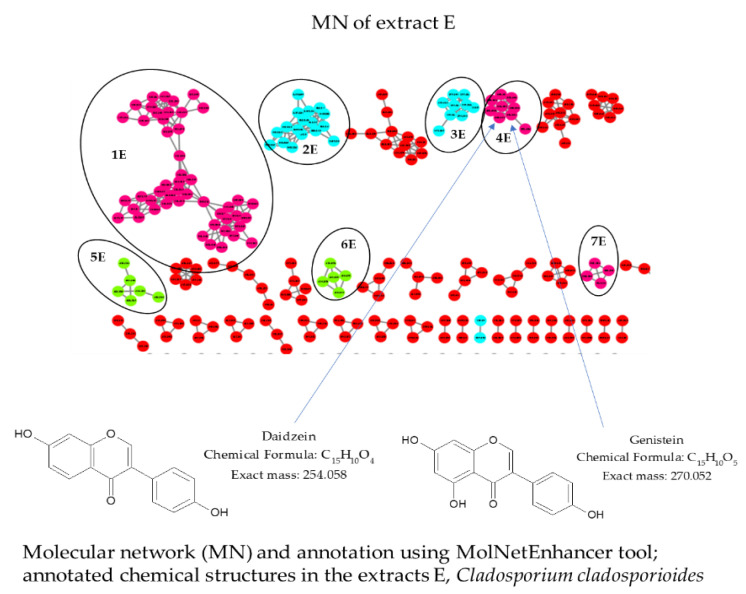
Molecular network and annotation of extract E using MolNetEnhancer tool. Each node represents a precursor ion (MS) and edge thickness between nodes indicates similarity in MS/MS fragmentation patterns. The node color represents the structural annotation at the class/subclass level. Annotated class/subclass: 1E—Flavonoids/Flavones, 2E—Carboxylic acids and derivatives/Dipeptides, 3E—Carboxylic acids and derivatives/Alpha amino acids and derivatives, 4E—Isoflavonoids/Isoflavones, 5E—Steroids and steroid derivatives/Ergostane steroids, 6E—Prenol lipids/Sesquiterpenoids, 7E—Tannins/Hydrolyzable tannins. Unidentified clusters are in red or not circled.

Molecular network (MN) analysis of extract E revealed the presence of different classes/subclasses of chemical compounds (Figure 5). Cluster 1E corresponded to flavonoids/flavones. Cluster 2E corresponded to carboxylic acids and derivatives/dipeptides. Cluster 3E corresponded to carboxylic acids and derivatives/alpha amino acids and derivatives. Cluster 4E corresponded to isoflavonoids/isoflavones. Cluster 5E corresponded to steroids and steroid derivatives/ergostane steroids. Cluster 6E corresponded to prenol lipids/sesquiterpenoids, and cluster 7E corresponded to tannins/hydrolyzable tannins.

In cluster 4E, the peak ion of *m*/*z* 271.053 as [M + H]^+^ (theoretical *m*/*z* 271.060) and the peak ion of *m*/*z* 255.06 as [M + H]^+^ were identified as genistein and daidzein (C_15_H_10_O_4_, calcd. for [M + H]^+^ 255.065), respectively.

### 2.5. Purification of Extract a from Alternaria alternata and Characterization of Isolated Compounds

**Figure 6:**
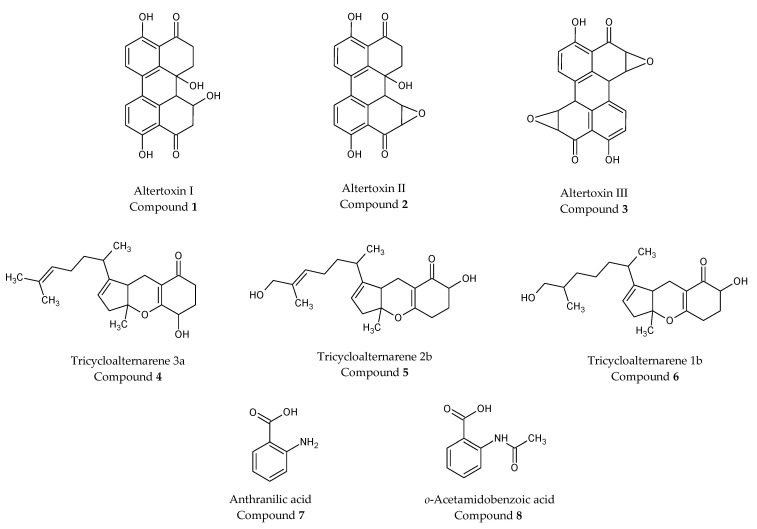
Pure compounds of the endophytic fungus *Alternaria alternata* isolated from *Calotropis procera*.

As extract A from *Alternaria alternata* constitutes the most interesting of the five fungal endophytic extracts based on its strong anti-inflammatory and cytotoxic activity (Figure S1), its ethyl acetate fraction was investigated for the isolation of different constituents. Eight compounds were isolated, and their structures were determined via NMR and mass fragmentation and then confirmed via comparison with the literature. Three perylenequinone metabolites, altertoxin I (compound **1**), altertoxin II (compound **2**) and altertoxin III (compound **3**); three tricycloalternarenes, tricycloalternarene 3a (compound **4**), tricycloalternarene 2b (compound **5**) and tricycloalternarene 1b (compound **6**); and two nitrogen compounds, anthranilic acid (compound **7**) and *o*-acetamidobenzoic acid (compound **8**) were identified. The chemical structures are presented in the Figure 6.

For the characterization of compound **1**, i.e., altertoxin I (Figure S2), the exploitation of the mass spectrum in ionization mode by electrospray showed an ionic peak appearing at *m*/*z* 353.0973 attributable to the radical cation [M + H]^+^ in agreement with the molecular formula C_20_H_17_O_6_ (Figure S3). MSMS spectra showed the fragments at 335.0868 [M-H_2_O + H]^+^ (calcd.335.0914); 317.0759 [M-2H_2_O + H]^+^ (calcd. 317.0808) 299.0615 [M-3H_2_O + H]^+^ (calcd. 299.0703); 289.0781 [M-2H_2_O-CO + H]^+^ (calcd. 289.0859) and 271.0699 [M-3H_2_O-CO + H]^+^ (calcd. 271.0753). MSMS spectra data matched Pubchem data.

The analysis of the ^13^C NMR spectrum revealed two carbonyl groups resonating at 207.0 and 204.8 ppm (Figure S5), respectively. ^1^H NMR spectrum analysis (Figure S4) showed four doublets in the aromatic proton resonance region appearing at *δ* = 8.03, 7.99, 7.08, and 6.99 ppm with a coupling constant of *J* = 8.8 Hz, attributable to the resonance of four chemically non-equivalent aromatic protons, suggesting that these were two tetra-substituted benzyl rings. This suggestion was also confirmed via the homonuclear correlation tasks (*δ* = 8.03–7.08 and 7.99–6.99) visible on the COSY spectrum (Figure S6). The correlations present on the HSQC spectrum (Figure S7) confirm the presence of three methylene groups by determining the chemical shifts of the methylene protons (δHα = 2.70 ppm) and (Hβ = 3.17 ppm) and of the secondary carbon (C = 34.8 ppm), (Hα = 2.48 ppm) and (Hβ = 3.17 ppm) and secondary carbon (C = 36.4 ppm) and (Hα = 2.97 ppm) and (Hβ = 3.01 ppm) and secondary carbon (C = 48.9 ppm).

In addition, the confirmation of the chaining of the hydrocarbon skeleton of altertoxin I was carried out via exploration of the HMBC spectrum (Figure S8), making it possible to visualize the heteronuclear correlation at long distances (^2^J and ^3^J) by observing the C-9/H-8α correlation tasks; C-9/H-8β; C-4/H-5α; C-4/H-5β and C-9b/H-6b.

For the characterization of compound **2**, i.e., altertoxin II (Figure S9), the analysis of the mass spectrum (Figure S10), which indicated the presence of an ionic peak at *m*/*z* 351.0844 attributable to the radical cation [M + H]^+^, was in agreement with the molecular formula C_20_H_15_O_6_. MSMS spectra showed the fragments at 333.0735 [M-H_2_O + H]^+^ (calcd.333.0758); 315.0607 [M-2H_2_O + H]^+^ (calcd. 315.0652); 305.0789 [M-H_2_O-CO + H]^+^ (calcd. 305.0808); 287.0646 [M-2H_2_O-CO + H]^+^ (calcd. 287.0803); 263.0658 [M-H^2^O-CO-O-ChCH + H]^+^ or [C_17_H_10_O_3_ + H]^+^ (calcd. 263.0703). Compound **2** was determined to be an analog of compound **1**, based on the comparison of their 1D NMR data and 2D NMR analyses (Figures S11–S15). The comparison of the two ^1^H spectra (Figures S4 and S11) allowed us to note the common presence of all the signals of the aromatic protons (H-1, H-2, H-11 and H-12) and methylene protons H-5α, H-5β, H-6αand H-6β. These spectral data pointed us towards a structure analogous to altertoxin I with a difference in the B nucleus (Figure S9). The appearance of two other doublets, integrating one proton (1H) each, with a low coupling constant (3.6 Hz) around 3.67 ppm and 4.33 ppm, which were carried by the carbons δC = 54.3 and δC = 57.2 ppm respectively according to HSQC spectra (Figure S14), showed the presence of an epoxide fragment between the C-7/C-8 carbons. The correlation spots visible on the HMBC spectrum (Figure S15) between the broad singlet around δH = 3.55 ppm with the C-6a, C-7, C-12a and C-12c carbons as well as the absence of multiplicity showed that the H-6b and H-7 protons were not on the same plane due to the effect of the three-membered ring of the epoxide.

For the characterization of compound **3**, i.e., altertoxin III (Figure S16), the analysis of the mass spectrum (Figure S17), indicating the presence of an ionic peak at *m*/*z* 349.0677 attributable to the radical cation [M + H]^+^, was in agreement with the molecular formula C_20_H_13_O_6_. MSMS spectra showed the fragments at 319.0549 [M-CH_2_O + H]^+^ (calcd. 319.0601); 303.0600 [M-HCOOH + H]^+^ (calcd. 303.0652); 275.0650 [M-HCOOH-CO + H]^+^ (calcd. 275.0703); 247.0705 [M-HCOOH-2CO + H]^+^ (calcd. 247.0753); 219.0760 [M-HCOOH-3CO + H]^+^ (calcd. 219.0804) and 191.0824 [M-HCOOH-4CO + H]^+^ (calcd. 191.0855). The isolated compound **3** was very unstable, as it darkened on the columns during purification and on the TLC plates and even in the NMR tube 30 min after sample preparation. The analysis of its ^1^H NMR spectrum (Figures S18 and S19) showed two doublets in the aromatic proton resonance region appearing at δH = 7.60 and 6.90 ppm, two doublets in the proton resonance region, bound directly to heteroatoms δH = 4.60 and 3.86 ppm, and a broad singlet around 4.22 ppm. These chemical shifts as well as the values of the calculated coupling constants were perfectly in agreement with the spectral data of altertoxin III, which possessed an element of symmetry as evidenced by its ^1^H NMR spectrum, which exhibits signals corresponding to half of the nuclei present as mentioned in literature [24]. Protons were bonded to carbon via HSQC spectrum analysis (Figure S20).

For the characterization of compound **4**, i.e., tricycloalternarene 3a (Figure S21), the analysis of the mass spectrum (Figure S22), indicating the presence of an ionic peak at *m*/*z* 331.2303 attributable to the radical cation [M + H]^+^, was in agreement with the molecular formula C_21_H_31_O_3_. MSMS spectra showed the fragments at 313.2152 [M-H_2_O + H]^+^ (calcd. 313.2162); 295.2045 [M-2H_2_O + H]^+^ (calcd. 295.2056); 277.1907 [M-3H_2_O + H]^+^ (calcd. 277.1951), and then the loss of CHx matched the carbonated structure. The structure was confirmed via 1D NMR data and the 2D NMR analyses (Figures S23–S28). Analysis of the ^1^H NMR spectrum (Figure S23) showed the presence of a side chain derived from an isoprene unit (2-methylbuta-1,3-diene). Indeed, the ^1^H NMR spectrum allowed for the observation of two integrating singlets for three protons (3H) each, appearing at δH = 1.65 ppm and δH = 1.55 ppm; an integrating doublet for three protons (3H), appearing at δH = 0.93 ppm with a coupling constant *J* = 6.8 Hz; an integrating multiplet for a proton (1H), appearing at δH = 5.02 ppm; an integrating multiplet for two protons (2H), appearing at δH = 1.84 ppm; two integrating multiplets for one proton (1H) each, appearing at δH = 1.45 ppm and δH = 1.28 ppm; and an integrating multiplet for a proton (1H), appearing at δH = 1.96 ppm. On the ^1^H-^1^H COSY spectrum (Figure S25), scalar correlation between δH = 5.02 ppm/δH = 1.84 ppm and δH = 1.96 ppm/δH = 0.93 ppm allowed us to confirm the structure of the compound. The exploration of the HSQC spectrum (Figure S26) also allowed us to confirm the presence of the ethylenic proton H3 by means of direct proton–carbon correlation tasks. In fact, the proton appearing at δH = 5.02 ppm, correlating with the resonant tertiary carbon at δC = 124.6 ppm, was in agreement with our conclusion. The ^1^H NMR spectrum (Figure S23) still showed a broad integrating singlet for a proton (1H) at δH = 5.31 ppm and an integrating triplet for a proton (1H) at δH = 4.33 ppm with a coupling rate of *J* = 5.6 Hz. These protons were carried by the carbons δC = 119.8 ppm (*sp*^2^) and δC = 66.6 ppm (oxygenated carbon), respectively (HSQC spectrum, Figure S26). The ^13^C NMR spectrum (Figure S24) showed the presence of two unshielded quaternary carbons, one at δC = 196.8 ppm and the other at δC = 169.9 ppm. These chemical shifts were characteristic of the α,β-unsaturated carbonyl function characteristic of tricycloalternarene derivatives. In order to confirm the sequence of the carbon skeleton within this fragment, we used the total spectrum of HMBC (Figure S27), starting from the chemical shift of the carbonyl C-18. The following correlation are visible: H-12α/C-18, H-12β/C-18, H-15/C-14 and H-10′/C-10. The position of the isoprenoid chain was highlighted by the observation of correlation H-6/C-7, visible on the HMBC spectrum. After comparing the chemical shifts and multiplicities of tricycloalternarenes with the literature [25], we confirmed the chemical structure of tricycloalternarene 3a.

For the characterization of compound **5**, i.e., tricycloalternarene 2b (Figure S29), the mass spectrum analysis (Figure S30), indicating the presence of an ionic peak at *m*/*z* 347.2226 attributable to the radical cation [M + H]^+^, was in agreement with the molecular formula C_12_H_31_O_4_. MSMS spectra showed the fragments at 329.2245 [M-H_2_O + H]^+^ (calcd. 329.2111); 311.2048 [M-2H_2_O + H]^+^ (calcd. 311.2006); 293.1839 [M-3H_2_O + H]^+^ (calcd. 293.1900) and then the loss of CHx matches with carbonated structure. MSMS spectra matched spectra suggested by Pubchem. Compound **5** was determined to be an analog of compound **4**, based on the comparison of their 1D NMR data and the 2D NMR analyses (Figures S31–S34).

On the ^1^H NMR spectrum (Figure S31), we can identify all the signals of the protons of the basic skeleton of the cycles A, B and C with the exception of the protons H-15, H-16 and H-17. We observed a deshielded methine in the form of a split doublet integrating for a proton (1H) appearing at δH = 4.02 ppm, with coupling constants of *J* = 12.8 Hz and *J* = 5.2 Hz linked to a carbon δC = 71.1 ppm (HSQC, Figure S33), which led us to think that the hydroxyl group (-OH) would be in position 17 and is unshielded with respect to position 15 due to the attracting effect of the carbonyl group (formation of hydrogen bonds). In the ^1^H spectrum of compound **5,** we observed the disappearance of a singlet integrating for three protons (3H), relative to the proton of one of the two methyl groups of the isoprenoid derivative and the appearance of a singlet around 3.95 ppm integrating for two protons (2H). These observations directed us towards a tricycloalternarene with a structure similar to that of tricycloalternarene 3a and possessing an oxygenated methylene group in position 2 of the side chain. The absence of methoxy protons in the proton deshielding region (3.4–3.9 ppm) led us to believe that the oxygen radical was a simple hydroxyl. The analysis of the HSQC spectrum (Figure S33) confirmed the presence of seven methylene groups; five methine groups, two of which are *sp*^2^ hybridized carbons; one oxygenated group; and three methyl groups. This description suggests that the structure is that of tricycloalternarene 2b [26].

For the characterization of compound **6**, i.e., tricycloalternarene 1b (Figure S35), the analysis of the mass spectrum (Figure S36), indicating the presence of an ionic peak at *m*/*z* 349,2472 attributable to the radical cation [M + H]^+^, was in agreement with the molecular formula C_21_H_33_O_4_. MSMS spectra showed fragments at 285.2172 [M-2H_2_O-CO + H]^+^ (cacld. 282.2212) and then the loss of CHx matched carbonated spectra. Compound **6** was determined via a study of 1D NMR data and the 2D NMR analyses (Figures S37–S40). Compound **6** is an analog to compounds **4** and **5**.

For the characterization of compound **7**, i.e., anthranilic acid (Figure S41), the analysis of the mass spectrum (Figure S42), indicating the presence of an ionic peak at *m*/*z* 136.0353 attributable to the radical cation [M − H]^−^, was in agreement with the molecular formula C_7_H_6_NO_2_. MS spectra showed an adduct with 2 or 3 compounds and an adduct with sodium (Na) or potassium (K), such as 448.0840 for [3(C_7_H_7_NO_2_) + K-2H]^−^ (cacld. 448.0916). MSMS spectra showed a fragment at 92.0509 [M-CO_2_-H]^−^ (cacld. 92.0506). MSMS spectra matched spectra suggested by Pubchem.

Compound **7** was determined via study of 1D NMR data and the 2D NMR analyses (Figures S43–S46).

For the characterization of compound **8**, i.e., *o*-acetamidobenzoic acid (Figure S47), MSMS spectra (Figure S48) showed a fragment at 136.0379 [M-CH_2_CO-H]^−^ (cacld. 136.0404); 134.0610 [M-CO_2_-H]^−^ (cacld. 134.0611) and 92.0539 [M-CH_2_CO-CO_2_-H]^−^ (cacld. 92.0506). The study of 1D NMR and the 2D NMR analyses were carried out to confirm this structure (Figures S49–S52).

## 3. Discussion

In this work, general screening of anti-inflammatory activity of five endophytic fungi ethyl acetate crude extracts was performed. Indeed, as some biological extracts endowed with antioxidant activity are also endowed with anti-inflammatory activity [27], we first investigated if these endophytic fungi extracts were able to counteract the LPS pro-inflammatory effect on THP-1 cells differentiated into macrophages. This cell type was chosen for this study because macrophages not only constitute a first line of defense against pathogens but are key players in tissue homeostasis and inflammation resolution, among other conditions, due to their ability to produce different types of cytokines. Indeed, during inflammation, auto-immunity, infection and/or cancer, they play an active role and tissue-resident macrophages can also play a resolving role or exacerbate the disease [28,29]. Thus, find biomolecules able to counteract excessive and/or uncontrolled inflammation is crucial and, in this context, find biomolecules able to reduce pro-inflammatory cytokine production such as TNF-α constitutes a prime target.

Our results showed that extract A of *Alternaria alternata* from *Calotropis procera* leaves and extract C from *Aspergillus terreus* from *Trigonella foenum-graecum* seeds diminished TNF-α production of THP-1 cells of 28% (at 20 µg/mL) and 54% (60 µg/mL), respectively, one hour after LPS. Only these two extracts were able to inhibit the pro-inflammatory effect of LPS. Moreover, they were able to induce an anti-inflammatory state rendering of THP-1 cells less sensible to the LPS pro-inflammatory stimulus. We have shown previously that extract C had also antioxidant activity (IC_50_ 18.01 ± 0.1 µg/mL) [22].

In addition, we have performed a metabolomics study with a view to describing the chemical composition of these extracts, which has never been studied before. We performed phytochemical analysis and metabolomics to obtain a general chemical profile of all endophytic extracts in view to identify compounds and to improve knowledge of chemistry of these fungal endophytes extracts.

HPTLC analysis showed significant differences in the presence of specialized metabolites in the different endophytic fungi. All extracts contained terpenoids. These compounds were frequently found in fungal endophytes. A group of Brazilian researchers identified 127 terpene compounds from fungal endophytes studied between 2006 and 2010, more than half of which were sesquiterpenes [30]. In general, terpenes play various roles in mediating interactions between organisms, such as plants/fungi, whether antagonistic or beneficial. A number of terpenes are toxins, repellents or attractants for other organisms, which led to the postulation that they have an ecological role in antagonistic or mutualistic interactions between organisms [31]. The HPTLC profile of extract A was completely different from the others. It showed high level of metabolites such as terpenoids, polyphenols and alkaloids. HPTLC profiles of the strains B, C and D were similar but not identical, although all were obtained from *Aspergillus terreus*. This can be explained by the fact that these three extracts came from the same endophyte fungus (*Aspergillus terreus*) which were isolated from two different plant species (*Calotropis procera* leaves and *Trigonella foenum-graecum* seeds).

The extracts A, B, C and D contained alkaloids. Alkaloids are toxic specialized metabolites in a large number of species of phytophagous insects and herbivorous mammals. Like terpenoids, alkaloids are compounds frequently observed in fungal endophytes [30].

GC-MS analysis revealed the variety of the chemical composition of the five fungal endophytic extracts. Fatty acids were present in all extracts, and linoleic acid was a ubiquitous compound. Itaconic acid is a molecule often identified in strains of the Aspergillus genus. This compound has industrial interests, in particular for the production of synthetic resins, plastics and rubbers. Derivatization of the extracts also confirmed the presence of compounds previously detected in non-derivatized extracts. This was particularly the case for fatty acids and for lovastatin in extract C. Ergosterol, the primary sterol in the cell membranes of filamentous fungi, has been detected via GC-MS only in extract E, that of *Cladosporium cladoporioides*. In the literature, this metabolite has been identified in various species of fungus such as *Aspergillus*, *Penicillium*, *Fusarium*, *Rhizopus*, *Cladosporium* and *Alternaria* [32]. Ergosterol concentration has been widely used as an estimate of fungal biomass in various environments.

To confirm the identification of molecules or to reveal other molecules produced by the five strains of endophytic fungi, molecular networking workflow was performed. Every node represented one chemical entity, while clusters of nodes corresponded to structurally related molecules based on similarity between their MS^2^ spectrum patterns [33]. Clusters, including nodes noted as compounds previously characterized, are called molecular families [34]. Complementary strategies created with the built-in automatic library search by GNPS followed by MolNetEnhancer Workflow, manual confirmations based on MS^2^ fragmentation pattern and NMR data for isolated metabolites were used to confirm the molecules described.

The three strains of endophytic fungi *Aspergillus terreus* showed the ability to produce the polyketide compound lovastatin. The fungus *Aspergillus terreus* has dominated the biological production of drugs known as statins [35]. Statins, initially discovered in fungi, are a class of drugs that inhibit HMG-CoA reductase and lead to decreased cholesterol production [36]. These compounds were identified via by GC-MS and by LC-MS in the networks 1B, 1C and 1D corresponding to lactones/delta valerolactones.

*Aspergillus terreus*, from which extract C is derived, is well known for industrial and pharmaceutical applications, whereas *Alternaria alternata*, from which extract A comes*,* is less well known. For this reason, we also undertook the metabolite purification of extract A.

The genus Alternaria is a cosmopolitan fungal genus widely distributed in soil and organic matter. It includes saprophytic, endophytic and pathogenic species belonging to the very large Pleosporaceae family. It contains about 300 species of so-called black molds, which are considered both saprophytes and major pathogens (e.g., *A. alternata*, *A. tenuissima*, *A. solani* and *A. infectoria*), which cause disease in many plants and can infest a wide variety of agricultural crops such as cereals, tomatoes, sunflower seeds, citrus fruits, apples, grapes and olives. The consequences are often considerable economic losses due to the deterioration of crops or an alteration to the visual appearance of agricultural products [37,38,39]. *A. alternata* is the most frequently reported species, infecting nearly 100 plant species. It is also responsible for post-harvest diseases in various crops, causing asthma and upper respiratory tract infections in humans and livestock because specialized metabolites are catabolized into toxic metabolites in the bodies of living organisms [40,41]. The reasons for the pathogenicity of this fungus are the production of various mycotoxins (the toxins produced by fungal molds), which are toxic to humans and animals, so they are closely associated with its phytopathogenicity [41,42]. Species of the genus Alternaria have been widely studied [39]. Around 300 metabolites have been reported in the past few decades [43]. They are mainly derivatives of pyranones, quinones, terpenoids, phenolic compounds and nitrogenous metabolites (amide, cyclopeptides), some of which exhibit phytotoxic, cytotoxic, antifungal and antimicrobial activities [39,41,44]. Here, the isolation of compounds from extract A allowed us to identify three perylenequinone metabolites as altertoxin I (compound **1**) [24], altertoxin II (compound **2**) [24] and altertoxin III (compound **3**) [24]; three tricycloalternarenes as tricycloalternarene 3a (compound **4**) [25], tricycloalternarene 2b (compound **5**) [26] and tricycloalternarene 1b (compound **6**) [25]; anthranilic acid (compound **7**), an aminobenzoic acid that is benzoic acid having a single amino substituent located at position 2; and *o*-acetamidobenzoic acid (compound **8**). This compound is an amidobenzoic acid consisting of benzoic acid bearing an acetamido substituent at the ortho position. The compounds 7 and 8 were described for the first time in *Alternaria alternata* endophytic fungus, and the chemical structures are presented in Figure 6.

Most mycotoxins exhibit toxic effects, even at low concentrations, in humans and animals [39]. Among the various secondary metabolites produced by fungi of the genus Alternaria, alternariol, alternariol 9-methyl ether and other lactones resorcylic acids or resorcylic acids are the main toxic metabolites described [42,45,46]. Although their toxicity is low compared to other mycotoxins (e.g., aflatoxins or ochratoxins), Alternaria species cause significant harvest losses of fruits, vegetables, juices and other products and can lead to allergic reactions, skin infections, keratitis and otitis in humans. For example, *A. alternata* is known to be a potent allergen [47,48,49]. In this work, Alternaria extract A showed a high anti-inflammatory activity and chemical diversity. The pure compounds can be tested and used in pharmaceutical applications.

Besides their toxic effects, mycotoxins have also attracted the attention of scientists for the wide range of their beneficial biological activities. Indeed, they exhibit anti-inflammatory, anti-tumoral, antimicrobial and phytotoxic activities. For example, porritoxin, isolated from *A. porri* has been investigated as a potential chemopreventive agent for cancer; depudecine, a histone deacetylase (HDAC) inhibitor, isolated from *A. brassicicola*, has also shown antitumor potency. Altertoxin I completely inhibited HIV-1 virus replication at concentrations of 2.20 μM [42]. In the agricultural field, tenuazonic acid, maculosin and tentoxin have been studied as herbicide candidates [49,50,51].

Altertoxins are perylenequinones, and under illumination, these metabolites react with molecular oxygen to generate reactive oxygen species (ROS), which in excess can damage cellular macromolecules and trigger apoptosis [52]. For example, above certain concentrations, altertoxins I, II and III were shown to be mutagenic [24], while altertoxin I inhibited pyruvate dehydrogenase phosphorylation [53].

Altertoxin II (compound **2**) is particularly unique due to the presence of an epoxide group which is responsible for its various biological activities, including anti-inflammatory properties. It should be noted that altertoxin II was evaluated as the most active mycotoxin in terms of anti-inflammatory activity [54,55]. Indeed, in the presence of mitochondrial superoxide ions inducing the production of membrane cholesterol, this compound was the most effective at promoting mitochondria reorganization and transcription factor NF-κB activity decrease in THP-1 macrophages, also indicating a reduction of inflammatory activity. These data also showed that altertoxin II, well known to exert a total suppression of NF-κB activation in THP-1 macrophages at generally subtoxic concentrations, can also act as an anti-inflammatory agent [54,55]. Interestingly, these data are in correlation with what we observed with extract A: at low concentrations, anti-inflammatory activity was observed and if concentrations increased, cytotoxicity appeared on THP-1 cells. Nevertheless, experiments with pure compounds of extract A, especially altertoxins, have to be performed to test the hypothesis that in extract A, altertoxin II will be responsible for the anti-inflammatory effect observed.

Among the other many molecules produced by *Alternaria alternata*, tricycloalternarenes have been previously described [25,56]. These metabolites represent a group of fugal-derived meroterpenoids less well known as altertoxins. These compounds are of interest to natural drug chemists/biologists due to their remarkable antimicrobial and cytotoxic effects. For example, the tricycloalternarene 3a is a significant tyrosine kinase inhibitor [57], while tricycloalternarene 2b exhibits potent in vitro cytotoxicity against the human lung adenocarcinoma A549 cell line [58]. As these molecules are present in extract A, it will be of interest to perform experiments on THP-1 cells and other cell types to determine if they are endowed with an anti-inflammatory activity.

## 4. Materials and Methods

### 4.1. Fungal Endophyte Strains

The fungal endophytes strains studied in this project were five (5) strains isolated and characterized in the article by Khiralla et al. [59]: *Alternaria alternata*, strain A, isolated from the leaves of *Calotropis procera*; *Aspergillus terreus,* strain B, isolated from the stems of *Calotropis procera*; *Aspergillus terreus*, strain C, isolated from the seeds of *Trigonella foenum-graecum* in 2014; *Aspergillus terreus*, strain D, isolated from the seeds of *Trigonella foenum-graecum* in 2015; and *Cladosporium cladosporioides,* strain E, isolated from the leaves of *Vernonia amygdalina*.

### 4.2. Culture of Endophytic Fungi

The fungal endophytes (strains A, B, C, D and E, Figure 1) were cultured in a medium composed of potato extract (4 g/L), dextrose (20 g/L) and agar-agar (15 g/L) also called PDA (potatoes–dextrose–agar). The media were prepared with 39 g/L of PDA (Sigma Aldrich, St. Louis, MI, USA) and pure water. The whole was autoclaved for 20 min at 250 °C. Approximately 20 mL of PDA medium was poured into each Petri dish (diameter = 9 mm) in a sterile field under a vertical flow fume cupboard. The endophyte strains were cultured on a PDA medium described previously. In a sterile field, the fungal endophyte was inoculated in the center of the agar nutrient medium, and the whole was incubated at 28 °C. The incubation period varied from 9 to 30 days, depending on the growth rate of the fungal strains.

### 4.3. Preparation of Fungal Endophytic Extracts

Five extracts were obtained from fresh strains of fungal endophytes. The contents of twenty boxes of endophytes and their medium were ground with approximately 200 mL of ethyl acetate in a blender. The mixture obtained was transferred to a 1 L Erlenmeyer flask and supplemented with ethyl acetate up to 1 L. The whole macerated for 24 h, and manual stirring was carried out several times (3 to 7 times). The macerate obtained was filtered using filter paper and a piece of cotton. The solvent was then evaporated using a rotary evaporator. The extracts were transferred into hemolysis tubes for storage at +4 °C. The extracts obtained were used for biological tests (cytotoxicity and anti-inflammatory) and phytochemical analysis (NMR, GC-MS and LC-MS).

### 4.4. Cytotoxicity and Anti-Inflammatory Activity

The THP-1 human monocytic leukemia cell line was purchased from the European Collection of Authenticated Cell Cultures (ECACC). THP-1 cells were used for cytotoxic and anti-inflammatory assays. A similar protocol is described in our previous work [60].

#### 4.4.1. Cell Culture and Treatments of THP-1 Cells

THP-1 cells were cultured in RPMI 1640 medium supplemented with 10% heat-inactivated fetal calf serum, 100 U/mL penicillin, 100 µg/mL streptomycin, 10 mM HEPES, 2 mM L-glutamine, 1 mM sodium pyruvate and 1 × non-essential amino-acids at 37 °C under 5% CO_2_. All products were purchased from SIGMA-Aldrich (Saint-Quentin-Fallavier, France). THP-1 cells, at a density of 0.8 × 10^6^ cells/mL in 24-well plates (Dutscher, Bernolsheim, France), were differentiated for 3 days into macrophages by adding to the cell culture medium 20 nM of phorbol myristate acetate (SIGMA-Aldrich). Then, differentiated THP-1 cells were incubated for 24 h with 100 ng/mL of LPS from *Escherichia coli* (serotype 0111:B4; SIGMA-Aldrich) added one hour before or after an endophytic fungal extract. Stock solutions of these extracts were prepared at 5 mg/mL by dissolving them with 70% ethanol solution and sterilized with 0.2 µM syringe filters (SIGMA-Aldrich). Thus, the five endophytic fungal extracts were tested at 5, 10, 20, 40, 60 and 100 µg/mL, and ethanol solvent was used as control.

#### 4.4.2. THP-1 Cell Viability Measurement

After 24 h of incubation with LPS and/or endophytic fungal extract, differentiated THP-1 cell viability was determined using a crystal violet assay. THP-1 cells were washed twice with phosphate buffer saline (PBS), incubated with 0.1% crystal violet (SIGMA-Aldrich) for 20 min at ambient temperature and then carefully washed twice with PBS. Finally, cells were lyzed with 10% acetic acid for 20 min at ambient temperature. Cell contents were homogenized and analyzed via spectrophotometry at 595 nm with a multilabel counter (Wallac-1420, Perkin Elmer, Boston, MA, USA). Each experiment was performed in triplicate.

#### 4.4.3. TNF-α Quantification from THP-1 Cell Culture Supernatants

After 24 h of treatment with LPS and/or endophytic fungal extracts, THP-1 cell culture supernatants were harvested in sterile conditions, centrifuged to remove dead cells and stored at −80 °C until ELISA tests. TNF-α concentrations were determined using the Human TNF-alpha Quantikine ELISA Kit (R&D Systems, BioTechne Brands, Rennes, France). Assays were performed according to the instructions of the manufacturer, in duplicate and repeated three to four times. Plates were read at 450 nm with a multilabel counter (Wallac-1420, Perkin Elmer, Boston, MA, USA).

### 4.5. Data Analysis

For THP-1 cell viability and anti-inflammation studies, six independent experiments were performed for each fungal endophytic extract. *T*-tests were used to identify statistically significant differences (*p* ≤ 0.05) using GraphPad Prism 9.

### 4.6. High Performance Thin Layer Chromatography (HPTLC): Equipment and Method

Chromatographic analysis was carried out with silica gel 60 F254 plates (Merck) fixed on an aluminum coating. The mobile phase chosen consisted of cyclohexane and ethyl acetate at a proportion of 7:3 (*v*/*v*). The observation of the spots was made in visible light or under an ultra-violet (UV) lamp at 254 nm or 365 nm. Four chemical derivative reagents were used: sulfuric reagent (10% of H_2_SO_4_), Neu’s reagent, Dragendorff and sulfuric anysaldehyde.

TLCs were performed with extracts of fungal endophytes solubilized at 20 mg/mL of ethyl acetate. The deposition of samples is carried out on the TLC plates using a semi-automatic sample application system “Linomat 5” (CAMAG, Muttenz, Switzerland). The filling of the syringe (capacity of 100 μL), its rinsing (with acetone) and its insertion were completed manually. Samples of 10 μL were sprayed onto a 6 mm strip with nitrogen (1.0 L/min) at a pressure of 4–6 bar.

### 4.7. Gas Chromatography Coupled to Mass Spectrometer (GC-MS): Equipment and Method

The GC-MS analyzes were carried out using a QP2010-Shimadzu equipment equipped with a quadrupole mass detector and operating in electron ionization mode at 70 eV. An apolar “DB-5 MS Agilent” column (30 m × 0.25 mm × 0.25 μm) was used with a temperature program of 36 min at 60–320 °C at 5 °C/min. The temperature of the injector was 250 °C; the flow rate of carrier gas (helium) was 1 mL.min^−1^. The injection was made in split mode with a division ratio of 1/5. The quantity of extracts injected was 1 μL. The identification of the compounds was carried out by comparing the measured data with those of the NIST library (V2.0-19/05/2011). The extracts were dissolved in dichloromethane at a concentration of 20 mg/mL.

The derivatization was carried out by silylation of the extracts by modifying the method of A.K Kiprop (2013) [61]. Thus, 10 mg of extracts were dissolved in 200 μL BSTFA-TMCS (99:1 *v*/*v*). The whole was heated in an oil bath at 54 °C for 20 min with magnetic stirring. The solutions were diluted 1/10 with dichloromethane for their injection into GC-MS under the same conditions as above.

### 4.8. Liquid Chromatography Coupled to Mass Spectrometer (LC-MS): Equipment, Method and Molecular Networking

The LC system consisted of a U3000-Dionex apparatus with an injector comprising a 1 µL loop. The LC analytical column used was a Hypersil Gold (100 mm × 2.1 mm, Thermo Scientific, Bellefonte, PA, USA) and eluted at a flow rate of 200 µL/min using a gradient 0 mn 5%B/5 mn 5%B/40 mn 99%B/45 mn 99%B/50 mn 5%B/55 mn 5%B. Solvent A consisted of water/2% of formic acid (HCOOH), and solvent B consisted of acetonitrile (ACN). The oven temperature was set at 40.00 °C, and 2 µL was injected. The LC-MS analysis was performed using a micrOTOF_Q_^TM^ apparatus (Bruker Daltonics, Bruker, Bremen, Germany), and the MS/MS data are obtained using Electrospray Ionization—High Resolution Mass Spectrometry (ESIHRMS). A mass range of 50–1000 *m*/*z* and collision energy of 20 eV was used. The raw data were converted using Bruker DataAnalysis; each data point was calibrated with sodium formate. All MS/MS data were converted in a mascot generic file (.mgf) file.

The analyses were performed in mass spectrometry of the L2CM laboratory at the University of Lorraine, France.The .mgf file was sent to the GNPS website [33]. A molecular network was created using the online workflow on the GNPS platform (http://gnps.ucsd.edu (accessed on 26 June 2023)).

The data were filtered by removing all MS/MS fragment ions within ±17 Da of the precursor m/z. MS/MS spectra were window-filtered by choosing only the top 6 fragment ions in the ±50 Da window throughout the spectrum. The precursor ion mass tolerance was set to 2.0 Da and an MS/MS fragment ion tolerance of 0.05 Da. A network was then created where edges were filtered to have a cosine score above 0.7 and more than two matched peaks.

Further, edges between two nodes were kept in the network if and only if each of the nodes appeared in each other’s respective top 10 most similar nodes. Finally, the maximum size of a molecular family was set to 100, and the lowest-scoring edges were removed from molecular families until the molecular family size was below this threshold. The spectra in the network were then searched against GNPS spectral libraries. The library spectra were filtered in the same manner as the input data. All matches were kept between network spectra, and library spectra were required to have a score above 0.7 and at least three matched peaks.

To enhance chemical structural information within the molecular network, information from in silico structure annotations from GNPS Library Search were incorporated into the network using the GNPS MolNetEnhancer workflow (https://ccms-ucsd.github.io/GNPSDocumentation/molnetenhancer/, (accessed on 26 June 2023)) on the GNPS website (http://gnps.ucsd.edu (accessed on 26 June 2023)). Chemical class annotations were performed using the ClassyFire chemical ontology [33,62,63].

For the visualization of molecular networking, the software Cytoscape^®^ (version 3.8.2) was used [23]. A similar protocol was described in the article Elmi et al. [64]

### 4.9. Isolation of Pure Compounds from Extract A, from Alternaria alternata Endophyte

#### 4.9.1. General Experimental Procedures

Column chromatography (CC) was carried out on an open silica gel column or on Sephadex gel filtration (Sephadex^TM^ LH-20, GE Healthcare Bio-Sciences AB, Uppsala, Sweden).

High-performance thin layer chromatography (HPTLC) was performed on pre-coated silica gel plates (silica gel plates 60 F254 Merck on aluminum bracket) using two mobile phases: dichloromethane:methanol (DCM:MeOH, 95:5) or hexane:EtOAc (7:3). Spot were located by visualization under UV at 254 nm and 365 nm.

An analytical control of each fraction was carried out on a GILSON High Performance Liquid Chromatography (HPLC) chain equipped with a UV/Visible detector with strips of diode (DAD) (PIMS-DET-UV-04). The analytical column used was a reverse phase GOLD 250-046 with a binary solvent system methanol: 2% formic acid/water: 2% formic acid. The flow rate was 1 mL/min. For analytical controls, an appropriate elution gradient was used for each sample. The compounds were then isolated on a GILSON semi-preparative HPLC line with an LTD collector injector, connected to a UV detector (DAD) (PIMS-DET-UV-04). The column used was a semi-preparative column THERMO GOLD column, 250 mm × 10 mm, with a methanol: 2% formic acid/water: 2% formic acid binary solvent system in gradient mode and a flow rate of 1 mL/min.

MS/MS spectra were recorded via Electrospray Ionization—High Resolution Mass Spectrometry (ESIHRMS). A mass range of 50–1000 *m*/*z* and collision energy of 20 eV was used.

All ^1^H and ^13^C-NMR spectra were recorded on a Bruker Avance III 400 spectrometer (Bruker BioSpin, Rheinstetten, Germany), operating at a frequency of 400.13 MHz at a temperature of 26 °C using a BBFO Probe and a Bruker sample changer. NMR analyses were performed at the CPM NMR facility of Université de Lorraine. One-dimensional proton and carbon nuclear magnetic resonance spectra (^1^H and ^13^C NMR) were recorded at 400 MHz and 100 MHz, respectively, in deuterated methanol (CD_3_OD) or in deuterated chloroform (CDCl_3_) or in deuterated dimethyl sulfoxide (DMSO-*d*_6_). One-dimensional and two-dimensional homonuclear (COSY) and heteronuclear (HSQC, HMBC) NMR experiments were performed using a Bruker Avance III 400 spectrometer (Bruker BioSpin, Rheinstetten, Germany), operating at a frequency of 400.13 MHz at a temperature of 26 °C using a BBFO probe and a Bruker autosampler. The spectra thus obtained were reprocessed with the MestreNova software (version 14.3.1) and Bruker TopSpin software (version 4.0.6). The chemical shifts (δ) are expressed in parts per million (ppm) and the coupling constants (*J*) in Hertz (Hz).

#### 4.9.2. Isolation of Pure Compounds from Strain A

The ethyl acetate (EtOAc) extract (550 mg) was subjected to Sephadex™ LH-20 gel filtration (VWR) eluted with dichloromethane:methanol (DCM:MeOH, 2:8, 500 mL). A total number of 11 fractions (20 mL each) was collected. Fraction 5 (40 mg) was subjected to semi-preparative HPLC using a gradient of MeOH to water (H_2_O) as eluent to obtain compound 1 (3.7 mg) and compound 2 (5.2 mg). For the next column, 14 g of the EtOAc extract were subjected to open silica gel column chromatography eluted using a gradient of hexane:EtOAc (9:1, 500 mL; 8:2, 500 mL; 7:3, 500 mL; 6:4, 500 mL; 5:5, 500 mL; 3:7, 500 mL; 0:1, 500 mL) and then EtOAc:MeOH (1:1, 500 mL). A total number of 195 fractions (20 mL each) were collected and finally 13 fractions were obtained on combining the eluates according to their similarity behavior on TLC. Fraction F7 (1.14 g) was subjected successively to Sephadex gel filtration eluted with DCM:MeOH (1:1, 500 mL) and subjected to semi-preparative HPLC using a MeOH:H_2_O mixture of increasing polarity as eluent to obtain pure compound 7 (8 mg). Fraction F8 (550 mg) was applied repeatedly to silica gel chromatography column (CC) and eluted using a gradient of DCM:MeOH (100:0, 500 mL to 80:20, 500 mL. Two sub-fractions were obtained. The sub-fraction F8-1 and F8-2 were then subjected to Sephadex gel filtration eluted with DCM:MeOH (1:1, 500 mL). Compound 4 (30 mg) was isolated after the injection of this last sub-fraction F8-1 to semi-preparative HPLC using a gradient of MeOH to water as eluent. Sub-fraction F8-2 was then subjected successively to open silica gel CC eluted using a gradient of DCM:MeOH (100:0, 500 mL to 60:0, 500 mL), to Sephadex gel filtration eluted with MeOH and finally to semi-preparative HPLC using a gradient of MeOH to water as eluent to afford pure compound 8 (2.8 mg). Fraction F9 (1.49 g) was applied repeatedly to silica gel CC and eluted with DCM:MeOH gradients to afford pure compound 3 (8 mg) in sub-fraction F9-3 (824 mg). This last one was then subjected to Sephadex gel filtration eluted with DCM:MeOH (2:8) and subjected repeatedly to silica gel CC and eluted using a gradient of DCM:MeOH and finally subjected to semi-preparative HPLC using MeOH:H_2_O mixture of increasing polarity as eluent to obtain pure compound 1 (60 mg), compound 5 (7.7 mg) and compound 6 (2.8 mg).

Compound **1**, Altertoxin I, was obtained as a yellow crystal. ^1^H NMR (400 MHz, CD_3_OD): δ (ppm) 8.03 (1H, d, *J* = 8.8 Hz, H-1)*,* 7.99 (1H, d, *J* = 8.8 Hz, H-12), 7.08 (1H, d, *J* = 8.8 Hz, H-2), 6.99 (1H, d, *J* = 8.8 Hz, H-11), 4.65 (1H, ddd, *J* = 11.2; 8.8; 5.2 Hz, H-7), 3.17, (1H, m, H-5β), 3.17, (1H, m, H-6β), 3.07 (1H, d, *J* = 8.8 Hz, H-6b), 3.01 (1H, dd, *J* = 16.0; 10.8 Hz, H-8β), 2.97 (1H, dd, *J* = 16.0; 5.2 Hz, H-8α), 2.70 (1H, dt, *J* = 18.4; 4.0 Hz, H-5α), 2.48 (1H, td, *J* = 15.6; 4.0, H-6α). ^13^C NMR (100 MHz, CD_3_OD): δ (ppm) 207 (C-4), 204.8 (C-9), 163.1(C-3), 162.6 (C-10), 141.3 (C-9b), 138.7 (C-12c), 133.8(C-1), 133.7 (C-12), 126.5 (C-12a), 125.3 (C-12b), 119.5 (C-2), 117.9 (C-9a), 117.2 (C-11), 115. (C-3a), 70.0 (C-6a), 66.7 (C-7), 53.3 (C-6b), 48.9 (C-8), 36.4 (C-6), 34.8 (C-5). HRESIMS (positive mode) calcd. for C_20_H_17_O_6_ [M + H]^+^ *m*/*z* 353.1025, found: 353.0973.

Compound **2**, altertoxin II, was obtained as a yellow crystal. ^1^H NMR (400 MHz, CD_3_OD): δ (ppm) 8.07 (1H, d, *J* = 9.2 Hz, H-1), 7.98 (1H, d, *J* = 9.2 Hz, H-12), 7.04 (1H, d, *J* = 8.8 Hz, H-2), 6.98 (1H, dd, *J* = 8.8; 1.2 Hz, H-11), 4.33 (1H, d, *J* = 3.6 Hz, H-7), 3.67 (1H, dd, *J* = 3.6; 0.8 Hz, H-8α), 3.55 (1H, sl, H-6b), 3.19 (1H, ddd, *J* =18.8; 14.0; 4.8 Hz, H-5β), 2.86 (1H, ddd, *J* =13.6; 5.2; 2.8 Hz, H-6β), 2.77 (1H, ddd, *J* = 18.0; 4.0; 2.8 Hz, H-5α), 2.47 (1H, ddd, *J* = 17.6; 13.6; 4.0 Hz, H-6α). ^13^C NMR (100 MHz, CD_3_OD): δ (ppm) 206.4 (C-4), 199.0 (C-9), 164.0 (C-3), 163.5 (C-10), 141.2 (C-9b), 136.9 (C-1), 134.1 (C-12), 136.9 (C-12c), 126.2 (C-12a), 124.8 (C-12b), 119.8 (C-2), 117.7 (C-11), 115.4 (C-9a), 115.0 (C-3a), 69.1 (C-6a), 57.2 (C-7), 54.3 (C-8), 46.3 (C-6b), 34.2 (C-5), 33.8 (C-6). HRESIMS (positive mode) calcd. for C_20_H_15_O_6_ [M + H]^+^ *m*/*z* 351.0869, found: 351.0844.

Compound **3**, altertoxin III, was obtained as a powder. ^1^H NMR (400 MHz, CD_3_OD): δ (ppm) 11.49 (4-OH), 11.49 (10-OH), 7.60 (1H, d, *J* = 8.8 Hz, H-6), 7.60 (1H, d, *J* = 8.8, H-12), 6.90 (1H, d, *J* = 8.8 Hz, H-5), 6.90 (1H, d, *J* = 8.8 Hz, H-11), 4.60 (1H, d, *J* = 3.6 Hz, H-1), 4.60 (1H, d, *J* = 3.6 Hz, H-7), 4.22 (1H, sl, H-6b), 4.22 (1H, sl, 12b), 3.86 (1H, d, *J* = 3.4 Hz, H-2), 3.86 (1H, d, *J* = 3.4, Hz, H-8). HRESIMS (positive mode) calcd. for C_20_H_13_O_6_ [M + H]^+^ *m*/*z* 349.0712, found: 349.0677.

Compound **4**, tricycloalternarene 3a, was obtained as a colorless oil. ^1^H NMR (400 MHz, CD_3_OD): δ (ppm) 5.02 (1H, m H-3), 5.31 (1H, s, H-8), 2.77 (1H, d, *J* = 3.6 Hz, H-11), 2.58 (1H, d, *J* = 16.4 Hz, H-9_α_), 2.55 (1H, m, H-12α), 2.55 (1H, m, H-17), 2.46 (d, *J* = 16.0 Hz, H-9β), 4.33 (1H, t, *J* = 5.6 Hz, H-15), 2.21 (1H, t, *J* = 6.4 Hz, H-16α), 2.18 (1H, m, H-12-β), 1.96 (1H, m, H-6), 1.92 (1H, m, H-16β), 1.84 (2H, m, H-4), 1.65 (3H, s, CH_3_), 1.55 (3H, s, CH_3_), 1.48 (3H, s, CH_3_), 1.45 (1H, m, H-5α), 1.28 (1H, m, H-5_β_), 0.93 (3H, d, *J* = 6.8 Hz, CH_3_). ^13^C NMR (100 MHz, CD_3_OD): δ (ppm) 196.8 (C-18), 169.9 (C-14), 150.4 (C-7), 131.5 (C-2), 124.6 (C-3), 119.8 (C-8), 108.0 (C-13), 88.9 (C-10), 66.6 (C-15), 46.8 (C-11), 45.0 (C-9), 35.0 (C-5), 33.7 (C-17), 32.4 (C-6), 29.0 (C-16), 26.0 (C-4), 25.8 (C-1), 23.8 (C-10′), 20.3 (C-6′), 17.8 (C-2′), 15.5 (C-12). HRESIMS (positive mode) calcd. for C_21_H_31_O_3_ [M + H]^+^ *m*/*z* 331.2273, found: 331.2303.

Compound **5**, tricycloalternarene 2b, was obtained as a white solid. ^1^H NMR (400 MHz, CD_3_OD): δ (ppm) 5.32 (1H, sl, H-8), 5.24 (1H, td, *J* = 6.4; 1.6 Hz), 4.02 (1H, dd, *J* = 12.8; 5.2 Hz, H-17), 3.95 (2H, sl, H-1), 2.77 (1H, m, H-11), 2.70 (1H, m, H-12α), 2.57 (1H, m, H-9α), 2.48 (1H, m, H-15α), 2.45 (1H, m, H-9β), 2.34 (1H, m, H-15β), 2.30 (1H, m, H-16α), 2.14 (1H, m, H-12β), 2.01 (1H, m, H-6), 1.70 (1H, m, H-16β), 1.90–1.98 (2H, m, H-4), 1.61 (3H, s, CH_3_), 1.47 (1H, m, H-5α), 1.43 (3H, s, CH_3_), 1.33 (1H, m, H-5β), 0.96 (1H, d, *J* = 6.8 Hz, H-6′). ^13^C NMR (100 MHz, CD_3_OD): δ (ppm) 197.9 (C-18), 173.0 (C-14), 150.2 (C-7), 135.4 (C-2), 125.6 (C-3), 120.1 (C-8), 105.2 (C-13), 88.9 (C-10), 71.1 (C-17), 68.9 (C-1), 46.7 (C-11), 45.2 (C-9), 34.8 (C-5), 31.3 (C-6), 29.6 (C-16), 27.9 (C-15), 25.0 (C-4), 23.4 (C-10′), 20.3 (C-6′), 15.1 (C-12), 13.8 (C-2′). HRESIMS (positive mode) calcd. for C_21_H_31_O_4_ [M + H]^+^ *m*/*z* 347.2222, found: 347.2226.

Compound **6**, tricycloalternarene 1b, was obtained as a white solid. ^1^H NMR (400 MHz, CD_3_OD): δ (ppm) 5.31 (1H, sl, H-8), 4.03 (1H, dd, *J =* 12.8; 5.6 Hz, H-17), 3.42 (2H, m, H-1), 2.74 (1H, m, H-11), 2.64 (1H, m, H-12α), 2.57 (1H, m, H-9α), 2.48 (1H, m, H-15α), 2.44 (1H, m, H-9β), 2.39 (1H, m, H-15β), 2.30 (1H, m, H-16α), 2.19 (1H, m, H-12β), 2.00 (1H, m, H-6), 1.73 (1H, m, H-16β), 1.56 (1H, m, H-2), 1.43 (1H, m, H-5α), 1.43 (3H, s, CH_3_), 1.24 (1H, m, H-5β), 1.31 (1H, m, H-3α), 1.24–1,3 (2H, m, H-4), 1.02 (1H, m, H-3β), 0.95 (3H, d, *J =* 7.2 Hz, CH_3_), 0.87 (3H, d, *J =* 6.8 Hz, CH_3_). ^13^C NMR (100 MHz, CD_3_OD): δ (ppm) 198.0 (C-18), 172.6 (C-14), 150.6 (C-7), 119.9 (C-8), 105.4 (C-13), 88.5 (C-10), 71.1 (C-17), 68.5 (C-1), 46.6 (C-11), 45.1 (C-9), 35.6 (C-2), 35.2 (C-5), 33.2 (C-3), 32.6 (C-6), 29.7 (C-16), 27.9 (C-15), 24.8 (C-4), 23.5 (C-10′), 20.3 (C-6′), 16.7 (C-2′), 15.5 (C-12). HRESIMS (positive mode) calcd. for C_21_H_33_O_4_ [M + H]^+^ *m*/*z* 349.2378, found: 349,2472.

Compound **7**, anthranilic acid, was obtained as a colorless crystal. ^1^H NMR (400 MHz, CD_3_OD): δ (ppm) 7.70 (1H, d, *J* = 8.0 Hz, H-7), 7.12 (1H, t, *J* = 8.0 Hz, H-6), 6.63 (1H, d, *J* = 8.0 Hz, H-4), 6.47 (1H, t, *J* = 8.0 Hz, H-5). ^13^C NMR (100 MHz, CD_3_OD): δ (ppm) 171.7 (C-1), 152.8 (C-3), 134.9 (C-2), 133.4 (C-6), 116.6 (C-4), 116.6 (C-7) 115.0 (C-5). HRESIMS (negative mode) calcd. for C_7_H_6_NO_2_ [M − H]^−^ *m*/*z* 136.0398, found: 136.0353.

Compound **8**, *o*-acetamidobenzoic acid, was obtained as a colorless crystal. ^1^H NMR (400 MHz, CD_3_OD): δ (ppm) 8.45 (1H, dd, *J*= 8.4; 0.8 Hz, H-7), 8.00 (1H, dd, *J*= 8.0; 1.6 Hz, H-4), 7.57 (1H, ddd, *J*= 8.6; 7.4; 1.6 Hz, H-6), 7.16 (1H, ddd, *J*= 7.9; 7.4; 1.1 Hz, H-5), (3H, s, CH3). ^13^C NMR (100 MHz, CD_3_OD): δ (ppm) 169.9 (C-1), 169.1 (C-8), 141.1 (C-3), 133.9 (C-5), 131.5 (C-7), 123.0 (C-6), 120.3 (C-4), 118.1 (C-2), 25.4 (C-9). HRESIMS (negative mode) calcd. for C_9_H_8_NO_3_ [M − H]^−^ 178.0510, found: 178.0553.

## 5. Conclusions

We report, for the first time, anti-inflammatory activity screening and an extensive metabolomics analysis of extracts providing from five endophytic fungi isolated from Sudanese medicinal plants. The anti-inflammatory activity screening has opened the way to discover active endophytic fungi crude extracts, and the metabolomics analysis has permitted us to detail their chemical richness. The HPTLC, GC-MS and LC-MS analyses combined with molecular networking data processing were carried out to allow the identification of different structures of metabolites such as fatty acids, carboxylic acids and derivatives, steroid derivatives, alkaloids, hydroxyanthraquinones, valerolactones and perylenequinones. Based on literature data, a number of molecules belonging to perylenequinone family could have anti-inflammatory activities.

We found that two extracts (extract C from *Aspergillus terreus* and extract A from *Alternaria alternata*) were able to inhibit the LPS pro-inflammatory effect on differentiated THP-1 cells. Moreover, these both extracts were able to set these cells in an anti-inflammatory state rendering them less sensible to the pro-inflammatory LPS effect.

Extract A from *Alternaria alternata* exhibited the strongest anti-inflammatory activity. Its purification showed the presence of three perylenequinones such as altertoxins, three terpenoids such as tricycloalternarenes and two derivatives of benzoic acid. These perylenequinones and in particular, altertoxin II could explain this anti-inflammatory activity.

However, due to limited literature data, the terpenoid compounds with the tricycloalternares structures, need also to be investigated for this biological property.

## Figures and Tables

**Table 1:** Effects of the five endophytic fungal extracts on the viability of differentiated THP-1 cells.

Sample Concentration (µg/mL)	% of Viable Cell	S.D.	Sample Concentration (µg/mL)	% of Viable Cells	S.D.	Sample Concentration (µg/mL)	% of Viable Cells	S.D.
Control	100.00	0	LPS	99.20	6.78			
Solv,5	104.76	9.40	LPS+Solv,5	99.48	3.48	Solv,5+LPS	101.26	9.33
Solv,10	105.56	8.05	LPS+Solv,10	100.57	7.18	Solv,10+LPS	99.96	6.32
Solv,20	106.86	7.32	LPS+Solv,20	97.62	7.27	Solv,20+LPS	100.43	8.78
Solv,40	104.39	8.52	LPS+Solv,40	99.25	8.47	Solv,40+LPS	99.48	5.54
Solv,60	99.18	4.53	LPS+Solv,60	97.61	7.70	Solv,60+LPS	100.59	6.53
A,5	104.42	8.84	LPS+A,5	101.13	10.48	A,5+LPS	101.05	9.02
A,10	99.41	6.26	LPS+A,10	102.26	8.92	A,10+LPS	103.24	4.20
A,20	101.37	4.76	LPS+A,20	98.44	8.13	A,20+LPS	98.20	8.93
A,40 *^,$$$^	79.71	6.76	LPS+A,40 *^,$$^	78.96	9.44	A,40+LPS *^,$$^	80.50	9.22
B,5	104.98	5.76	LPS+B,5	101.32	6.14	B,5+LPS	101.38	8.51
B,10	99.19	8.17	LPS+B,10	98.07	6.15	B,10+LPS	99.74	7.84
B,20	95.06	3.41	LPS+B,20	93.78	5.84	B,20+LPS	94.06	6.09
B,40	92.44	3.23	LPS+B,40	92.34	7.37	B,40+LPS	92.79	7.75
B,60 *^,$^	78.97	2.47	LPS+B,60 *^,$^	77.10	5.81	B,60+LPS *^,$^	77.48	7.02
C,5	103.90	9.54	LPS+C,5	102.45	8.99	C,5+LPS	99.93	7.34
C,10	95.76	7.68	LPS+C,10	97.11	8.35	C,10+LPS	102.68	5.86
C,20	103.64	9.39	LPS+C,20	100.36	9.63	C,20+LPS	104.14	7.36
C,40	101.28	5.03	LPS+C,40	98.34	9.05	C,40+LPS	100.17	8.96
C,60	99.74	6.00	LPS+C,60	99.04	9.08	C,60+LPS	99.82	7.67
D,5	100.76	9.07	LPS+D,5	98.38	6.86	D,5+LPS	100.14	9.09
D,10	104.59	5.59	LPS+D,10	99.12	2.36	D,10+LPS	98.03	7.28
D,20	92.76	4.75	LPS+D,20	87.77	8.47	D,20+LPS	91.84	7.68
D,40 *^,$^	85.07	8.77	LPS+D,40 *^,$^	83.89	9.00	D,40+LPS *^,$^	83.10	8.73
E,5	100.08	9.15	LPS+E,5	103.69	5.54	E,5+LPS	99.75	8.06
E,10	101.09	9.36	LPS+E,10	99.52	7.78	E,10+LPS	98.09	9.64
E,20	98.61	5.33	LPS+E,20	97.32	9.41	E,20+LPS	97.89	9.62
E,40	93.59	9.34	LPS+E,40	92.04	9.15	E,40+LPS	93.95	8.66
E,60 *^,$^	87.29	6.53	LPS+E,60 *^,$^	85.69	8.82	E,60+LPS *^,$$^	85.30	4.90

Five concentrations of these extracts (5, 10, 20, 40 and 60 µg/mL) were tested. Differentiated THP-1 cells were incubated either with these extracts alone or with 100 ng/mL of LPS added one hour before or after the endophytic fungal extract addition. Twenty-four hours later, THP-1 cell viability was analyzed via crystal violet assay and optical density (O.D.) was measured at 595 nm. “Control” corresponds to a THP-1 cell culture in which no product was added (negative control). “LPS” corresponds to a THP-1 cell culture in which only LPS was added. “Solv” corresponds to ethanol used to dissolve the endophytic fungal extracts. “LPS + endophytic fungal extract,5” indicates that LPS was added before endophytic fungal extract at a concentration of 5 µg/mL, while “endophytic fungal extract,5 + LPS” indicated that the endophytic fungal extract at a concentration of 5 µg/mL was added before LPS. The values with asterisk (*) indicate the first concentration for which the endophytic fungal extract became cytotoxic for THP-1 differentiated cells. This table is representative of six independent experiments performed in triplicate. Data are presented as the mean ± S.D. *t*-tests were used to identify statistically significant differences. ^$^ *p* < 0.05 vs. control; ^$$^ *p* < 0.01 vs. control; ^$$$^ *p* < 0.001 vs. control.

**Table 2:** Chemical composition of the five fungal endophyte extracts analyzed by GC-MS.

Compound Name	Chemical Formula	MW	rt (min)	RI	Similarity %	Extracts				
						A	B	C	D	E
3-*t*-Pentylcyclopentanone	C_10_H_18_O	154	11.31	1145	83	-	-	+	+	-
Thujopsene	C_15_H_24_	204	14.15	1416	85	+	-	-	-	-
Methyl 2-nonynoate	C_10_H_16_O_2_	168	14.56	1200	83	-	-	+	-	-
Dodecanal	C_12_H_24_O	184	17.5	1402	89	-	-	-	-	+
3-Isobutylhexahydropyrrolo[1,2-a]pyrazine-1,4-dione or cyclo (Pro-Leu)	C_11_H_18_N_2_O_2_	210	19.8	1699	92	+	-	-	-	+
Palmitic acid	C_16_H_32_O_2_	256	20.04	1968	95	+	+	-	+	+
11,14-Eicosadienoic acid, methyl ester	C_21_H_38_O_2_	322	21.6	2292	88	-	-	+	-	+
Linoleic acid	C_18_H_32_O_2_	280	21.7	2183	93	+	+	+	+	+
Oleic Acid	C_18_H_34_O_2_	282	21.72	2175	87	+	+	-	+	+
Stearic acid	C_18_H_36_O_2_	284	21.95	2167	88	+	-	-	-	+
3-Benzylhexahydropyrrolo[1,2-a]pyrazine-1,4-dione or cyclo (Phe-Pro)	C_14_H_16_N_2_O_2_	244	23.9	2138	80	-	-	-	-	+
trans-Squalene	C_30_H_50_	410	27.08	2914	87	+	-	-	-	+
Lovastatin	C_24_H_36_O_5_	404	28.02	3091	77	-	+	+	+	-
Ergosterol	C_28_H_44_O	396	29.74	2679	80	-	-	-	-	+
Hippuric-benzaldehyde azalactone	C_16_H_11_NO_2_	249	32.94	2266	82	-	+	+	+	-
Total						7	6	6	7	9

MW = molecular weight; rt (min) = retention time expressed in minutes; RI = retention index; (-) = no compound detected; (+) = compound detected. After identification via GC–MS and comparison with NIST Mass Spectral Library (V2.0-19/05/2011), the chemical names were confirmed using the PubChem application.

**Table 3:** Chemical composition of the five fungal endophyte extracts analyzed by GC-MS after derivatization.

Compound Name	Chemical Formula	MW	rt (min)	RI	Similarity %	Extracts				
						A	B	C	D	E
2-Ketoisocaproic acid, trimethylsilyl ester	C_9_H_18_O_3_Si	202	9.06	1065	86	+	-	-	-	-
Benzeneacetic acid, trimethylsilyl ester	C_11_H_16_O_2_Si	208	12.15	1269	90	+	-	-	-	+
Succinic acid, di(trimethylsilyl) ester	C_10_H_22_O_4_Si_2_	262	12.4	1170	81	+	-	-	-	+
Acide itaconique (tms)	C_11_H_22_O_4_Si_2_	274	12.80	1236	80	-	-	+	-	-
Fumaric acid, bis(trimethylsilyl) ester	C_10_H_20_O_4_Si_2_	260	12.94	1178	81	-	-	-	-	+
Malic acid, O-trimethylsilyl-, bis(trimethylsilyl) ester	C_13_H_30_O_5_Si_3_	350	14.74	1390	87	+	-	-	-	+
Benzoic acid, 4-[(trimethylsilyl)oxy]-, trimethylsilyl ester	C_13_H_22_O_3_Si_2_	282	16.44	1467	85	-	-	+	-	+
Benzeneacetic acid, 4-[(trimethylsilyl)oxy]-, trimethylsilyl ester	C_14_H_24_O_3_Si_2_	296	16.6	1566	82	+	-	-	-	-
Phenylpyruvic acid, bis(trimethylsilyl)	C_15_H_24_O_3_Si_2_	308	17.33	1637	83	+	-	-	-	-
2-Propenoic acid, 2-[(trimethylsilyl)oxy]	C_18_H_32_O_4_Si_3_	396	20.8	1935	76	-	+	-	-	-
Palmitic acid, trimethylsilyl ester	C_16_H_32_O_2_	256	20.9	1987	91	+	+	-	+	+
Linoleic acid trimethylsilyl ester	C_21_H_40_O_2_Si	352	22.34	2202	93	+	+	+	+	+
Oleic acid, trimethylsilyl ester	C_21_H_42_O_2_Si	354	22.40	2194	90	+	+	-	+	+
Stearic acid, trimethylsilyl ester	C_21_H_44_O_2_Si	356	22.63	2186	90	+	-	-	-	+
1-Monooleoylglycerol trimethylsilyl ether	C_27_H_56_O_4_Si_2_	500	26.65	2788	81	-	-	-	-	+
Lovastatin	C_24_H_36_O_5_	404	29.01	3012	96	-	+	+	+	-
Bis(trimethylsilyl) 3-methyl-3-trimethylsilyloxypentanedioate	C_15_H_34_O_5_Si_3_	378	29.68	1568	70	-	-	+	+	-
(22E)-3-[(trimethylsilyl)oxy]ergosta-5,7,22-triene	C_31_H_52_OSi	468	29.81	2708	70	-	-	+	-	+
Total						10	4	6	5	11

MW = molecular weight; rt (min) = retention time expressed in minutes; RI = retention index; (-) = no compound detected; (+) = compound detected. After identification via GC–MS and comparison with NIST Mass Spectral Library (V2.0-19/05/2011), the chemical names were confirmed using PubChem application.

## Supplementary Materials

The following supporting information can be downloaded at: https://www.mdpi.com/article/10.3390/molecules28186531/s1, Figure S1: Endophytic fungus *Alternaria alternate*; Figure S2: Chemical structure of compound **1**, altertoxin I; Figure S3: HR-ESI-MS spectrum of compound **1**, altertoxin I; Figure S4: ^1^H-NMR spectrum of compound **1**, altertoxin I, in CD_3_OD (400 MHz); Figure S5: ^13^C-NMR spectrum of compound **1**, altertoxin I, in CD_3_OD (100 MHz); Figure S6: ^1^H-^1^H COSY spectrum of compound **1**, altertoxin I, in CD_3_OD; Figure S7: HSQC spectrum of compound **1**, altertoxin I, in CD_3_OD; Figure S8: HMBC spectrum of compound **1**, altertoxin I, in CD_3_OD; Figure S9: Chemical structure of compound **2**, altertoxin II; Figure S10: HR-ESI-MS spectrum of compound **2**, altertoxin II; Figure S11: ^1^H-NMR spectrum of compound **2**, altertoxin II, in CD_3_OD (400 MHz); Figure S12: ^13^C-NMR spectrum of compound **2**, altertoxin II, in CD_3_OD (100 MHz); Figure S13: ^1^H-^1^H COSY spectrum of compound **2**, altertoxin II, in CD_3_OD; Figure S14: HSQC spectrum of compound **2**, altertoxin II, in CD_3_OD; Figure S15: HMBC spectrum of compound **2**, altertoxin II, in CD_3_OD; Figure S16: Chemical structure of compound **3**, altertoxin III; Figure S17: HR-ESI-MS spectrum of compound **3**, altertoxin III; Figure S18: ^1^H-NMR spectrum of compound **3**, altertoxin III, in CDCl_3_ (400 MHz); Figure S19: Zoom ^1^H-NMR spectrum of compound **3**, altertoxin III, in CDCl_3_ (400 MHz); Figure S20: HSQC spectrum of compound **3**, altertoxin III, in CDCl_3_; Figure S21: Chemical structure of compound **4**, Tricycloalternarene 3a; Figure S22: HR-ESI-MS spectrum of compound **4**, Tricycloalternarene 3a; Figure S23: ^1^H-NMR spectrum of compound **4**, Tricycloalternarene 3a, in CDCl_3_ (400 MHz); Figure S24: ^13^C-NMR spectrum of compound **4**, Tricycloalternarene 3a, in CDCl_3_ (100 MHz); Figure S25: ^1^H-^1^H COSY spectrum of compound **4**, Tricycloalternarene 3a, in CDCl_3_; Figure S26: HSQC spectrum of compound **4**, Tricycloalternarene 3a, in CDCl_3_; Figure S27: HMBC spectrum of compound **4**, Tricycloalternarene 3a, in CDCl_3_; Figure S28: Zoom of HMBC spectrum of compound **4**, Tricycloalternarene 3a, in CDCl_3_; Figure S29: Chemical structure of compound **5**, Tricycloalternarene 2b; Figure S30: HR-ESI-MS spectrum of compound **5**, Tricycloalternarene 2b, Figure S31: ^1^H-NMR spectrum of compound **5**, Tricycloalternarene 2b, in CDCl_3_ (400 MHz); Figure S32: ^13^C-NMR spectrum of compound **5**, Tricycloalternarene 2b, in CDCl_3_ (100 MHz); Figure S33: HSQC spectrum of compound **5**, Tricycloalternarene 2b, in CDCl_3_; Figure S34: HMBC spectrum of compound **5**, Tricycloalternarene 2b, in CDCl_3_; Figure S35: Chemical structure of compound **6**, Tricycloalternarene 1b; Figure S36: HR-ESI-MS spectrum of compound **6**, Tricycloalternarene 1b; Figure S37: ^1^H-NMR spectrum of compound **6**, Tricycloalternarene 1b, in CDCl_3_ (400 MHz); Figure S38: Zoom ^1^H-NMR spectrum of compound **6**, Tricycloalternarene 1b, in CDCl_3_ (400 MHz); Figure S39: ^13^C-NMR spectrum of compound **6**, Tricycloalternarene 1b, in CDCl_3_ (100 MHz); Figure S40: HSQC spectrum of compound **6**, Tricycloalternarene 1b, in CDCl_3_; Figure S41: Chemical structure of compound **7**, Anthranilic acid; Figure S42: HR-ESI-MS spectrum of compound **7**, Anthranilic acid; Figure S43: ^1^H-NMR spectrum of compound **7**, Anthranilic acid, in CD_3_OD (400 MHz); Figure S44: ^13^C-NMR spectrum of compound **7**, Anthranilic acid, in CD_3_OD (100 MHz); Figure S45: HSQC spectrum of compound **7**, Anthranilic acid, in CD_3_OD; Figure S46: HMBC spectrum of compound **7**, Anthranilic acid, in CDCl_3_; Figure S47: Chemical structure of compound **8**, *o*-acetamidobenzoic acid; Figure S48: HR-ESI-MS spectrum of compound **8**, *o*-acetamidobenzoic acid, Figure S49: ^1^H-NMR spectrum of compound **8**, *o*-acetamidobenzoic acid, in DMSO-*d*_6_ (400 MHz); Figure S50: Zoom ^1^H-NMR spectrum of compound **8**, *o*-acetamidobenzoic acid, in DMSO-*d*_6_ (400 MHz); Figure S51: ^13^C-NMR spectrum of compound **8**, *o*-acetamidobenzoic acid, in DMSO-*d*_6_ (100 MHz); Figure S52: HMBC spectrum of compound **8**, *o*-acetamidobenzoic acid, in DMSO-*d*_6_.

## Sample Availability

Samples of the compounds **1**–**8** are available from the authors.
