# Supplementary material for: Screening of Anti-Inflammatory Activity and Metabolomics Analysis of Endophytic Fungal Extracts; Identification and Characterization of Perylenequinones and Terpenoids from the Interesting Active Alternaria Endophyte
Source: Molecules. 2023 Sep 9;28(18):6531. doi: 10.3390/molecules28186531 (PMC10534442; doi:10.3390/molecules28186531)
Supplement: Supplementary file 1 [file molecules-28-06531-s001.zip › molecules-2550964-supplementary.pdf]

Supplementary materials

Screening of Anti-Inflammatory Activity and Metabolomics Analysis of Endophytic Fungal Extracts; Identification and Characterization of Perylenequinones and Terpenoids from the Interesting Active Alternaria Endophyte

Rosella Spina*[1], Armelle Ropars[2], Sihem Bouazzi[3], Safa Dadi[3], Pascal Lemiere[3], François Dupire[3], Afra Khiralla[4], Sakina Yagi[5], Jean-Pol Frippiat[2], Dominique Laurain-Mattar[1]

[1] Université de Lorraine, INRAE, LAE, F-54000 Nancy, France; rosella.spina@univ-lorraine.fr (R.S.); dominique.mattar@univ-lorraine.fr (D.L-M.);

[2] Université de Lorraine, SIMPA, F-54000 Nancy, France; armelle.ropars@univ-lorraine.fr (A.R.); jean-pol.frippiat@univ-lorraine.fr (J-P.F.);

[3] Université de Lorraine, CNRS, L2CM, F-54000 Nancy, France; bouazzi-sihem@hotmail.com (S.B.); dadi.safa@gmail.com (S.D.); pascal.lemiere@uiv-lorraine.fr (P.L.); francois.dupire@univ-lorraine.fr (F.D.);

[4] Botany Department, Faculty of Sciences and Technologies, Shendi University, 11111 Shendi, Sudan; aafraa21@hotmail.com (A.K.);

[5] Department of Botany, Faculty of Science, University of Khartoum, 11115 Khartoum, Sudan; sakinayagi@gmail.com (S.Y.).

* Correspondence: rosella.spina@univ-lorraine.fr. (R.S.).

Contents

Figure S1: Endophytic fungus *Alternaria alternata*

Figure S2: Chemical structure of compound **1,** altertoxin I

Figure S3: HR-ESI-MS spectrum of compound **1**, altertoxin I

Figure S4: ^{1}H-NMR spectrum of compound **1,** altertoxin I, in CD_3OD (400 MHz)

Figure S5: ^{13}C-NMR spectrum of compound **1,** altertoxin I, in CD_3OD (100 MHz)

Figure S6: ^{1}H-^{1}H COSY spectrum of compound **1,** altertoxin I, in CD_3OD

Figure S7: HSQC spectrum of compound **1,** altertoxin I, in CD_3OD

Figure S8: HMBC spectrum of compound **1,** altertoxin I, in CD_3OD

Figure S9: Chemical structure of compound **2,** altertoxin II

Figure S10: HR-ESI-MS spectrum of compound **2**, altertoxin II

Figure S11: ^{1}H-NMR spectrum of compound **2,** altertoxin II, in CD_3OD (400 MHz)

Figure S12: ^{13}C-NMR spectrum of compound **2,** altertoxin II, in CD_3OD (100 MHz)

Figure S13: ^{1}H-^{1}H COSY spectrum of compound **2,** altertoxin II, in CD_3OD

Figure S14: HSQC spectrum of compound **2,** altertoxin II, in CD_3OD

Figure S15: HMBC spectrum of compound **2,** altertoxin II, in CD_3OD

Figure S16: Chemical structure of compound **3,** altertoxin III

Figure S17: HR-ESI-MS spectrum of compound **3**, altertoxin III

Figure S18: ^{1}H-NMR spectrum of compound **3,** altertoxin III, in $CDCl_3$ (400 MHz)

Figure S19: Zoom ^{1}H-NMR spectrum of compound **3,** altertoxin III, in $CDCl_3$ (400 MHz)

Figure S20: HSQC spectrum of compound **3,** altertoxin III, in $CDCl_3$

Figure S21: Chemical structure of compound **4,** Tricycloalternarene 3a

Figure S22: HR-ESI-MS spectrum of compound **4**, Tricycloalternarene 3a

Figure S23: ^{1}H-NMR spectrum of compound **4,** Tricycloalternarene 3a, in $CDCl_3$ (400 MHz)

Figure S24: ^{13}C-NMR spectrum of compound **4,** Tricycloalternarene 3a, in $CDCl_3$ (100 MHz)

Figure S25: ^{1}H-^{1}H COSY spectrum of compound **4,** Tricycloalternarene 3a, in $CDCl_3$

Figure S26: HSQC spectrum of compound **4,** Tricycloalternarene 3a, in $CDCl_3$

Figure S27: HMBC spectrum of compound **4,** Tricycloalternarene 3a, in $CDCl_3$

Figure S28: Zoom of HMBC spectrum of compound **4,** Tricycloalternarene 3a, in $CDCl_3$

Figure S29: Chemical structure of compound **5,** Tricycloalternarene 2b

Figure S30: HR-ESI-MS spectrum of compound **5**, Tricycloalternarene 2b

Figure S31: ^{1}H-NMR spectrum of compound **5,** Tricycloalternarene 2b, in $CDCl_3$ (400 MHz)

Figure S32: ^{13}C-NMR spectrum of compound **5,** Tricycloalternarene 2b, in $CDCl_3$ (100 MHz)

Figure S33: HSQC spectrum of compound **5,** Tricycloalternarene 2b, in $CDCl_3$

Figure S34: HMBC spectrum of compound **5,** Tricycloalternarene 2b, in $CDCl_3$

Figure S35: Chemical structure of compound **6,** Tricycloalternarene 1b

Figure S36: HR-ESI-MS spectrum of compound **6**, Tricycloalternarene 1b

Figure S37: ^{1}H-NMR spectrum of compound **6,** Tricycloalternarene 1b, in $CDCl_3$ (400 MHz)

Figure S38: Zoom ^{1}H-NMR spectrum of compound **6,** Tricycloalternarene 1b, in $CDCl_3$ (400 MHz)

Figure S39: ^{13}C-NMR spectrum of compound **6,** Tricycloalternarene 1b, in $CDCl_3$ (100 MHz)

Figure S40: HSQC spectrum of compound **6,** Tricycloalternarene 1b, in $CDCl_3$

Figure S41: Chemical structure of compound **7,** Anthranilic acid

Figure S42: HR-ESI-MS spectrum of compound **7**, Anthranilic acid

Figure S43: ^{1}H-NMR spectrum of compound **7,** Anthranilic acid, in CD_3OD (400 MHz)

Figure S44: ^{13}C-NMR spectrum of compound **7,** Anthranilic acid, in CD_3OD (100 MHz)

Figure S45: HSQC spectrum of compound **7,** Anthranilic acid, in CD_3OD

Figure S46: HMBC spectrum of compound **7,** Anthranilic acid, in $CDCl_3$

Figure S47: Chemical structure of compound **8,** *o*-acetamidobenzoic acid

Figure S48: HR-ESI-MS spectrum of compound **8**, *o*-acetamidobenzoic acid

Figure S49: ^{1}H-NMR spectrum of compound **8,** *o*-acetamidobenzoic acid, in DMSO-d_6 (400 MHz)

Figure S50: Zoom ^{1}H-NMR spectrum of compound **8,** *o*-acetamidobenzoic acid, in DMSO-d_6 (400 MHz)

Figure S51: ^{13}C-NMR spectrum of compound **8,** *o*-acetamidobenzoic acid, in DMSO-d_6 (100 MHz)

Figure S52: HMBC spectrum of compound **8,** *o*-acetamidobenzoic acid, in DMSO-d_6

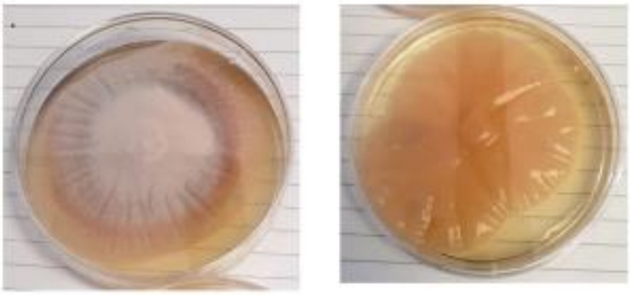

Figure S1: Endophytic fungus *Alternaria alternata*.

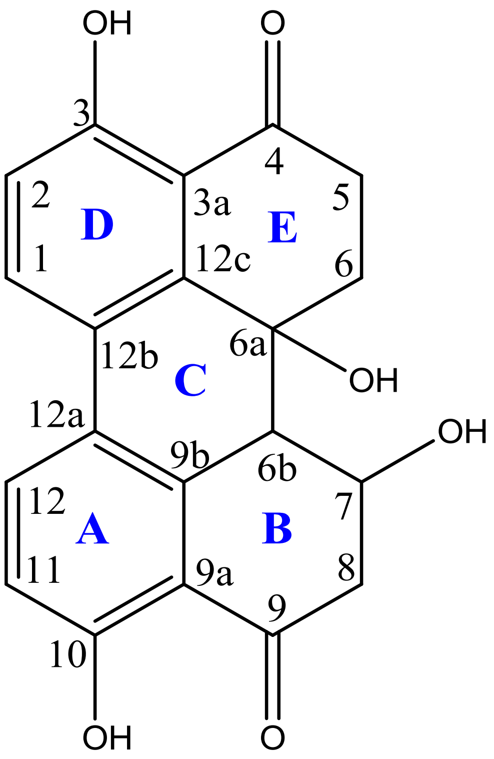

Figure S2: Chemical structure of compound **1,** altertoxin I

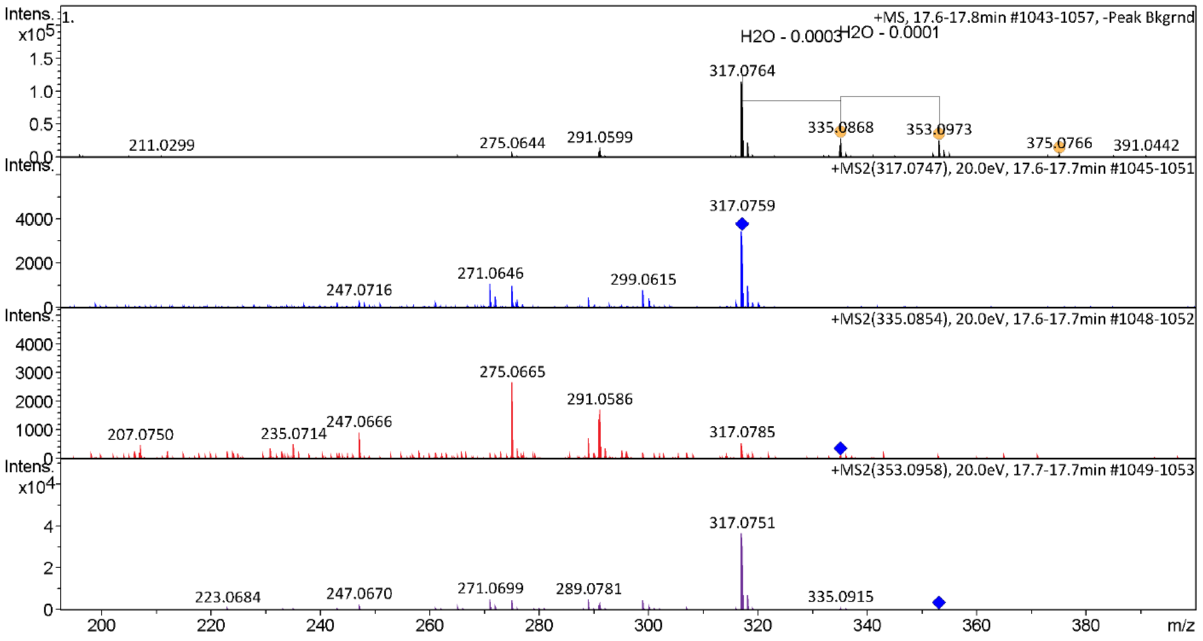

Figure S3: HR-ESI-MS spectrum of compound **1**, altertoxin

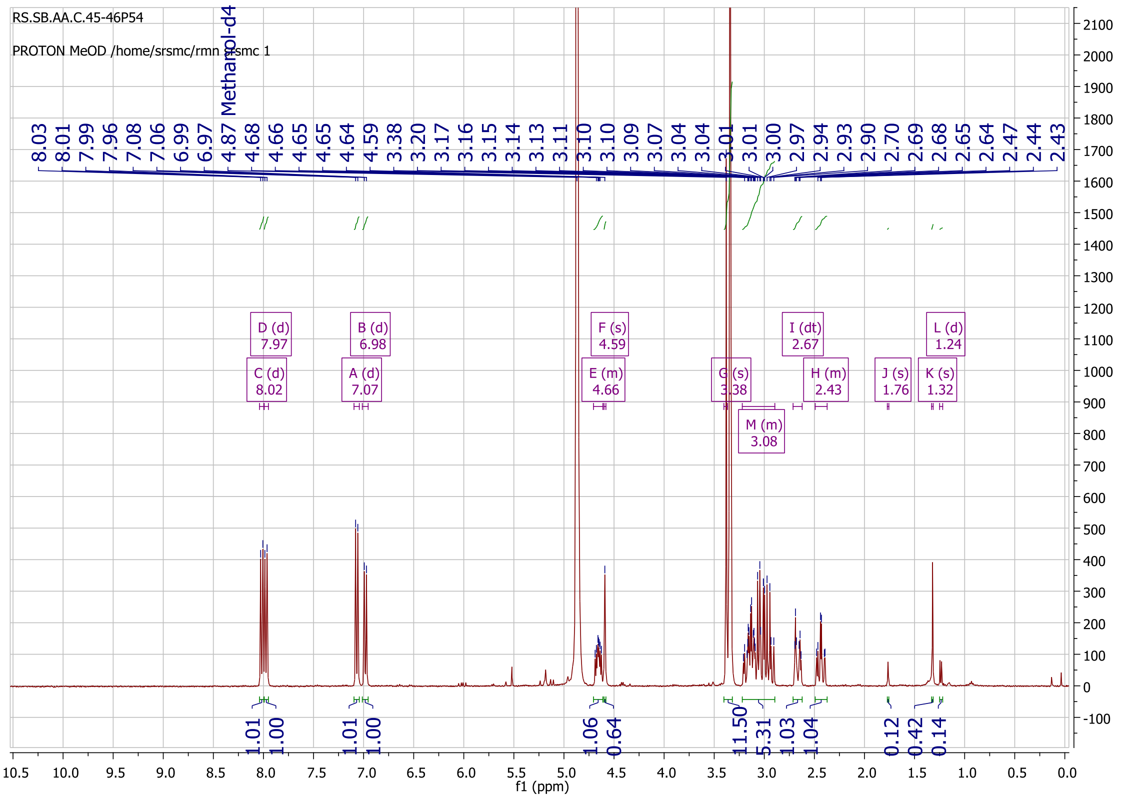

Figure S4: ^{1}H-NMR spectrum of compound **1,** altertoxin I, in CD_3OD (400 MHz)

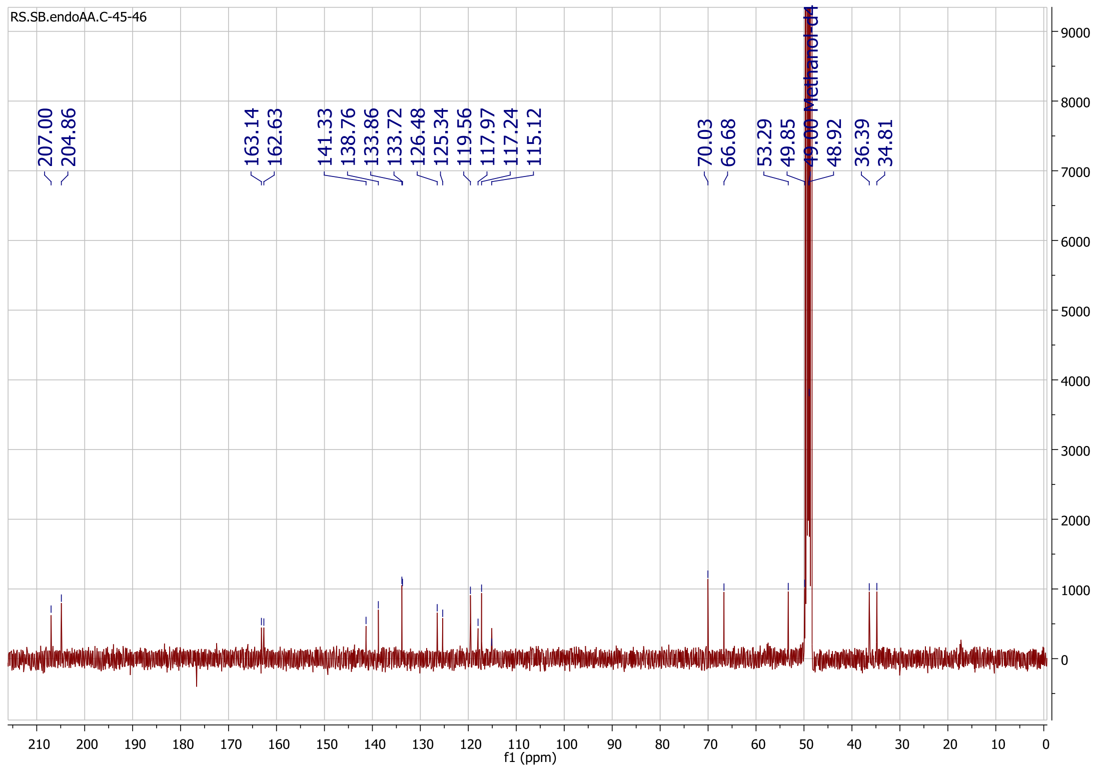

Figure S5: ^{13}C-NMR spectrum of compound **1,** altertoxin I, in CD_3OD (100 MHz)

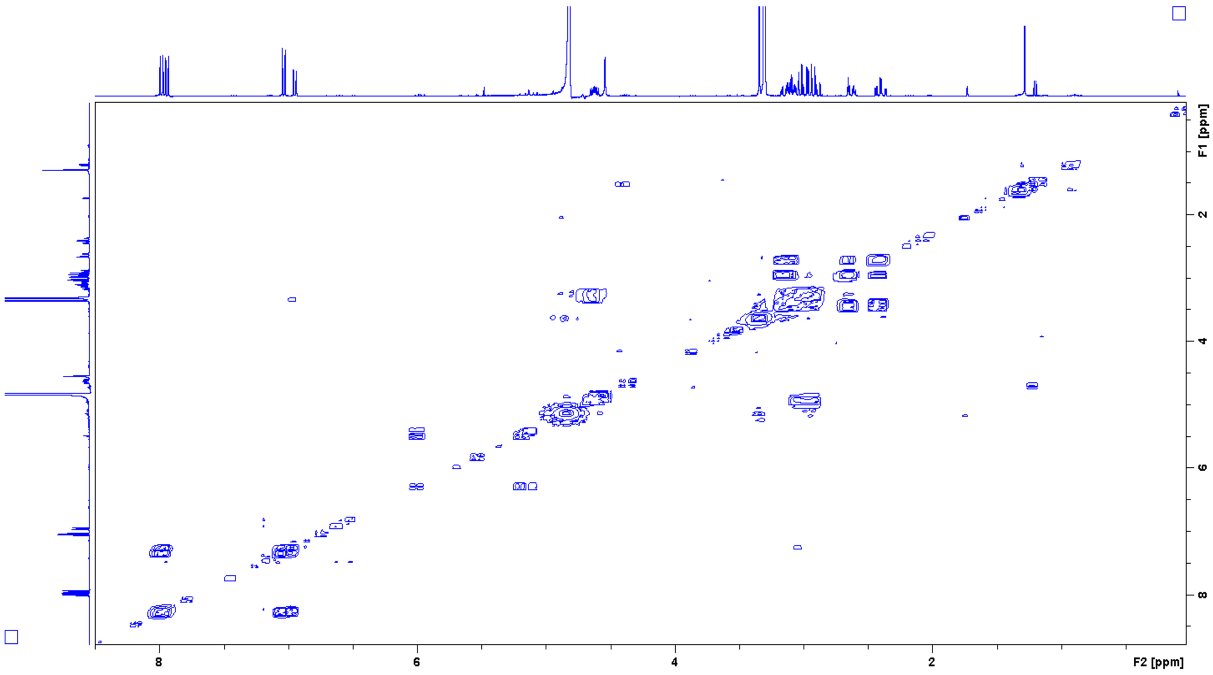

Figure S6: ^{1}H-^{1}H COSY spectrum of compound **1,** altertoxin I, in CD_3OD

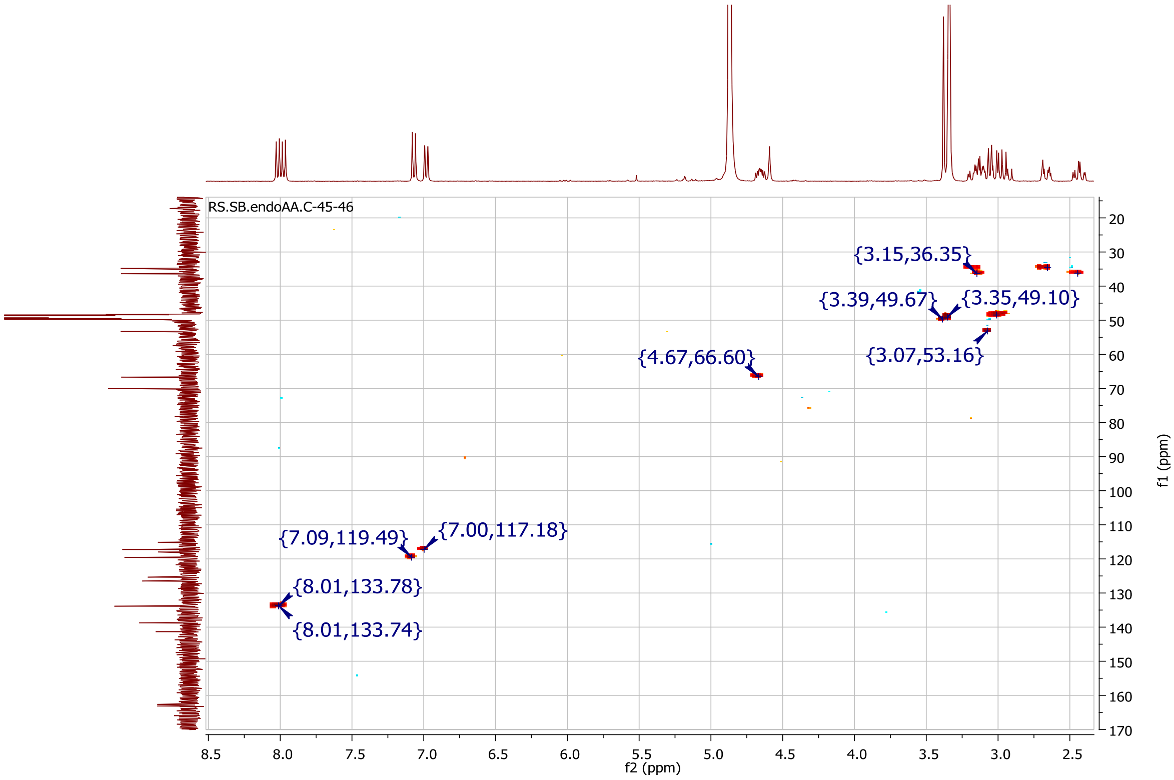

Figure S7: HSQC spectrum of compound **1,** altertoxin I, in CD_3OD

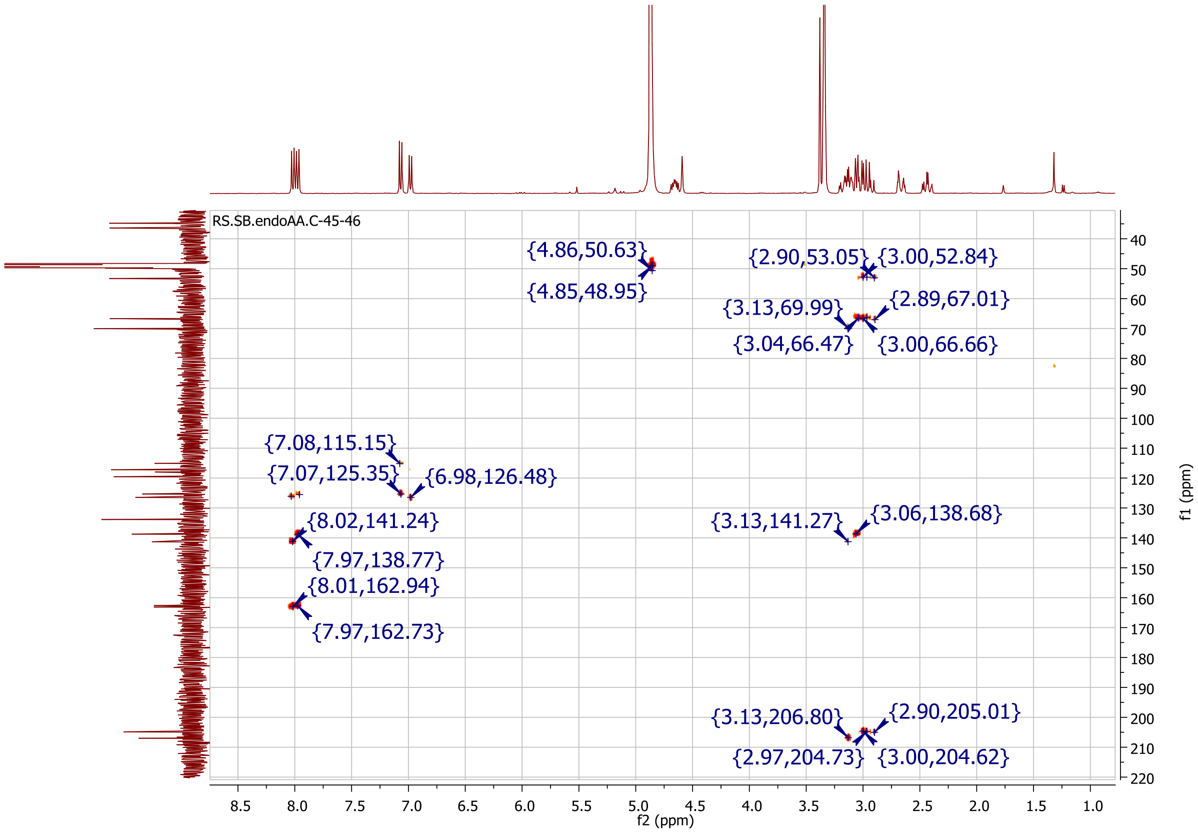

Figure S8: HMBC spectrum of compound **1,** altertoxin I, in CD_3OD

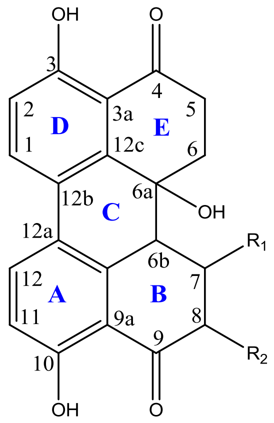

Figure S9: Chemical structure of compound **2,** altertoxin II

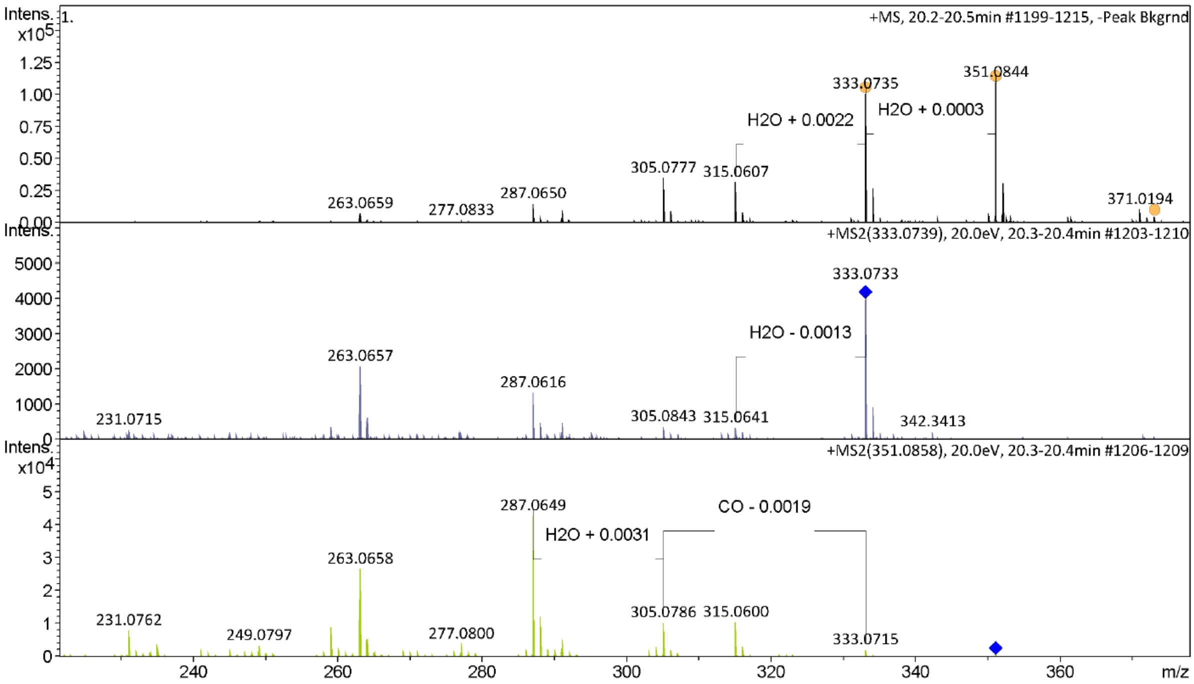

Figure S10: HR-ESI-MS spectrum of compound **2**, altertoxin II

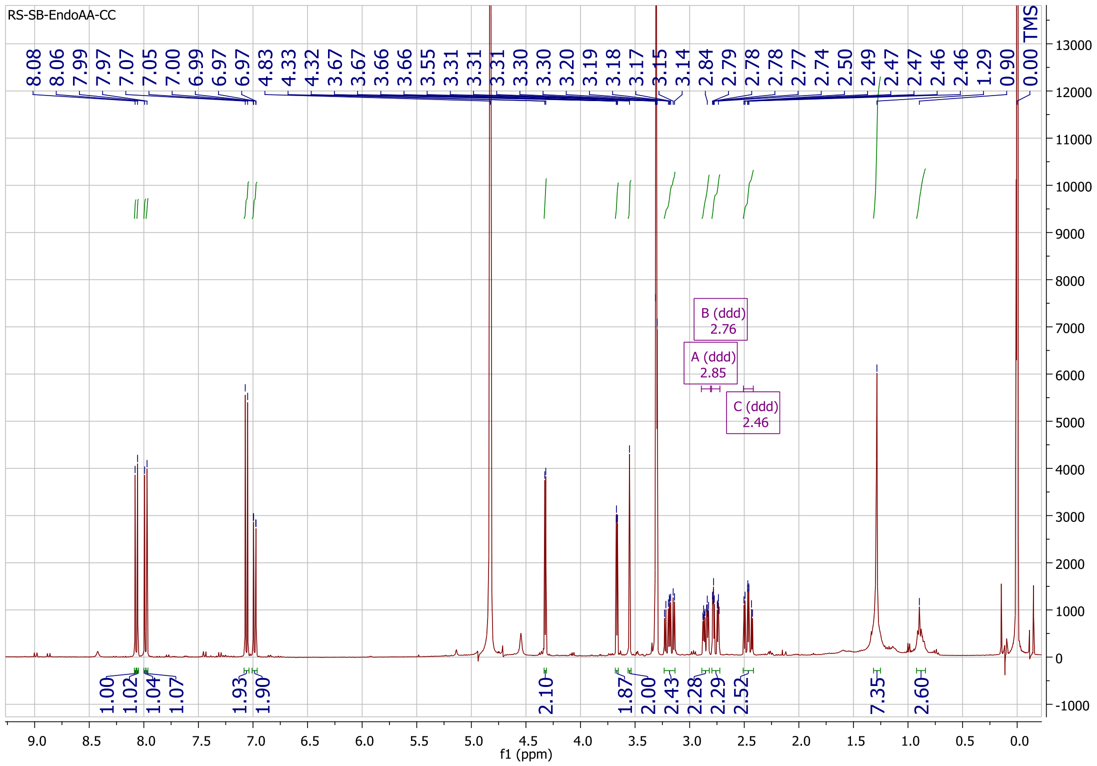

Figure S11: ^{1}H-NMR spectrum of compound **2,** altertoxin II, in CD_3OD (400 MHz)

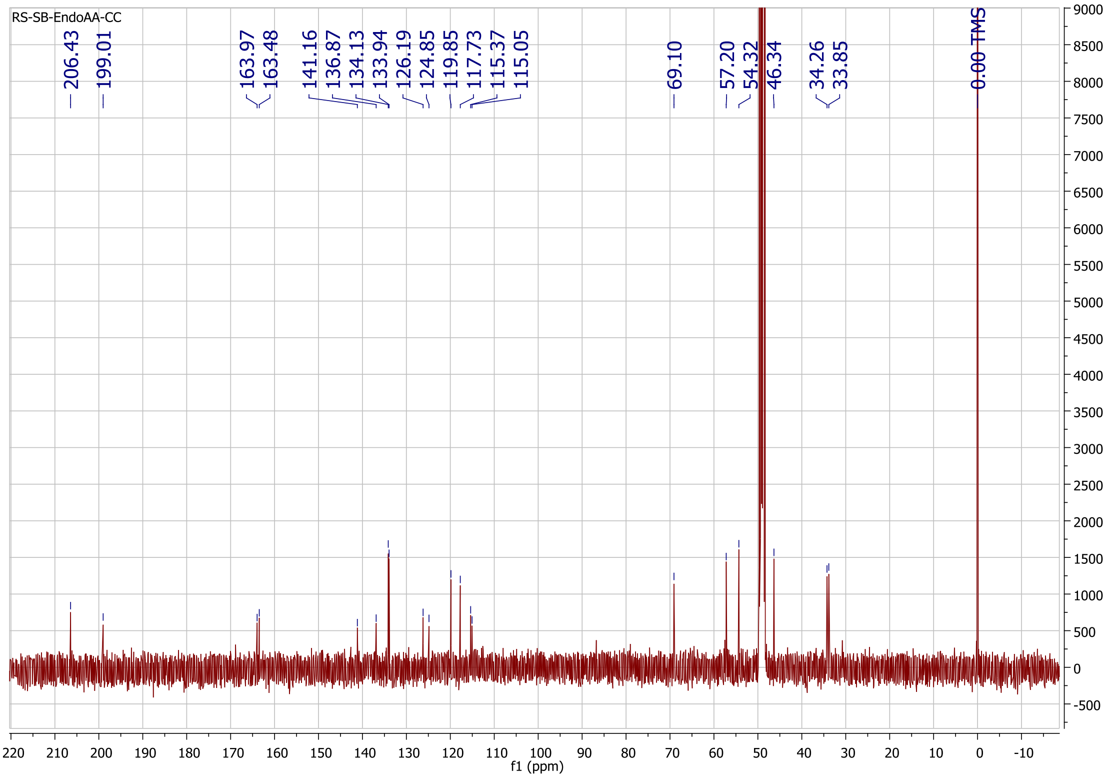

Figure S12: ^{13}C-NMR spectrum of compound **2,** altertoxin II, in CD_3OD (100 MHz)

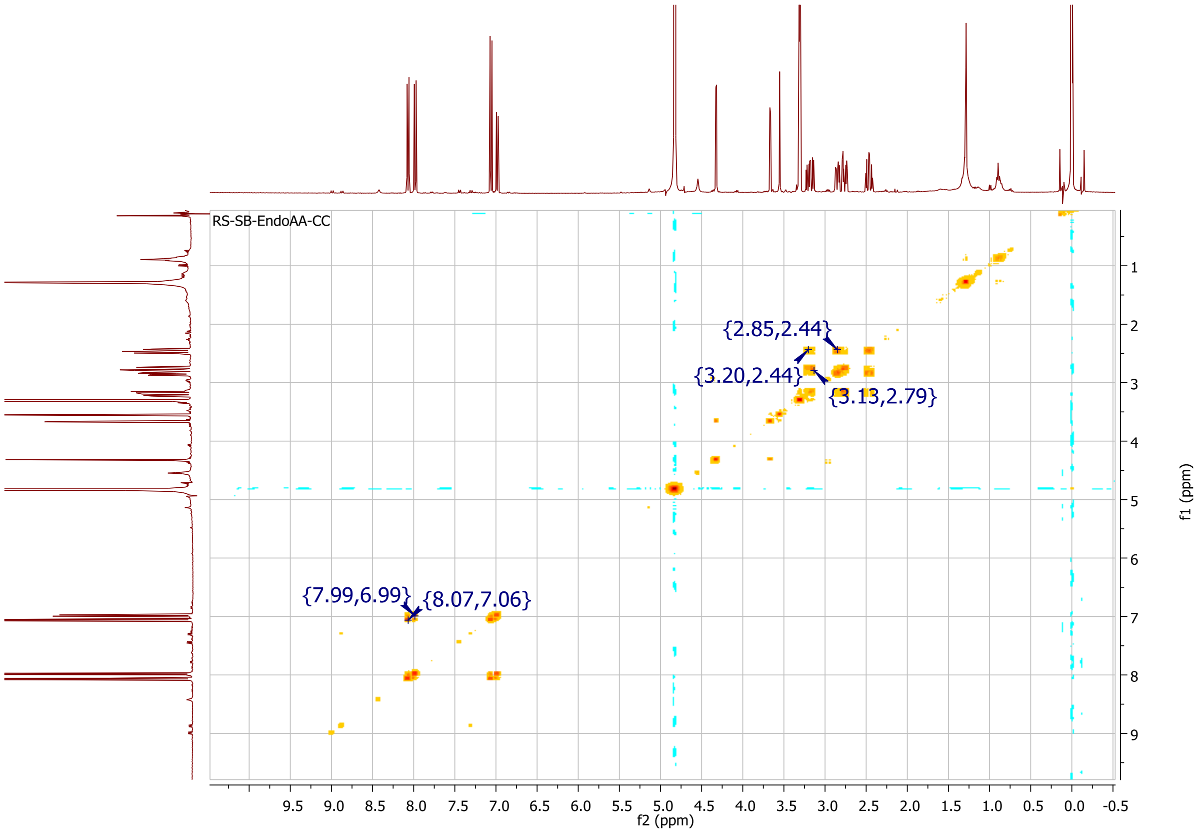

Figure S13: ^{1}H-^{1}H COSY spectrum of compound **2,** altertoxin II, in CD_3OD

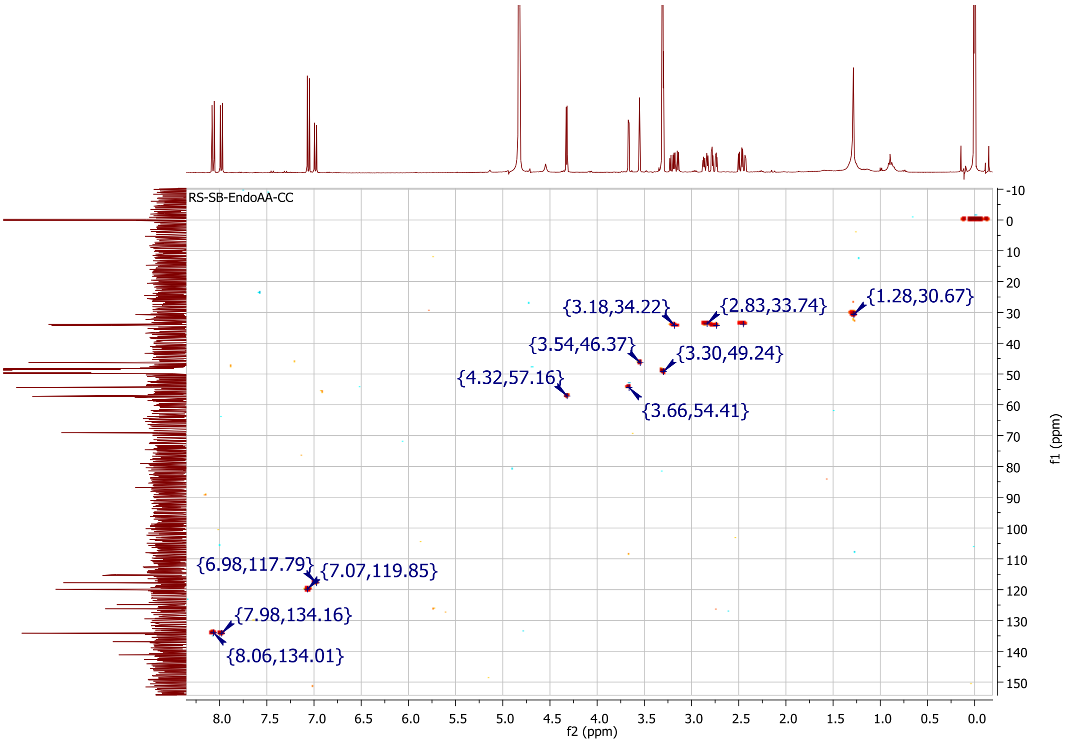

Figure S14: HSQC spectrum of compound **1,** altertoxin II, in CD_3OD

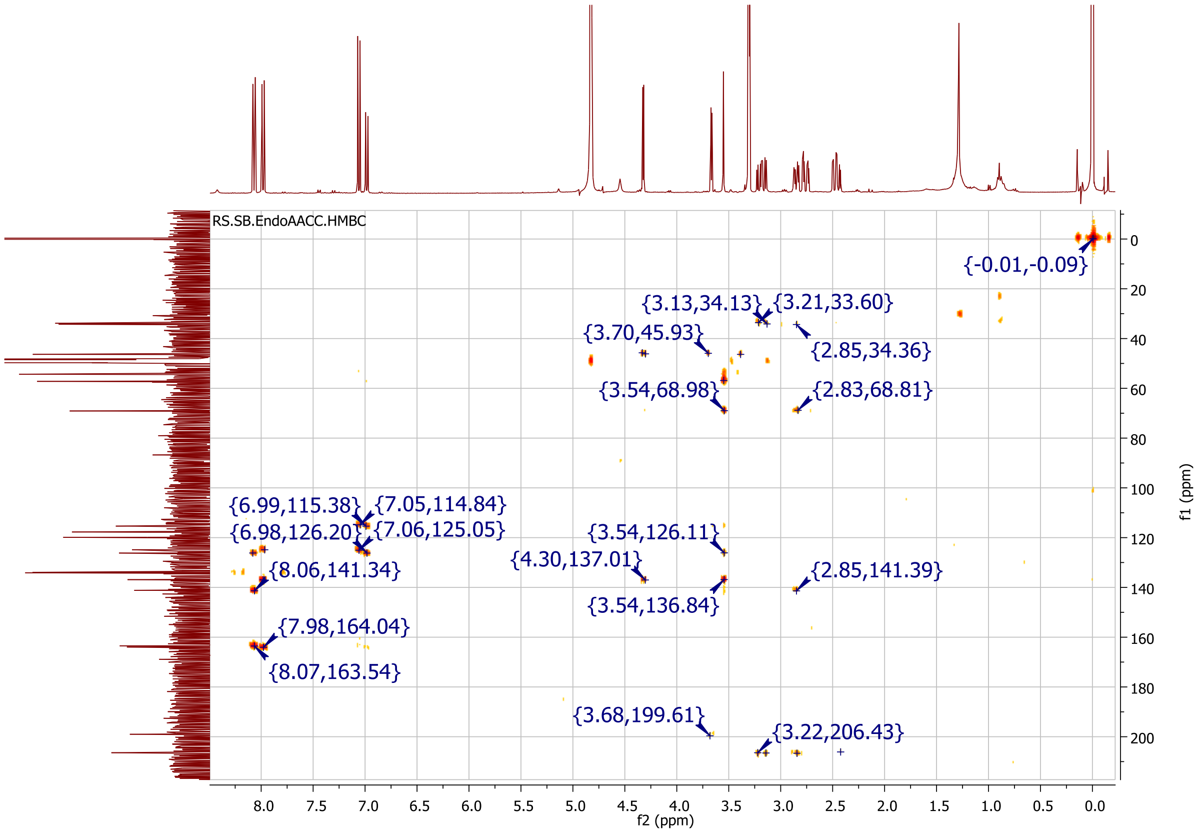

Figure S15: HMBC spectrum of compound **1,** altertoxin II, in CD_3OD

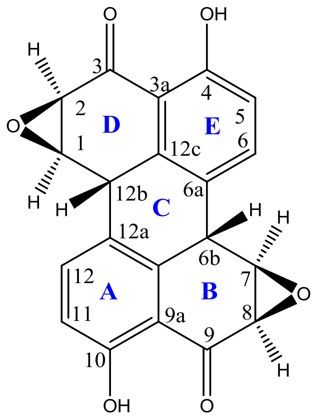

Figure S16: Chemical structure of compound **3,** altertoxin III

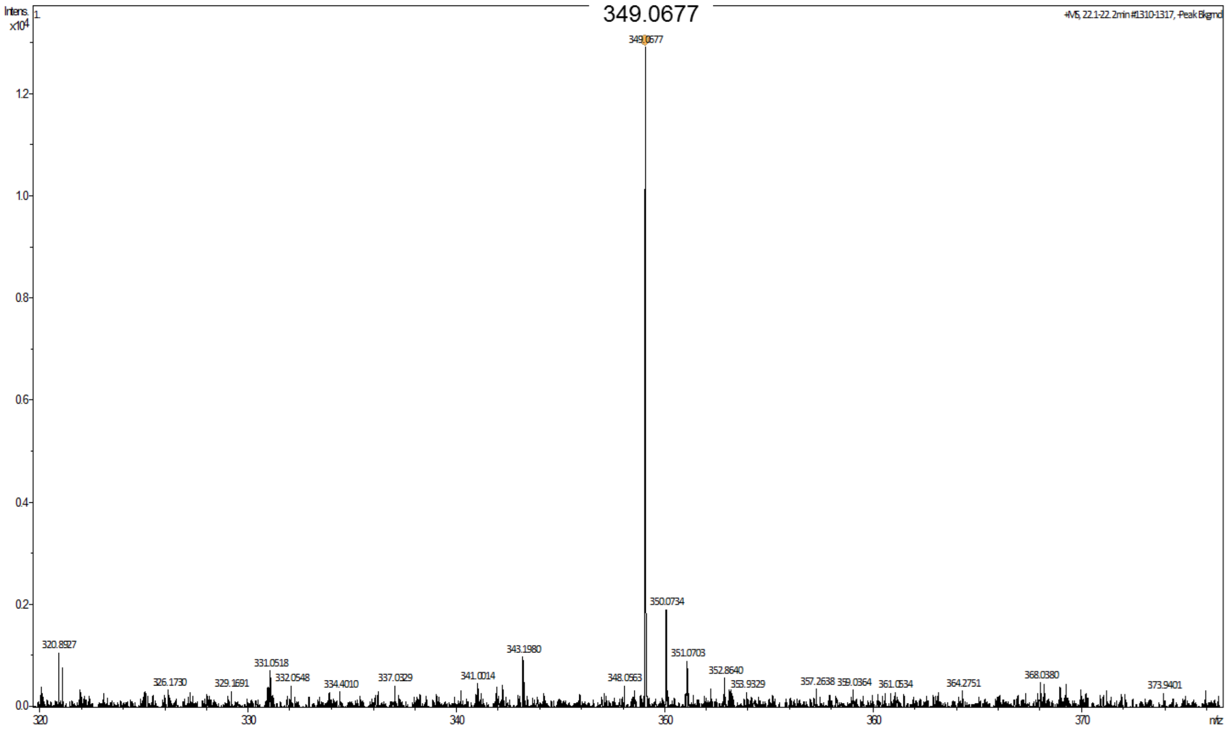

Figure S17: HR-ESI-MS spectrum of compound **3**, altertoxin III

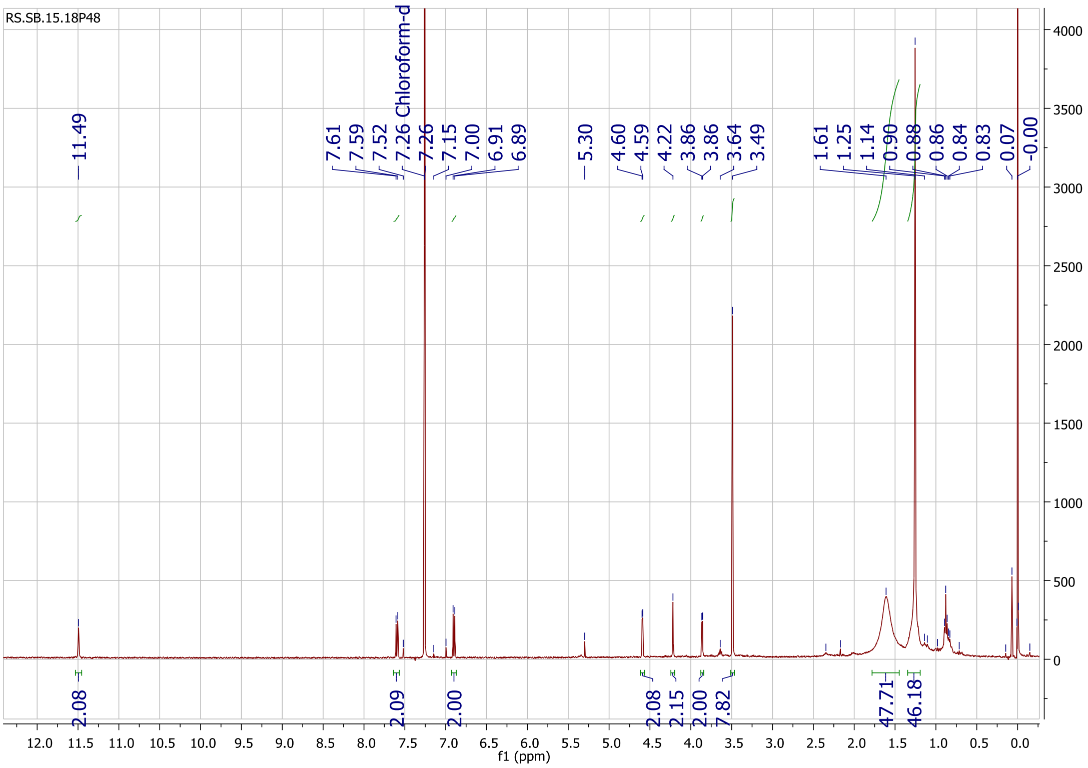

Figure S18: ^{1}H-NMR spectrum of compound **3,** altertoxin III, in $CDCl_3$ (400 MHz)

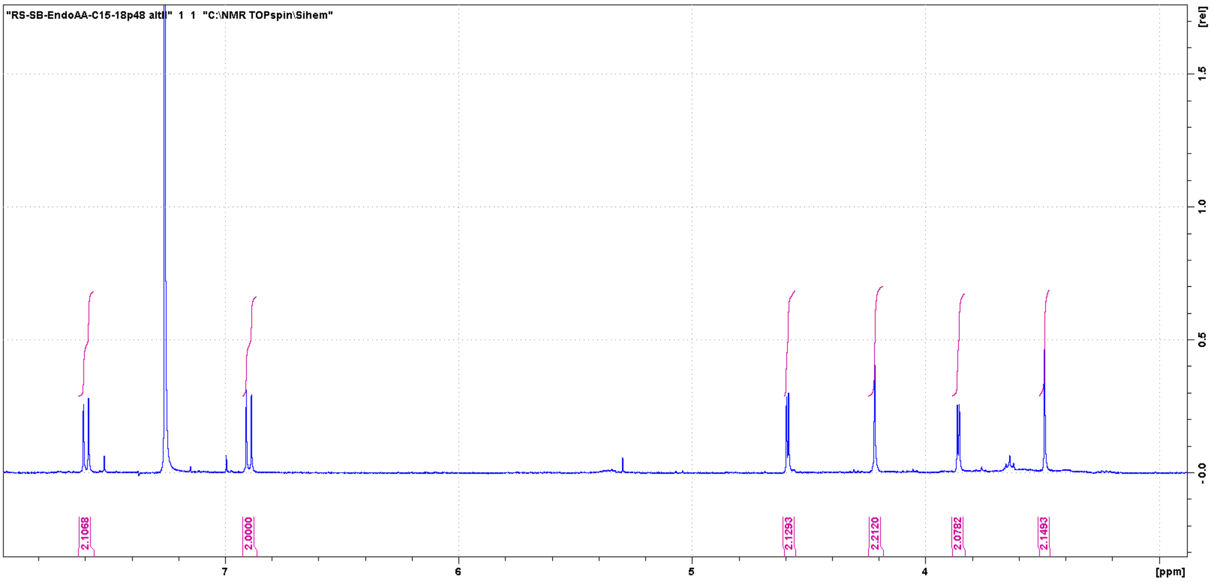

Figure S19: Zoom ^{1}H-NMR spectrum of compound **3,** altertoxin III, in $CDCl_3$ (400 MHz)

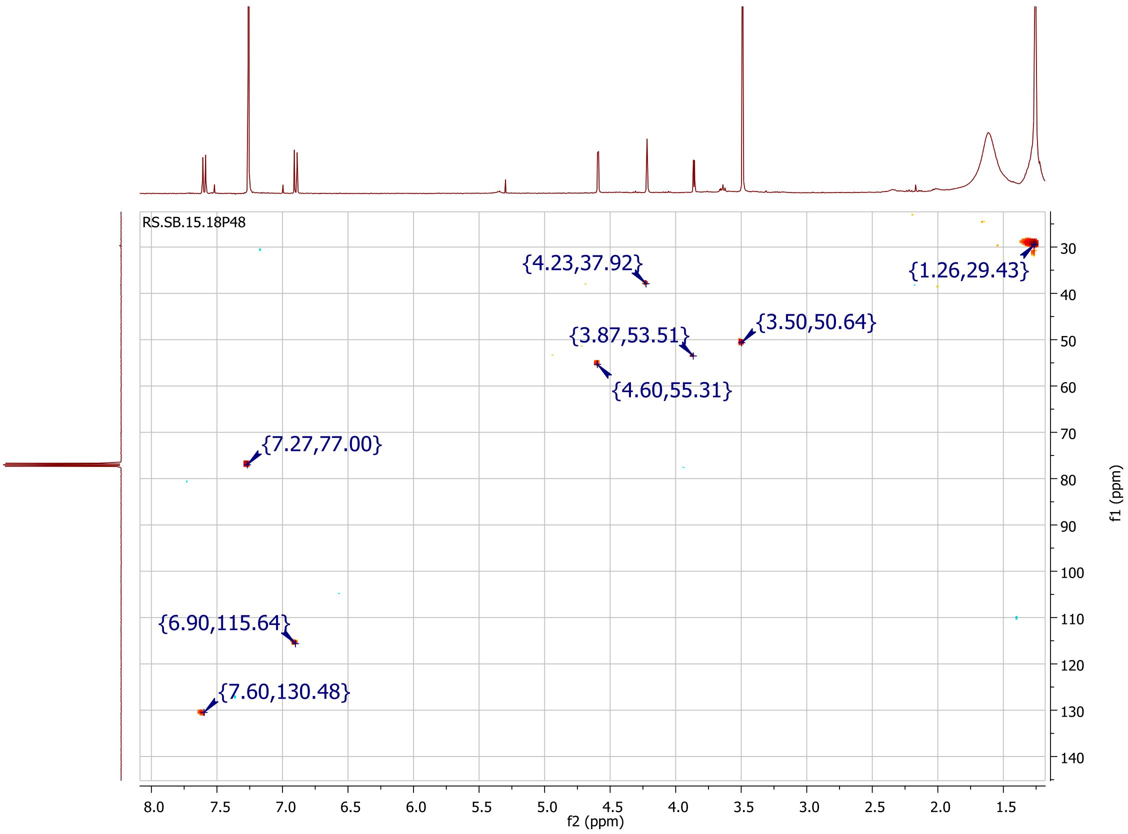

Figure S20: HSQC spectrum of compound **3,** altertoxin III, in $CDCl_3$

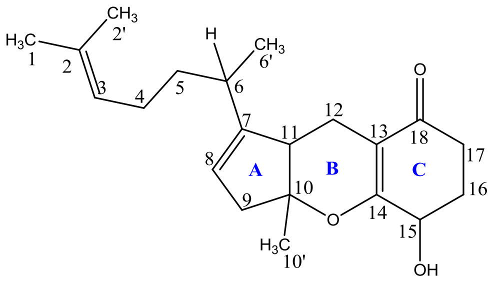

Figure S21: Chemical structure of compound **4,** Tricycloalternarene 3a

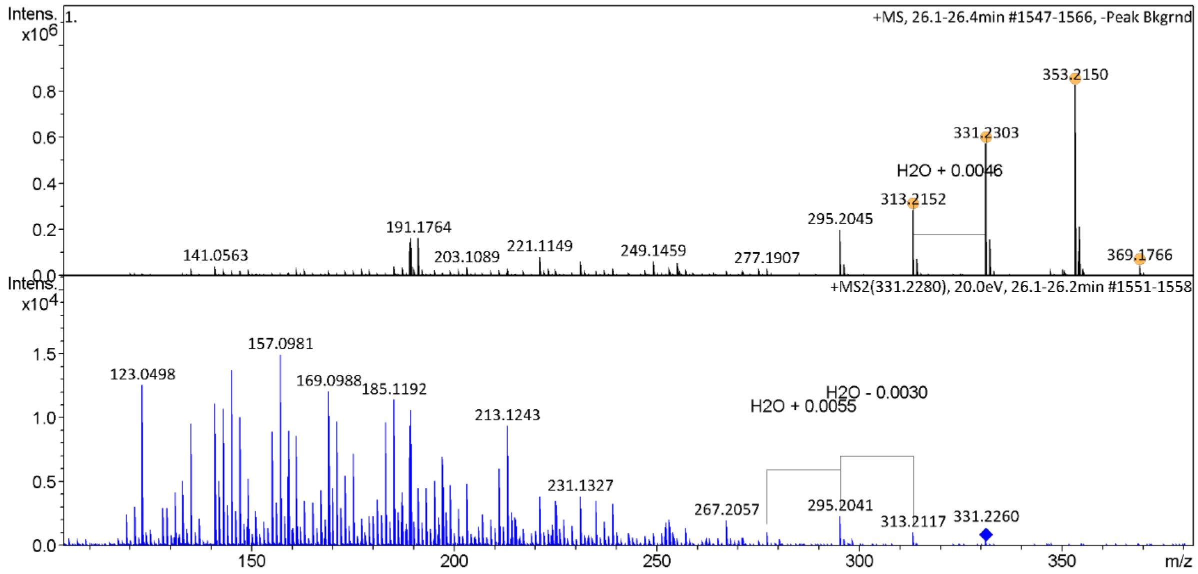

Figure S22: HR-ESI-MS spectrum of compound **4**, Tricycloalternarene 3a

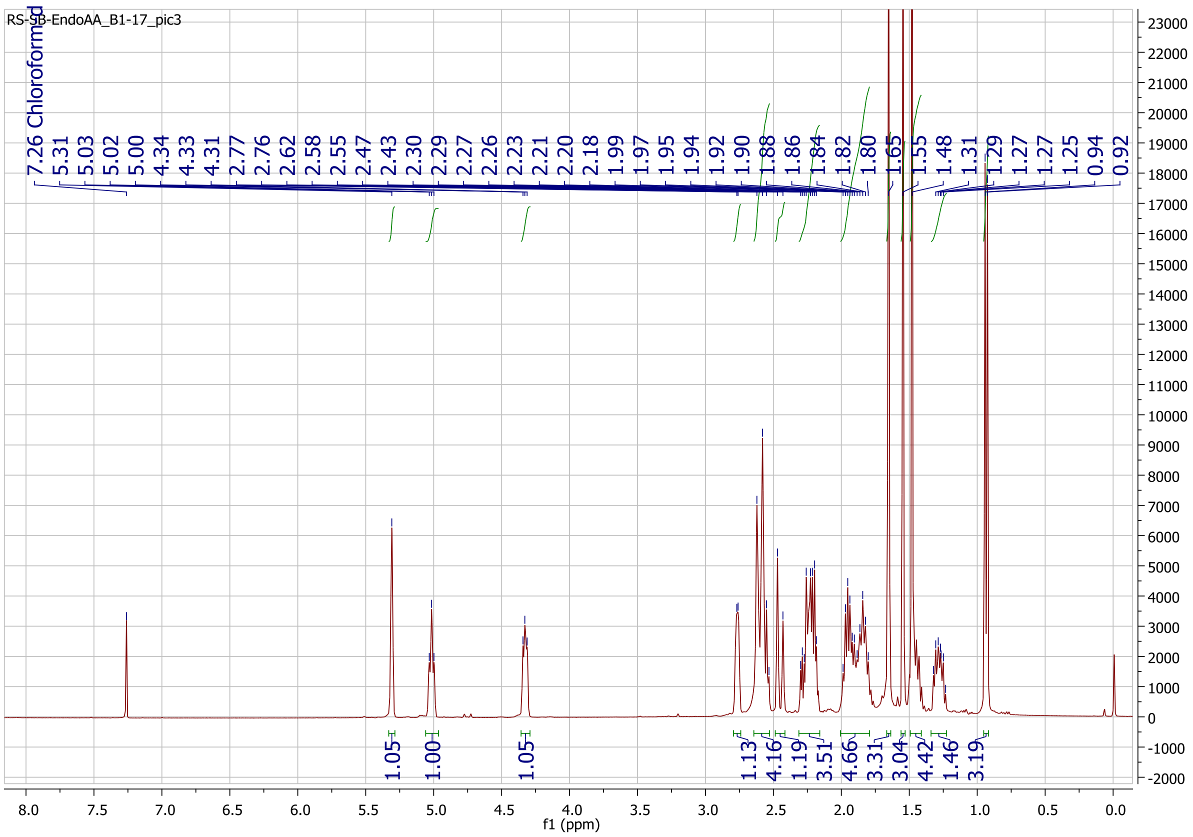

Figure S23: ^{1}H-NMR spectrum of compound **4,** Tricycloalternarene 3a, in $CDCl_3$ (400 MHz)

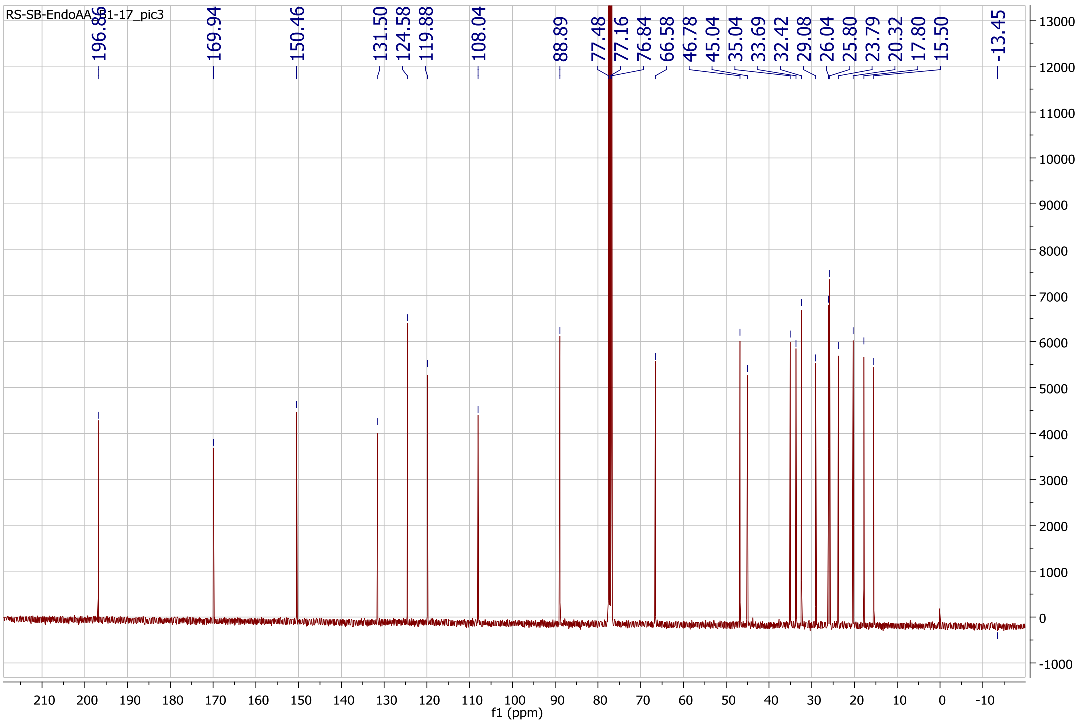

Figure S24: ^{13}C-NMR spectrum of compound **4,** Tricycloalternarene 3a, in $CDCl_3$ (100 MHz)

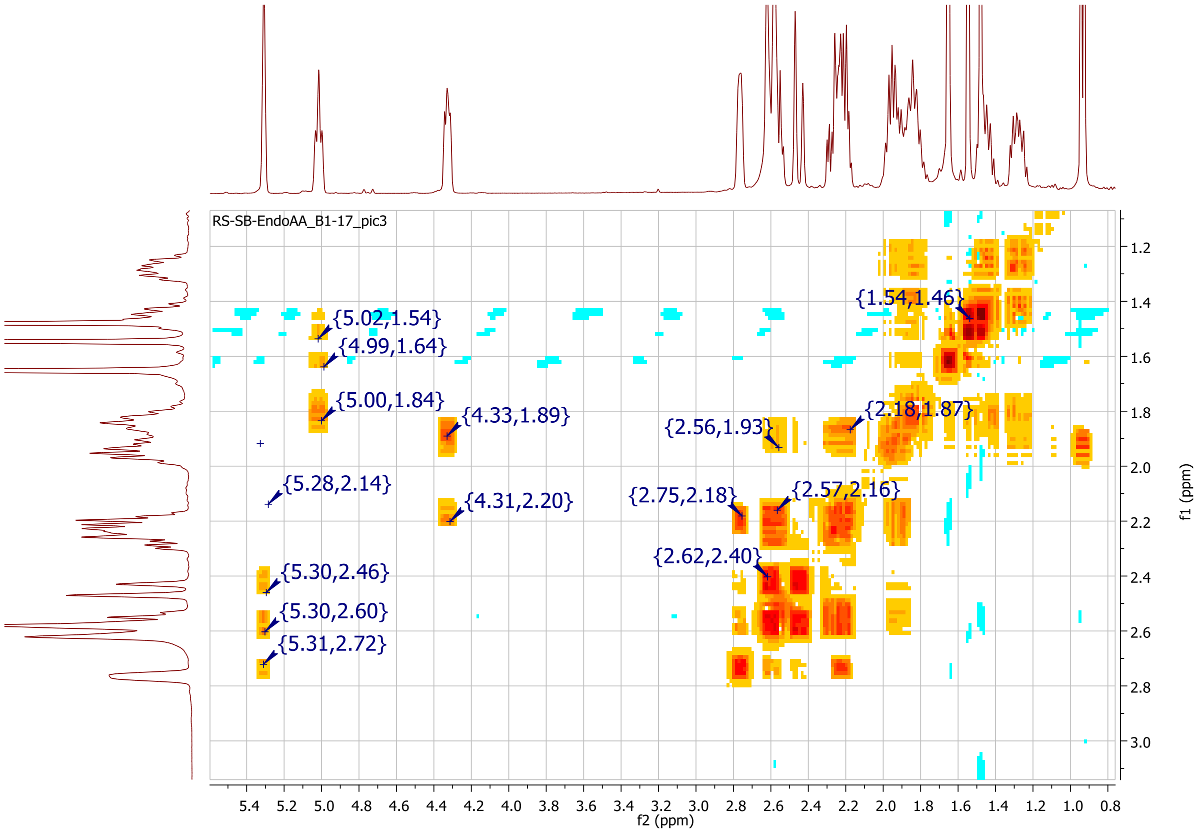

Figure S25: ^{1}H-^{1}H COSY spectrum of compound **4,** Tricycloalternarene 3a, in $CDCl_3$

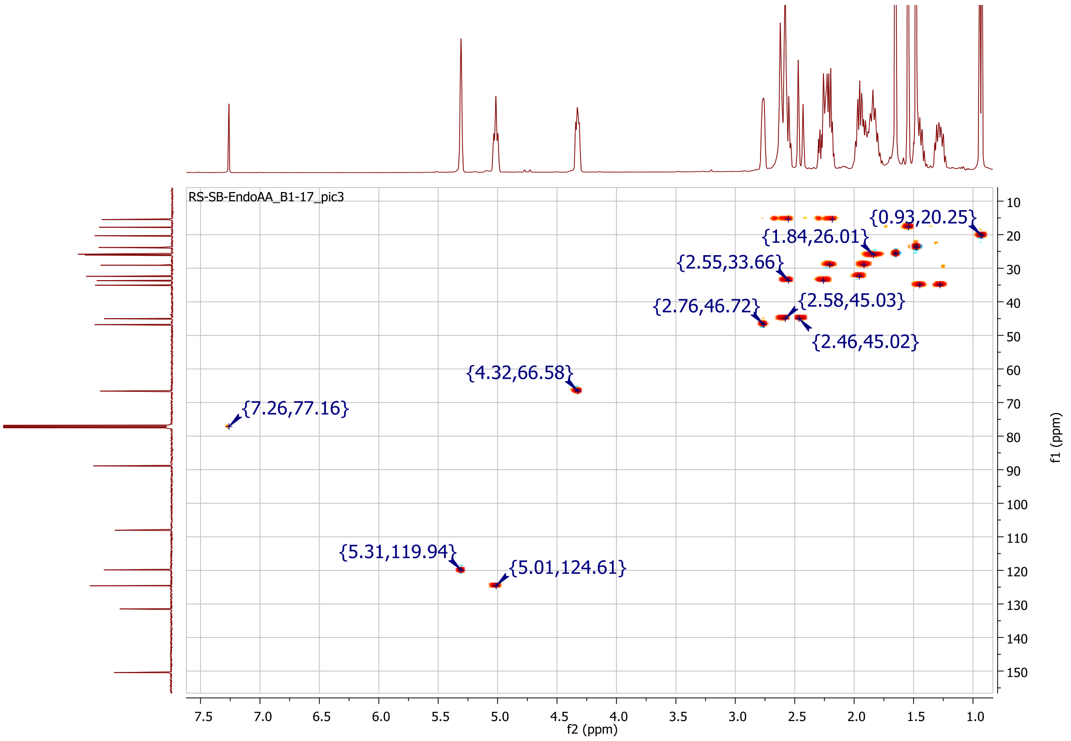

Figure S26: HSQC spectrum of compound **4,** Tricycloalternarene 3a, in $CDCl_3$

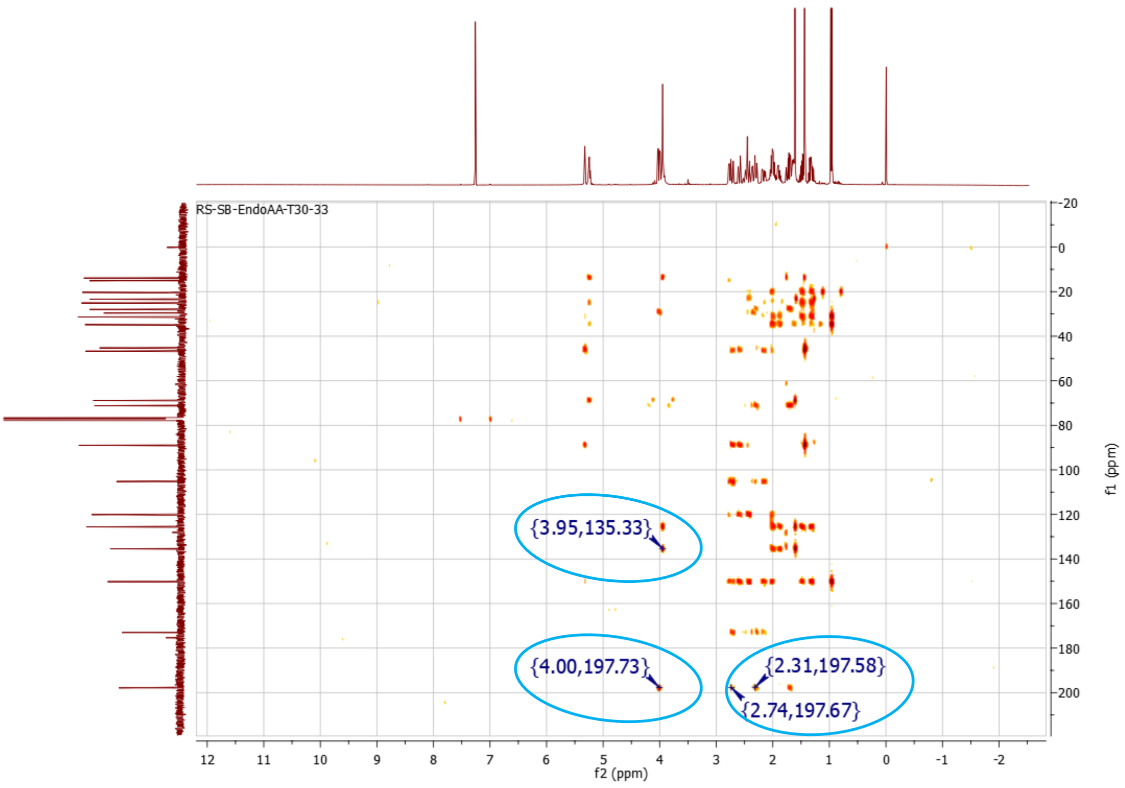

Figure S27: HMBC spectrum of compound **4,** Tricycloalternarene 3a, in $CDCl_3$

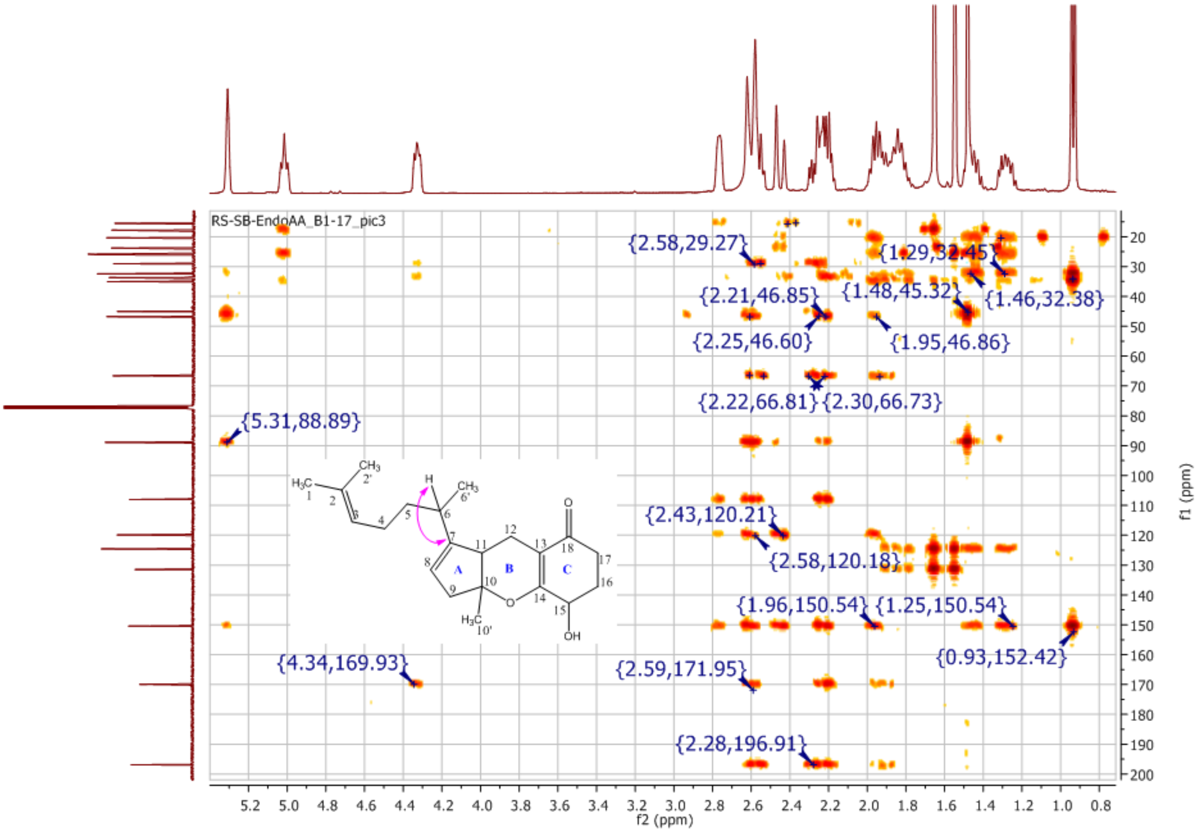

Figure S28: Zoom of HMBC spectrum of compound **4,** Tricycloalternarene 3a, in $CDCl_3$

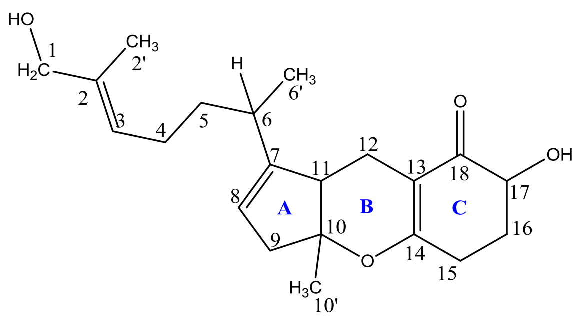

Figure S29: Chemical structure of compound **5,** Tricycloalternarene 2b

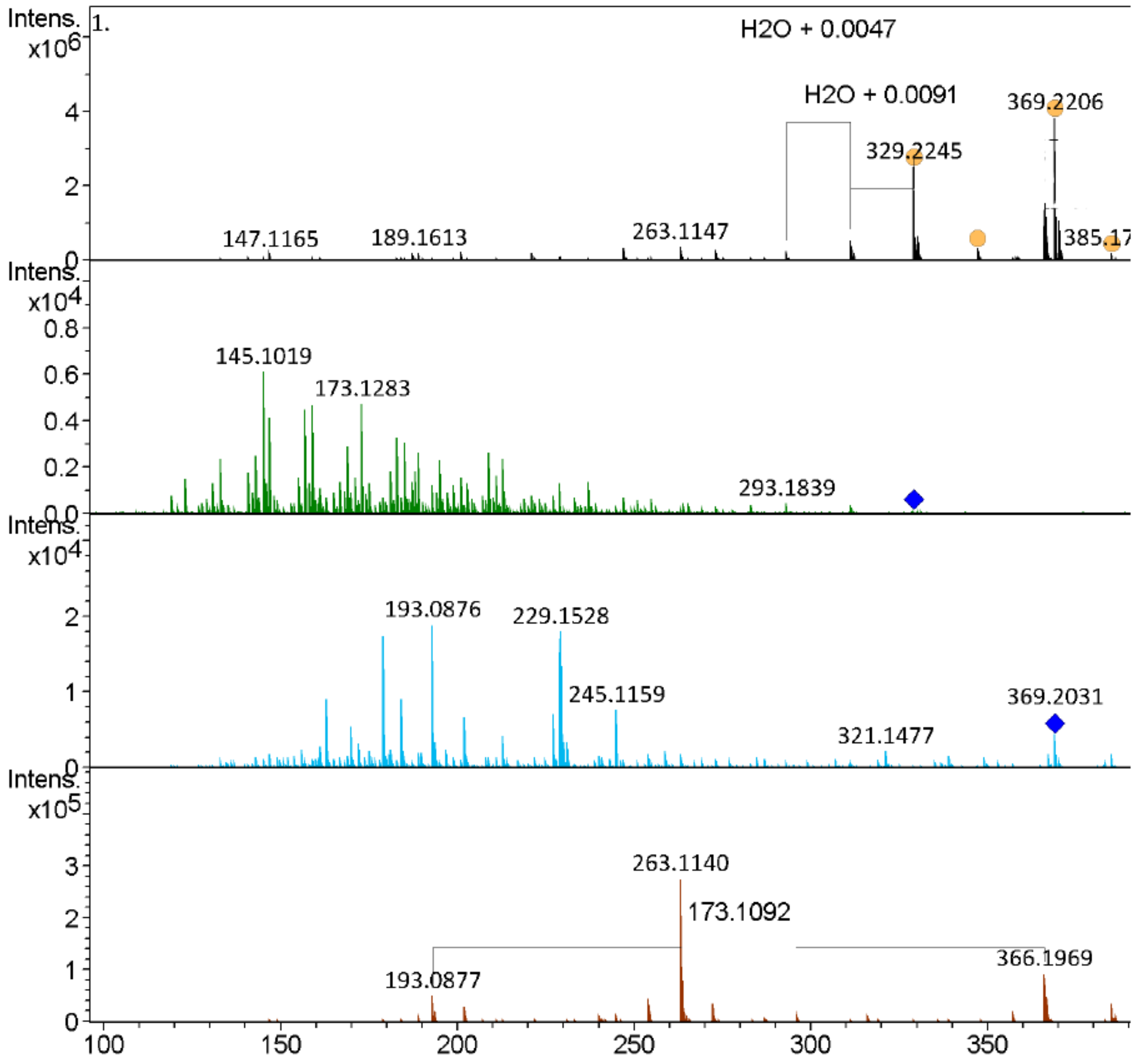

Figure S30: HR-ESI-MS spectrum of compound **5**, Tricycloalternarene 2b

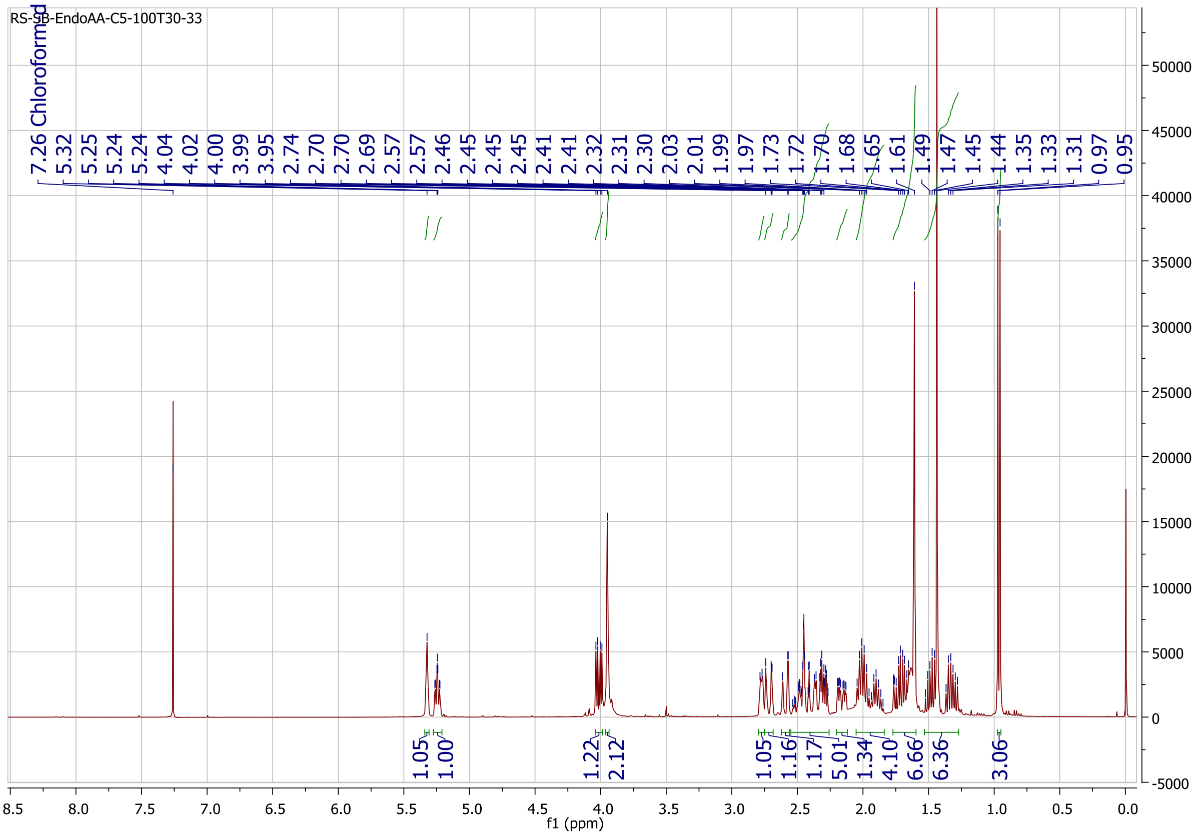

Figure S31: ^{1}H-NMR spectrum of compound **5,** Tricycloalternarene 2b, in $CDCl_3$ (400 MHz)

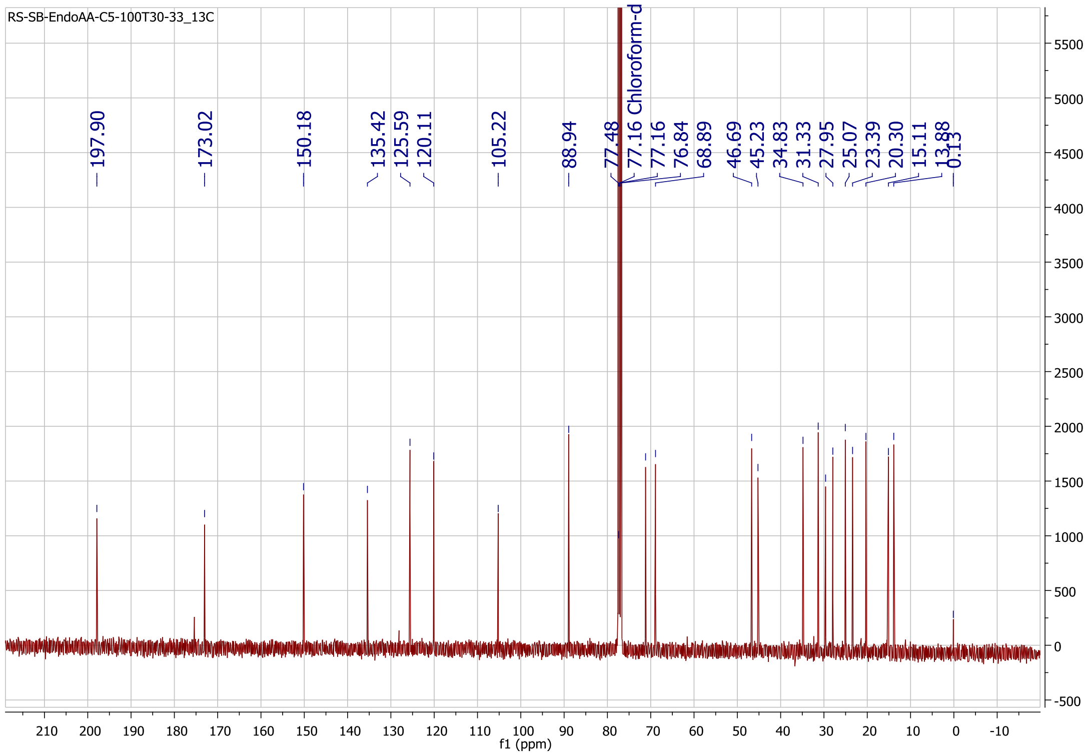

Figure S32: ^{13}C-NMR spectrum of compound **5,** Tricycloalternarene 2b, in $CDCl_3$ (100 MHz)

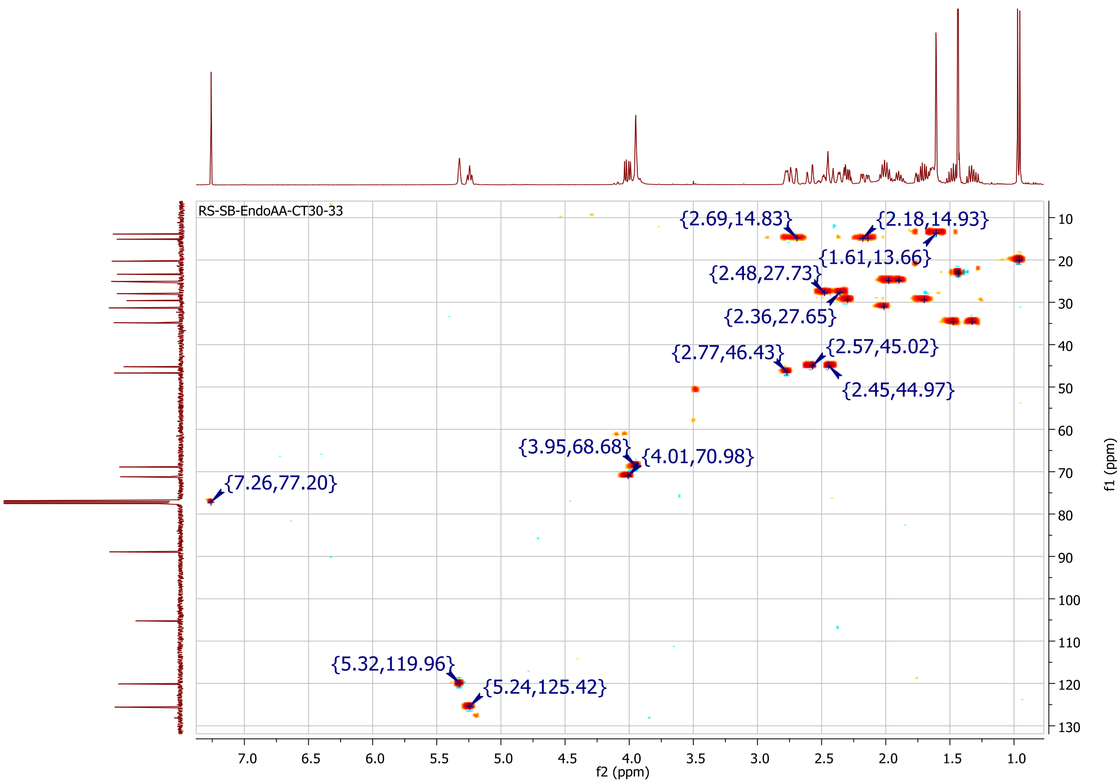

Figure S33: HSQC spectrum of compound **5,** Tricycloalternarene 2b, in $CDCl_3$

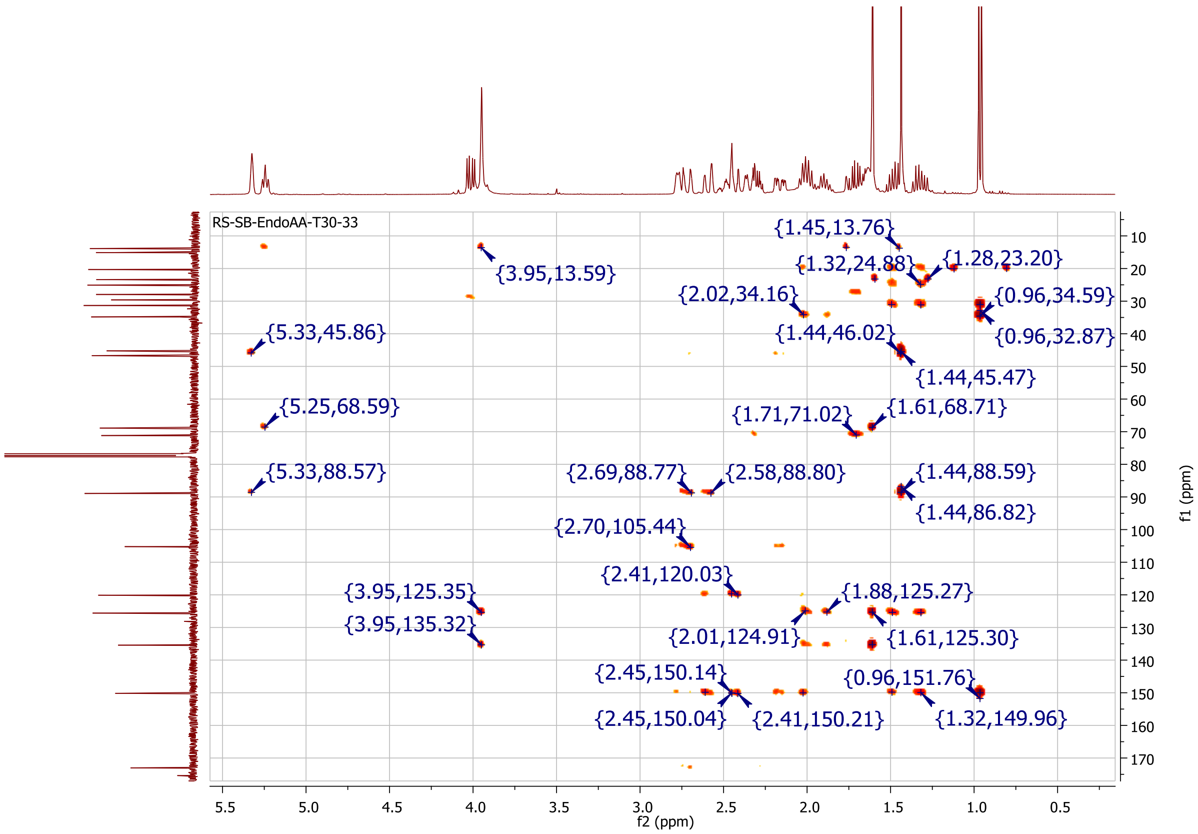

Figure S34: HMBC spectrum of compound **5,** Tricycloalternarene 2b, in $CDCl_3$

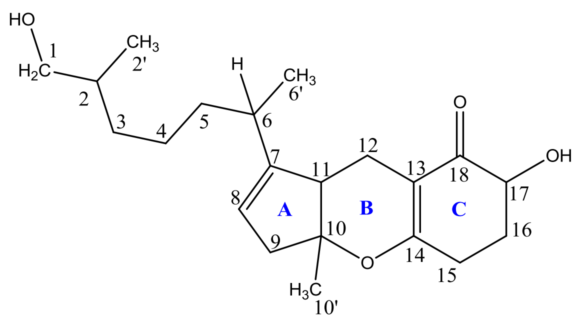

Figure S35: Chemical structure of compound **6,** Tricycloalternarene 1b

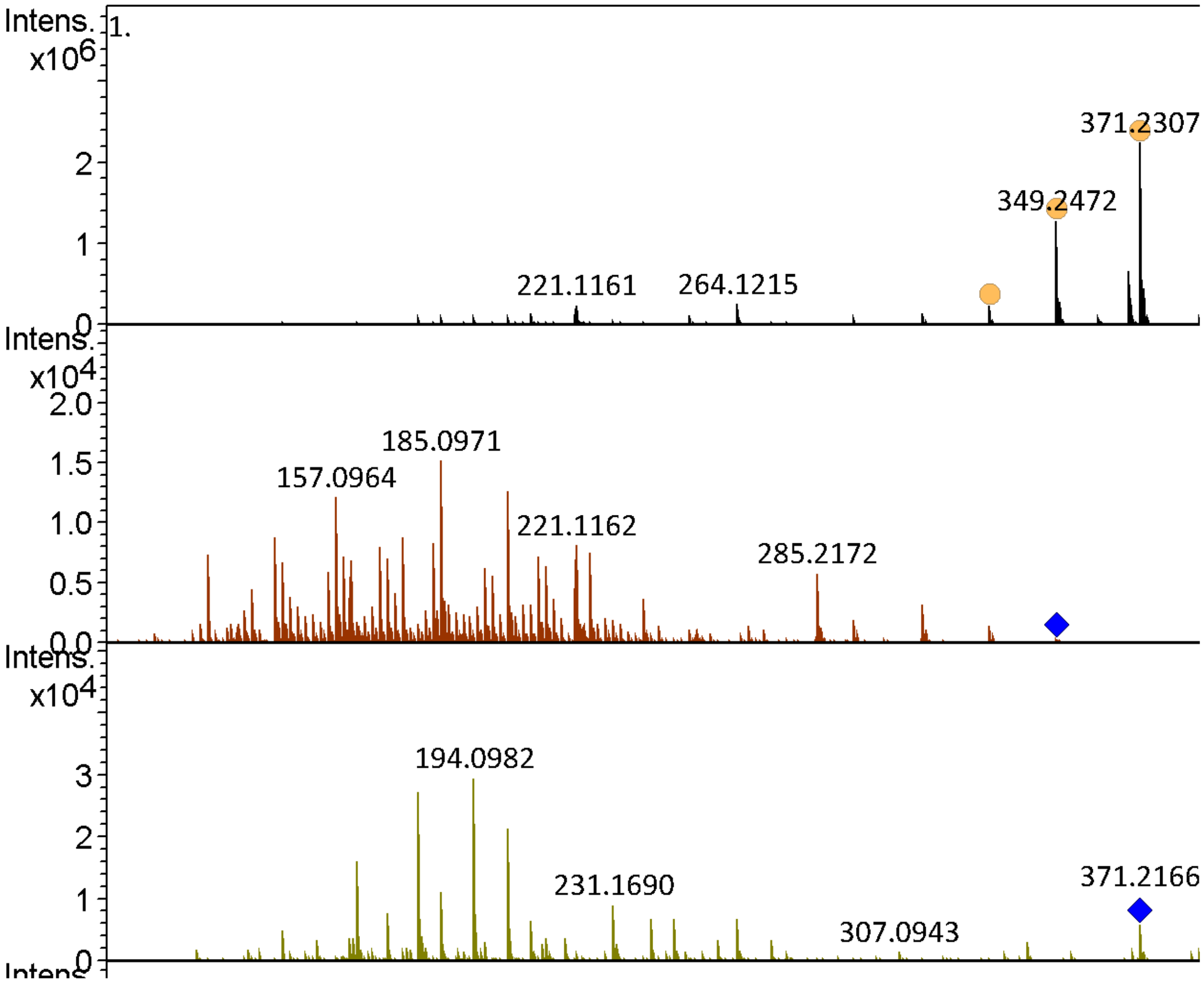

Figure S36: HR-ESI-MS spectrum of compound **6**, Tricycloalternarene 1b

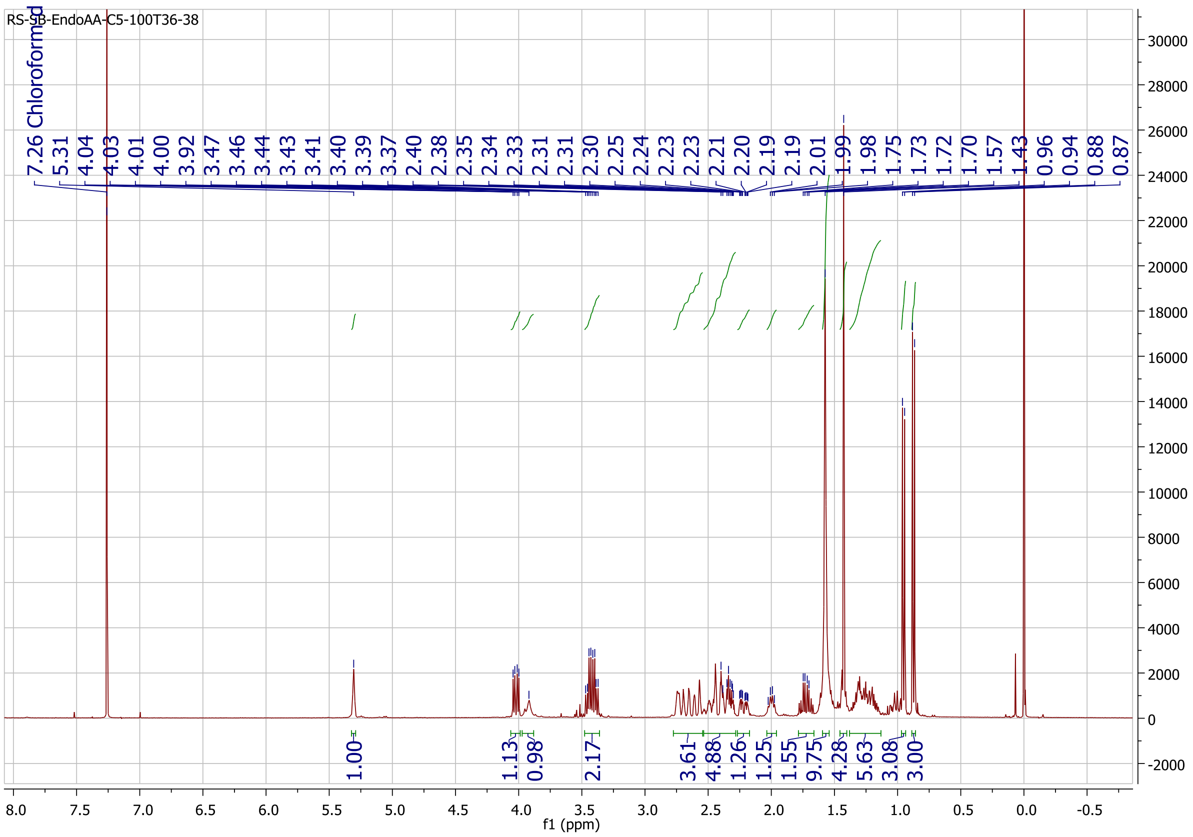

Figure S37: ^{1}H-NMR spectrum of compound **6,** Tricycloalternarene 1b, in $CDCl_3$ (400 MHz)

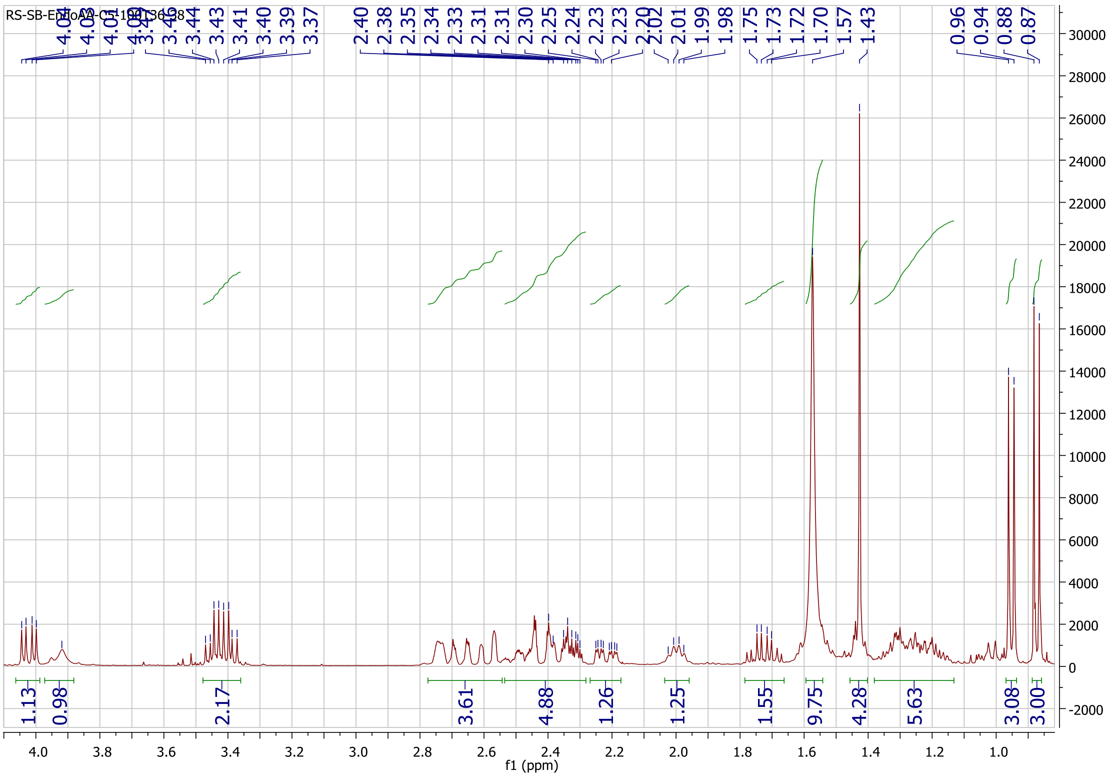

Figure S38: Zoom ^{1}H-NMR spectrum of compound **6,** Tricycloalternarene 1b, in $CDCl_3$ (400 MHz)

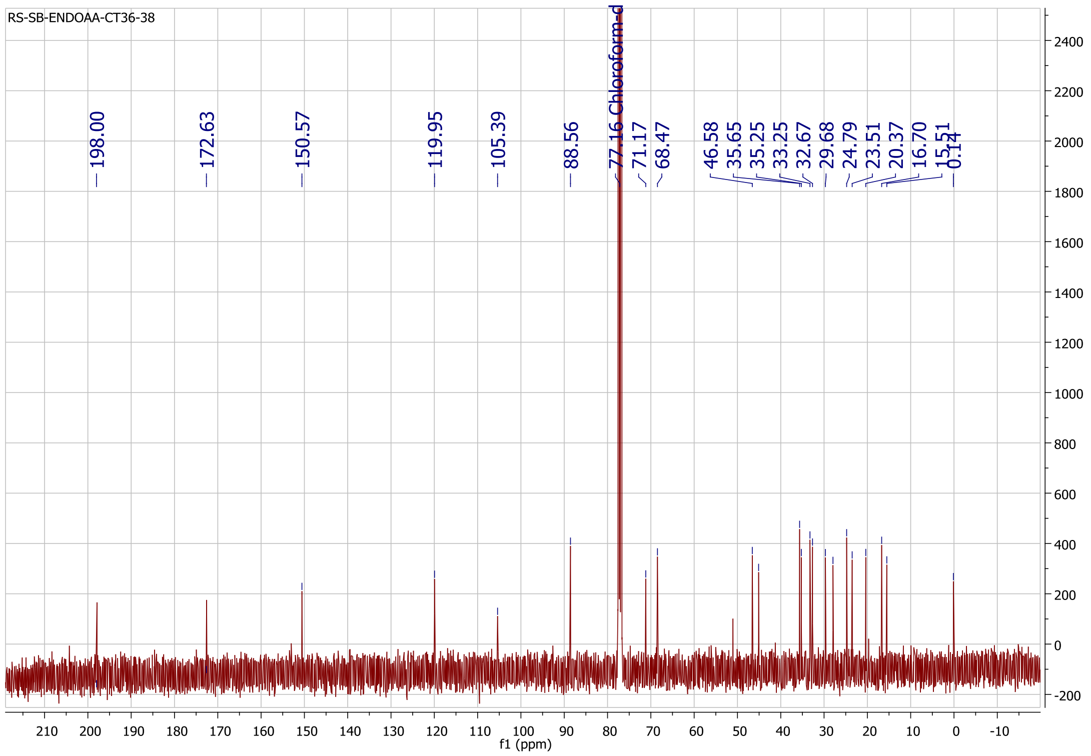

Figure S39: ^{13}C-NMR spectrum of compound **6,** Tricycloalternarene 1b, in $CDCl_3$ (100 MHz)

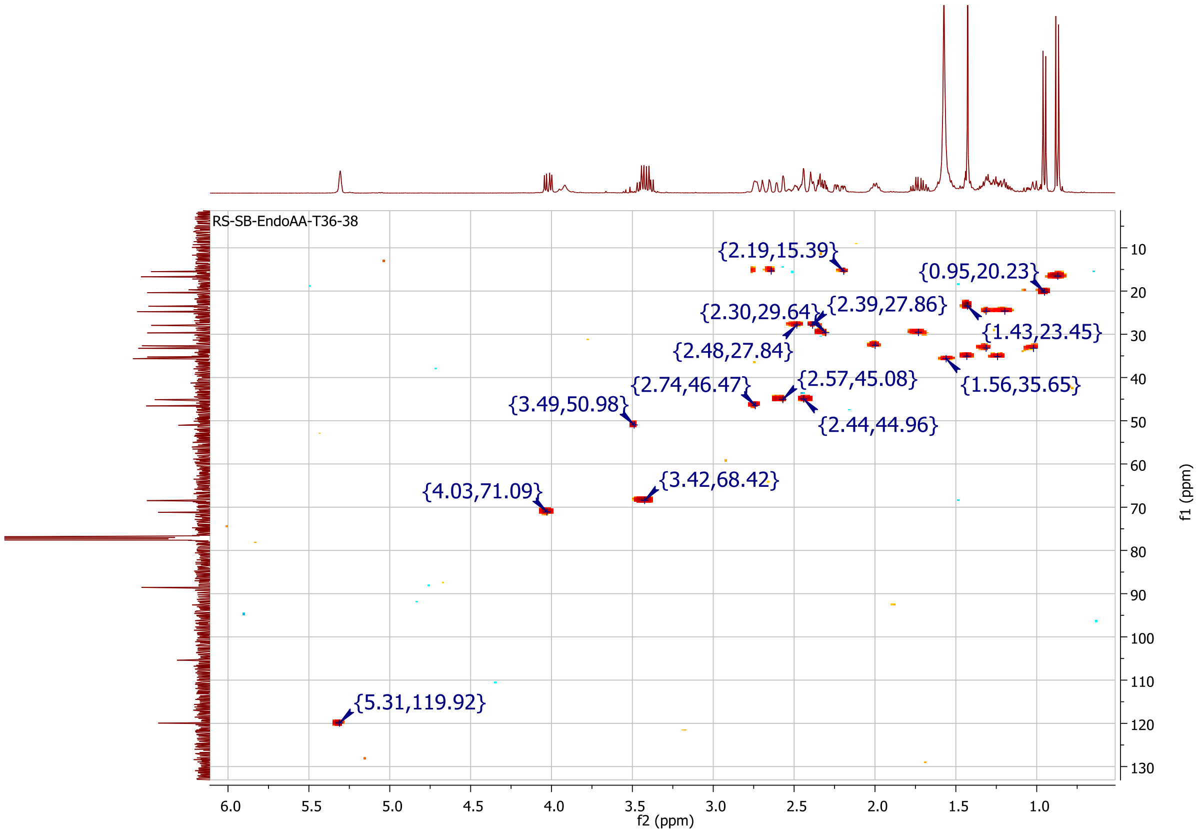

Figure S40: HSQC spectrum of compound **6,** Tricycloalternarene 1b, in $CDCl_3$

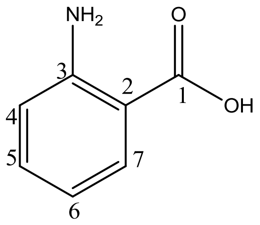

Figure S41: Chemical structure of compound **7,** Anthranilic acid

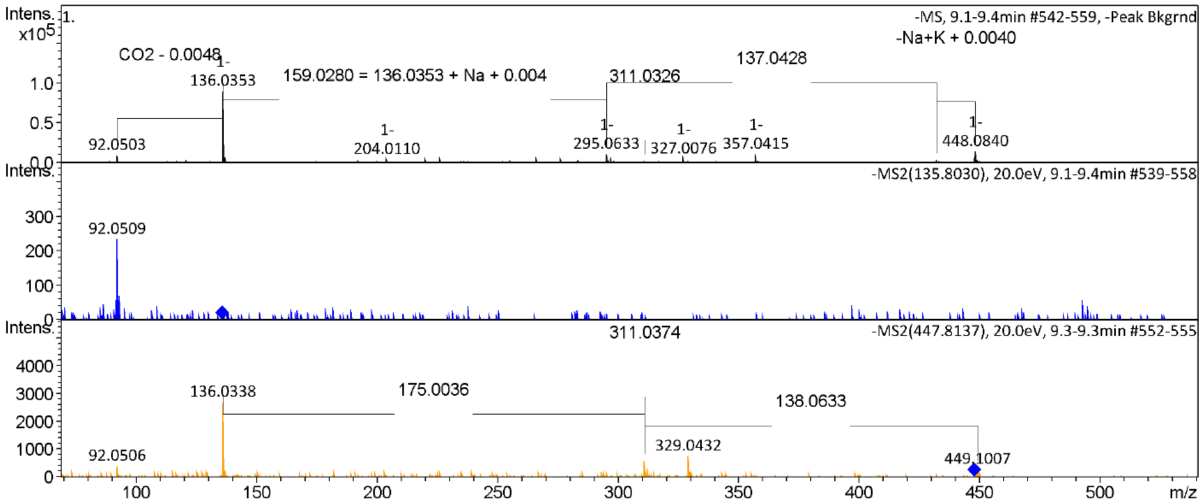

Figure S42: HR-ESI-MS spectrum of compound **7**, Anthranilic acid

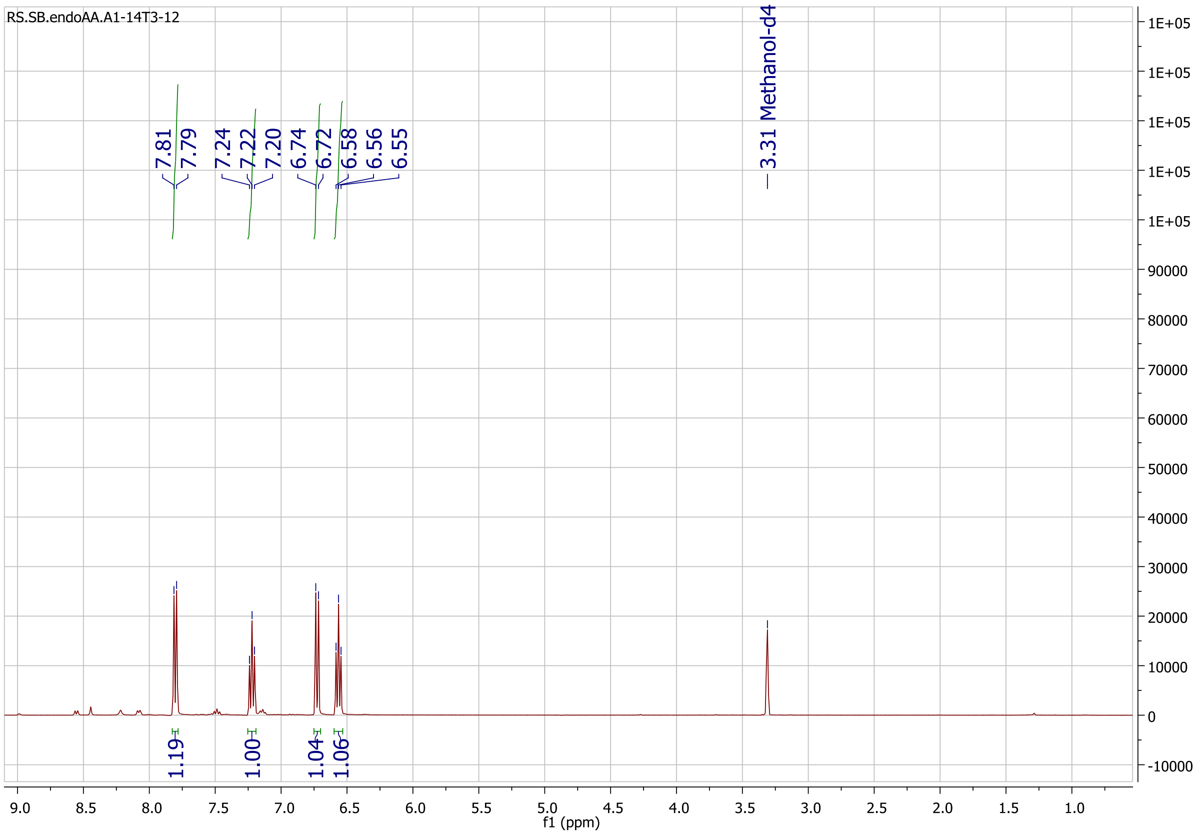

Figure S43: ^{1}H-NMR spectrum of compound **7,** Anthranilic acid, in CD_3OD (400 MHz)

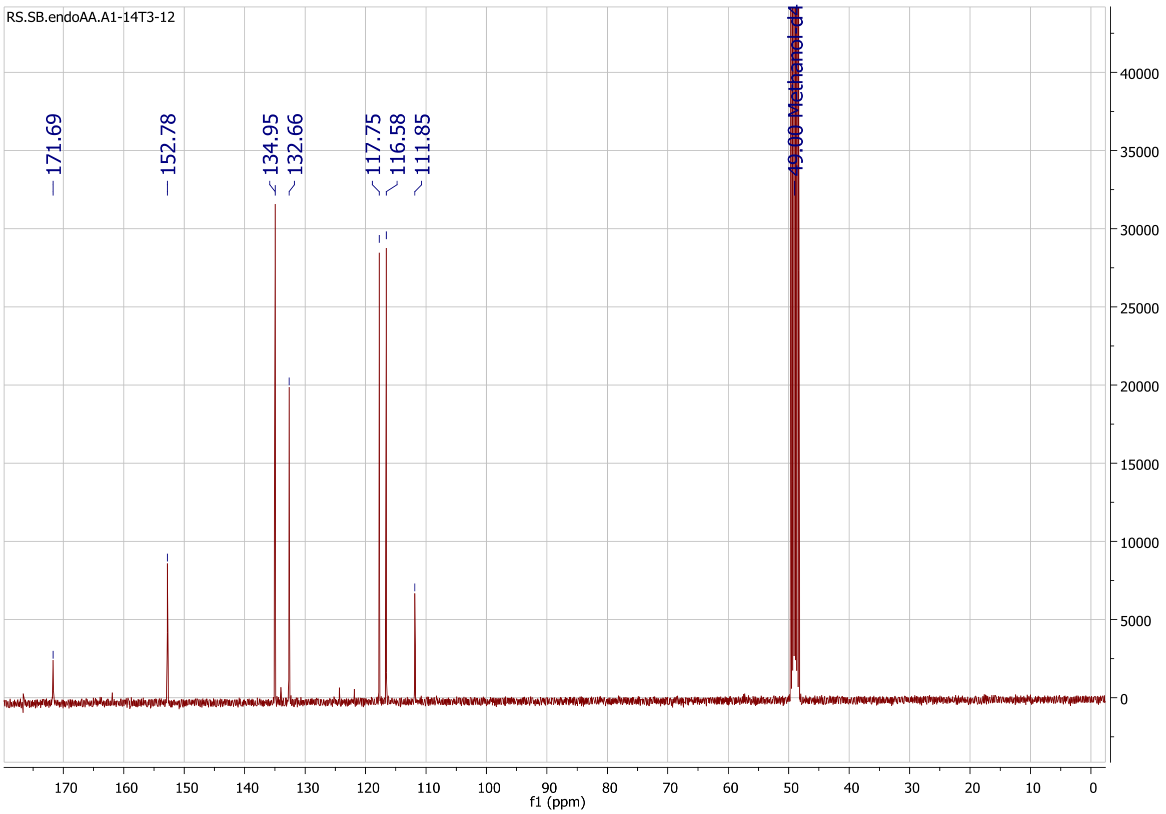

Figure S44: ^{13}C-NMR spectrum of compound **7,** Anthranilic acid, in CD_3OD (100 MHz)

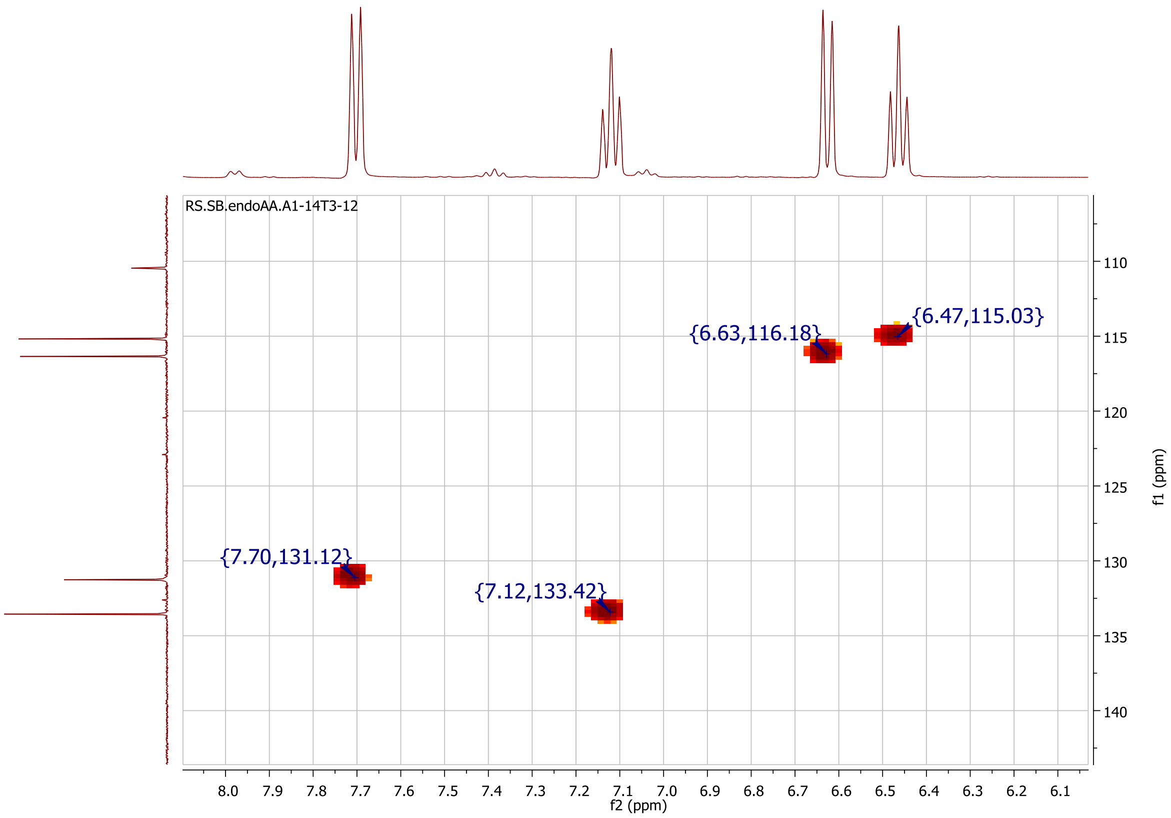

Figure S45: HSQC spectrum of compound **7,** Anthranilic acid, in CD_3OD

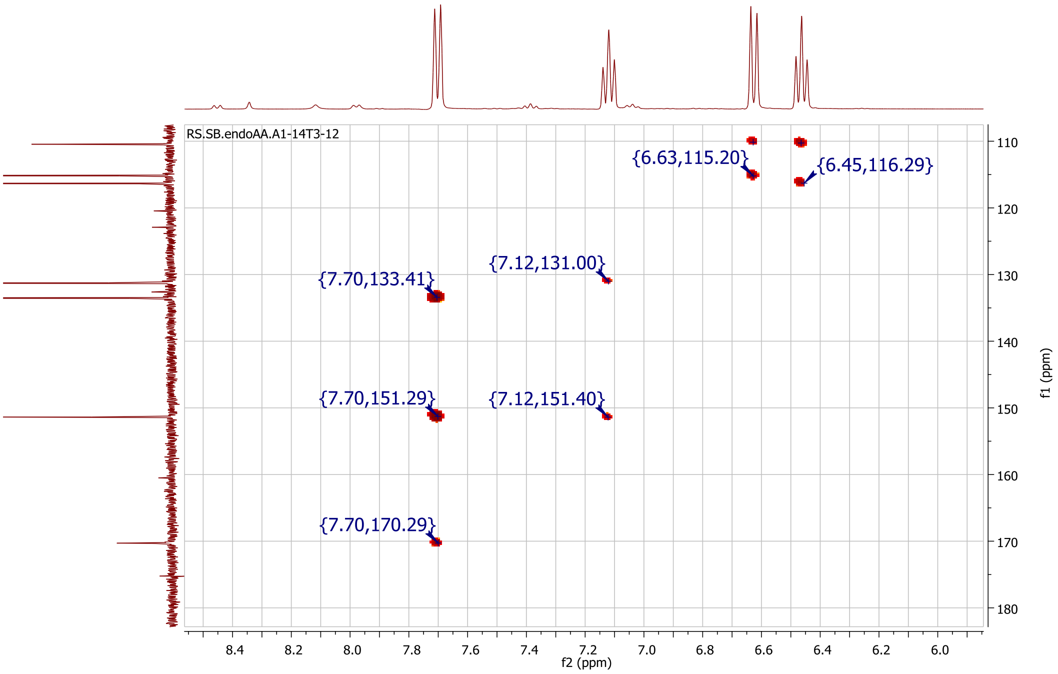

Figure S46: HMBC spectrum of compound **7,** Anthranilic acid, in $CDCl_3$

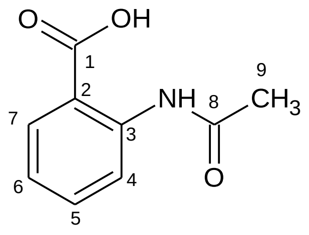

Figure S47: Chemical structure of compound **8,** *o*-acetamidobenzoic acid

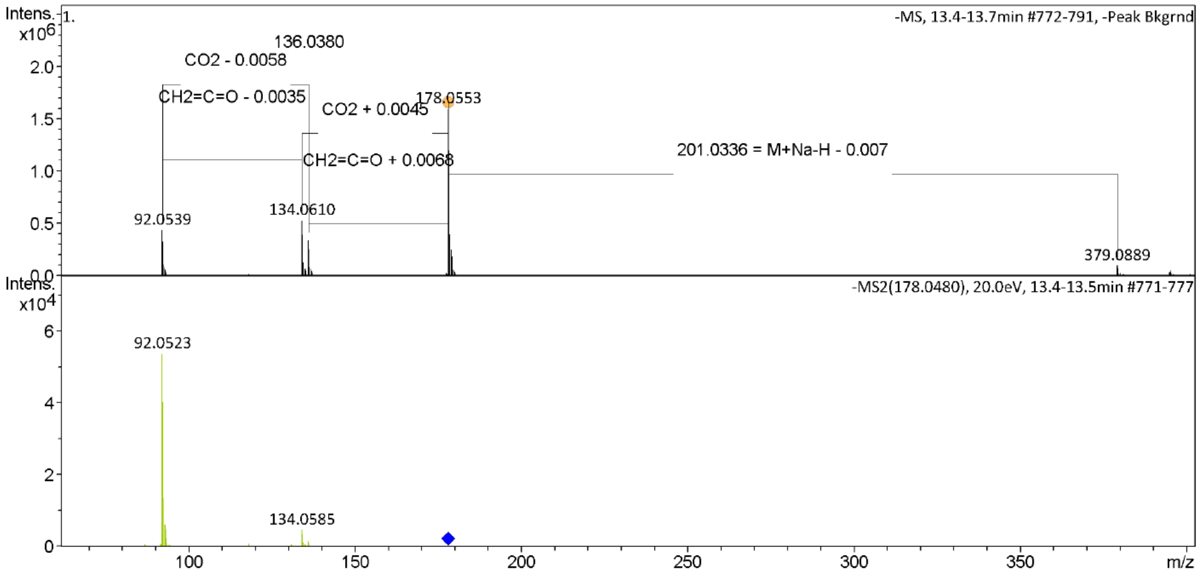

Figure S48: HR-ESI-MS spectrum of compound **8**, *o*-acetamidobenzoic acid

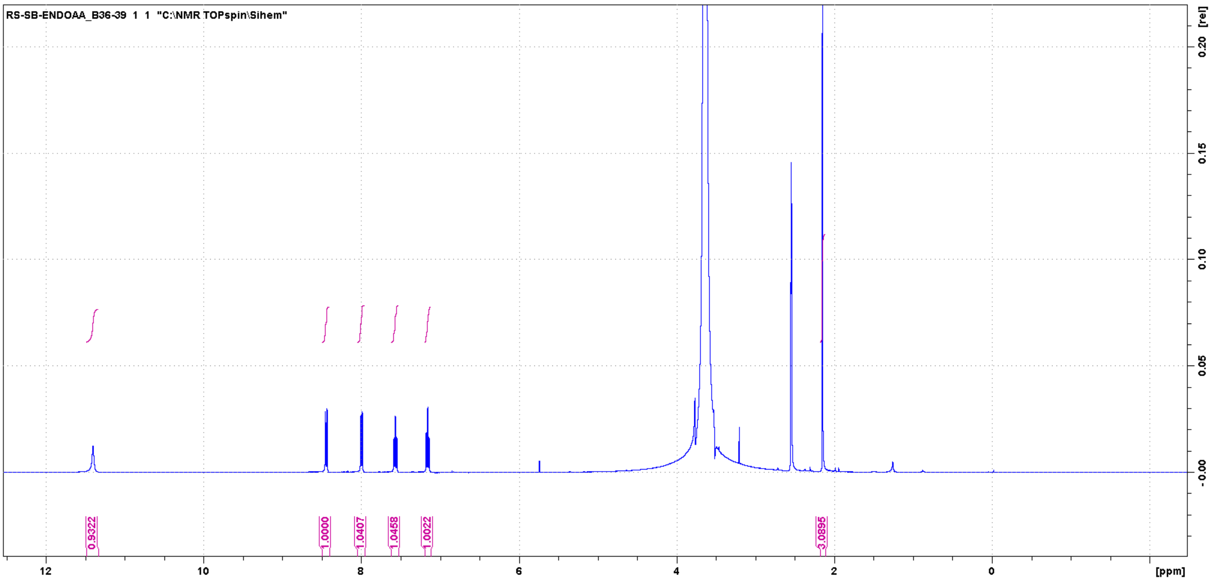

Figure S49: ^{1}H-NMR spectrum of compound **8,** *o*-acetamidobenzoic acid, in DMSO-d_6 (400 MHz)

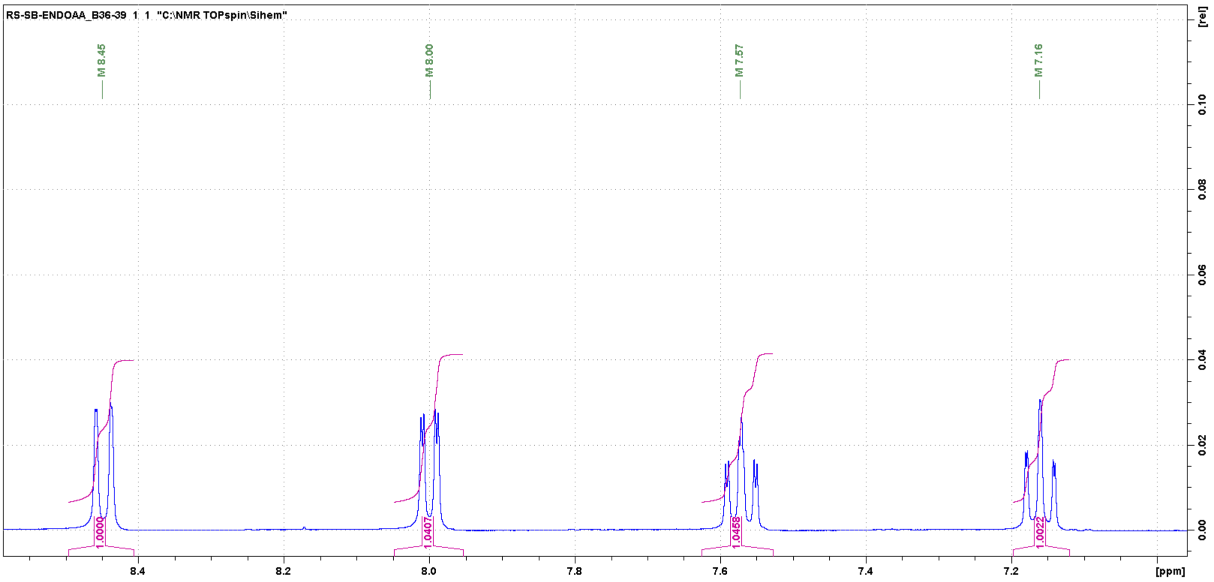

Figure S50: Zoom ^{1}H-NMR spectrum of compound **8,** *o*-acetamidobenzoic acid, in DMSO-d_6 (400 MHz)

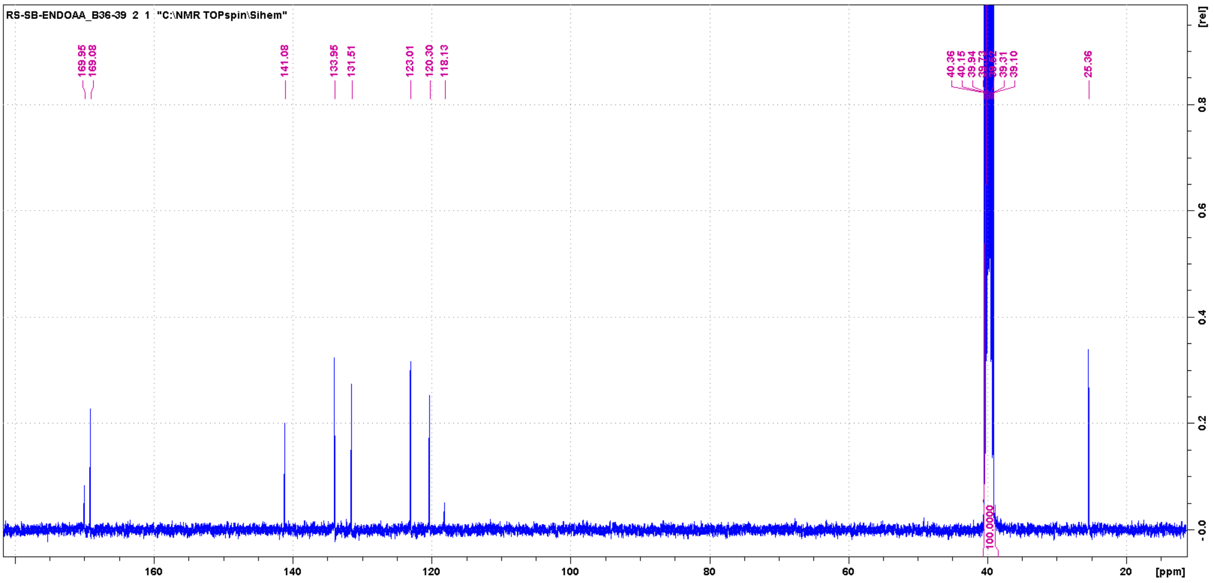

Figure S51: ^{13}C-NMR spectrum of compound **8,** *o*-acetamidobenzoic acid, in DMSO-d_6 (100 MHz)

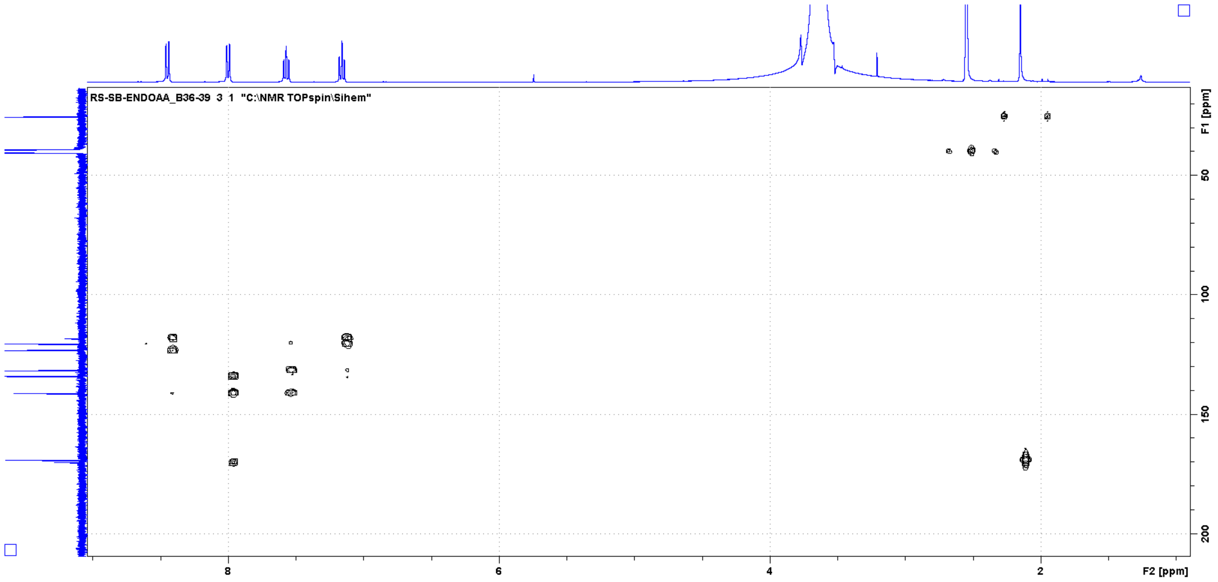

Figure S52: HMBC spectrum of compound **8,** *o*-acetamidobenzoic acid, in DMSO-d_6
